# Review of the effect of atrazine on the HPG axes and steroidogenic pathways in males: relevance for testicular and prostate cancer

**DOI:** 10.3389/ftox.2025.1702389

**Published:** 2026-03-11

**Authors:** Ralph L. Cooper, James W. Simpkins, Charles Breckenridge

**Affiliations:** 1 Quality Scientific Solutions, LLC, Waynesboro, VA, United States; 2 Department of Neuroscience, West Virginia University, Morgantown, WV, United States

**Keywords:** atrazine, carcinogenicity, estrogen, FSH, HPG axis, LH, prolactin, testosterone

## Abstract

Subacute, subchronic, and chronic rodent studies on atrazine were evaluated to determine the no observed adverse effect levels (NOAEL) for male reproductive toxicity and carcinogenicity. *In vitro* studies were also evaluated for the effect of atrazine on molecular pathways related to male reproductive function and potential mechanisms for cancer. Gestational exposure to high doses of atrazine delayed male development post-partum. The NOAEL in male offspring following perinatal exposure of the dam from PND 1-4 was ≥ 25 mg/kg/day. The NOAEL for an increase in prostate inflammation on PND 120 was 12.5 mg/kg/day. Exposure of peripubertal males to atrazine reduced serum and intratesticular testosterone levels, decreased gonadal and prostate gland weights, and decreased sperm count and motility. The NOAELs were approximately 25 mg/kg. Increased plasma estrone and estradiol levels at atrazine doses of 50–200 mg/kg/day were associated with elevated aromatase expression and impaired fluid absorption in the efferent ductules of the testes. Oxidative stress, detected *in vivo* and *in vitro* at high atrazine doses, was associated with reduced testosterone levels, increased cytokine activity, and inflammation. The NOAEL for these effects was 25 mg/kg/day. Lifetime doses up to 25 mg/kg/day of atrazine did not result in an elevated incidence of prostate or testicular cancer in rodents. An increased incidence of interstitial cell tumors in male rats (53 mg/kg/day) was not significant after adjusting for the increased survival. Finally, epidemiological studies did not provide any consistent evidence of a causal association between atrazine exposure and testicular or prostate cancer in men.

## 1 Introduction

**FIGURE 1:**
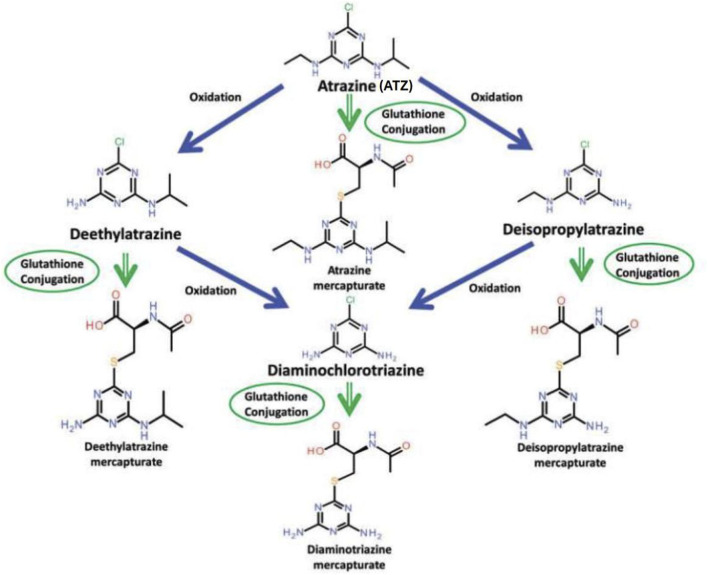
Chlorotriazine metabolism (From Campbell et al. (2016)). Figure 1 displays the oxidative metabolism of atrazine, resulting in the formation of the following three dealkylated chlorometabolites: DEA (deethylatrazine, DIA (deisopropylatrazine and DACT (diaminochlorotriazine). Atrazine and its chlorometabolites also undergo glutathione conjugation, leading to the formation of their respective mercapturates.

The purpose of this review is to assess the effects of atrazine and its chloro-metabolites DEA, DIA, and DACT (Figure 1) on the hypothalamic-pituitary-gonadal (HPG) axis in males to determine if plausible biological mechanisms could lead to cancer in humans at exposure levels found in food, water, or the workplace. This review complements the one by Cooper et al. (2026) on the effects of atrazine on the HPG axis in females.

To identify relevant publications, independent searches of PubMed and Google Scholar were conducted using the term “atrazine,” with further refinements focusing on topics pertinent to this manuscript (e.g., testes, prostate, testosterone, inflammation, cancer). We also obtained a copy of an electronic Endnotes database of published studies on atrazine maintained by Syngenta Crop Protection Inc. This database, containing 39,537 peer-reviewed journal articles, was compiled by Syngenta over the past 30 years as of November 2024. Syngenta searched the public literature using Agricola, CiNii, EMBASE, PASCAL, SciFinder, Toxline, WorldCat, PubMed, BIOSIS Previews, and CAB Abstracts. QS3 searched this library using EndNote. Additionally, QS3 conducted searches of the scientific literature via PubMed and Google Scholar for other topics (keywords, authors, etc.) related to this manuscript’s subject matter. Furthermore, studies cited in other reviews (Wirbisky and Freeman, 2015; Abarikwu et al., 2023; Guimaraes-Ervilha et al., 2025; Holliman et al., 2025) were also evaluated.

We summarized the data from relevant studies in tables, which were created by one author (CB) and verified by a second (RC). Summaries of regulatory studies and epidemiology studies were created by subcontractors, and verified by CB. Statistical analyses of epidemiological studies were conducted by a subcontractor and graphical presentations were created by a second subcontractor. CB verified the accuracy of these data. Adverse outcome pathways were prepared by CB and double-checked by RLC and JWS.

For each study whose data was summarized in tables, we provided the dose or concentration levels, duration of exposure and, if appropriate, the day or time after the initiation of dosing when the parameter listed in the table were assessed. Parameters where there were no statistically significant differences between control and any treated group were recorded as having no effect of treatment (NE). The no observed adverse effect level (NOAEL) in these cases was the highest dose tested. If there were statisically significant differences between the control and treated groups reported by the authors of the paper, then the lowest dose at which the effect was first seen was represented in the table with that dose. An up-arrow or down-arrow indicated the direction of the statistically significant difference between the treated and the control groups. In these instances, the lowest observed adverse effect level (LOAEL) was the lowest dose at which the effect was seen. The NOAEL was the dose just below the LOAEL dose, if there was such a dose in the study. In some cases, we reported absolute values for some parameters where there was a row of data for provided in the study for the control and treated groups. The study by Stanko et al. (2010), summarized in Table 3, is an example of such a study.

Studies summarized by presenting graphical representations of the data in Supplemental Figures 1-8 typically provided, the mean ± SEM, the number of animals/group an an indication of which groups were statisically significantly different from each other and the p value for each contrast. The NOAELS and LOAELS were obtained from these plots and corroborated by statements made by the authors in the text of their publication. QS3 did not perform any ad hoc statistical analysis of data from published studies, except in the case of the meta-analysis of the epidemiology study data (Table 7 and Supplemental Table 7).

In Sections 3, 4, we summarized the effects of atrazine on the HPG axis, including effects on:

1. LH and the role that LH plays in regulating steroidogenesis via LHR receptors in Leydig cells,
2. Endogenous biotransformation of androgens to estrogens,
3. FSH secretion and its role in the regulation of spermatogenesis via FSHR receptors in Sertoli cells,
4. The modulation of the tuberoinfundibular dopaminergic (TIDA) neurons during development and possible dysregulation of prolactin following gestational or early lactational exposure.
5. Prolactin-induced prostatic inflammation.

We also summarized the effects of atrazine on the HPA axis and the potential interaction between the HPA and HPG axes (Section 5). This included an evaluation of proposed adverse outcome pathways (AOPs) appearing in the published literature relating to the effect of atrazine on the HPA and HPG axes, as well as the effects of atrazine on cellular processes that are considered hallmarks of carcinogens (Smith, 2019).

We reviewed and graphically summarized postulated AOPS discussed in the published literature that could lead to a disruption of the hormonal control of male reproductive function, the initiation or progression of testicular or prostate inflammation leading to cancer. These included:

1. Steroidogenesis and the alteration of testosterone synthesis within the testes.
2. Phosphodiesterase (PDE) inhibition leads to increased aromatase activity, resulting in increased conversion of androgens to estrogens.
3. Mitochondria redox imbalance resulting from postulated electrophilic interference with electron transport leading to increased production of reactive oxygen species (ROS).
4. Dose-dependent increase in oxidative stress in Leydig cells leading to cytotoxicity, cell death, and impaired gonadal function.
5. Induction of prostatic inflammation by the activation of the AR, GPER-Gai-EGFR/PI3K/ERK, or NF-κB pathway as the nuclear transcriptional endpoint.

Epigenetics was only briefly discussed because of a lack of atrazine-specific data. Genotoxicity (Brusick, 1994; Hauswirth and Wetzel, 1998; Jowa and Howd, 2011; Morris-Schaffer et al., 2025) and the effects of atrazine on the immune system (Breckenridge et al., 2026), reproduction (DeSesso et al., 2004), and development (Goodman et al., 2014; Scialli et al., 2014) have been reviewed elsewhere by others.

We summarized histopathological findings in the reproductive organs from sub-chronic and chronic studies on male rats. We also provided tumor incidence data from carcinogenicity studies to determine if lifetime exposure to atrazine increased the occurrence of testicular or prostate cancer in male rats or mice from studies conducted by Syngenta and reviewed by regulatory authorities around the world (AVPMA, 2010; EEC, 1996; USEPA, 2006; 2011; WHO, 2007).

We evaluated the epidemiological evidence for any association between exposure to the chlorotriazines and the incidence of testicular or prostate cancer in men. The weight of the evidence and meta-analysis evaluation were part of a larger review of cancer epidemiology for the chlorotriazines conducted by Breckenridge et al. (2026).

## 2 Organization of the HPA and HPG axes in males

**FIGURE 2:**
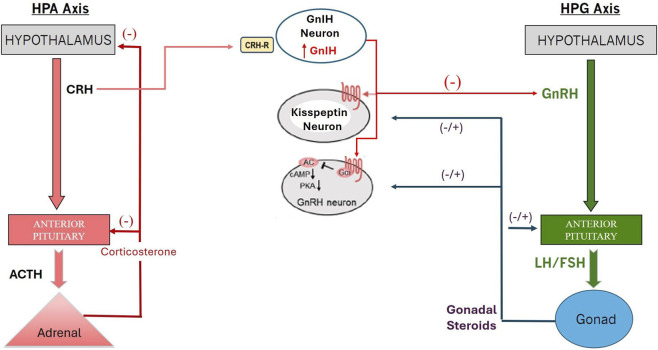
Interaction between the HPA and the HPG Axis via GnlH (Son et al., 2025). (Redrawn in part from Son et al, 2025) Stess-activation of the HPA axis in males increases adrenal gland production of corticosterone via ACTH released from the pituitary gland. CRH concommitantly induces GnIH neurons to release GnIH which dampens kisspeptin neuronal activity leading to a transient suppression of LH and FSH releasedfrom the anterior pituitary, thereby dampening the production of gonadal hormones.

**FIGURE 3:**
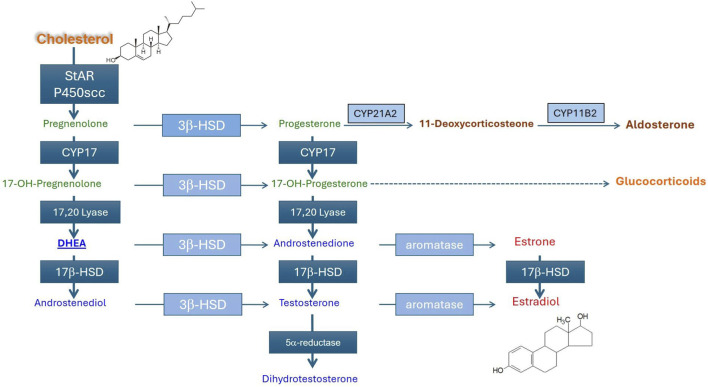
Steroid biosynthesis in the adrenal gland. Luteinizing hormone binds to the LHR, triggering the intracellular release of cAMP. StAR, a protein located in the mitochondria, is activated by cAMP through an intracellular signaling pathway shown in Figure 3. StAR-mediated cholesterol uptake into mitochondria is the rate‐limiting step for steroid biosynthesis. A combination of cytochrome P450 enzymes (CYP11A1, CYP17A1, and CYP19A1) and membrane-bound steroid dehydrogenases (3β-HSD-type 2; HSD17β-type 1) are involved in the synthesis of androgens (androstenedione testosterone) and estrogens (estrone and estradiol).

The HPA axis (Figure 2, left panel) plays an essential role in the regulation of the secretion of adrenal gland corticosteroids and mineralocorticoids (Figure 3). The HPA axis may also play a role in modulating the function of the HPG axis (Figure 2, middle panel).

**FIGURE 4:**
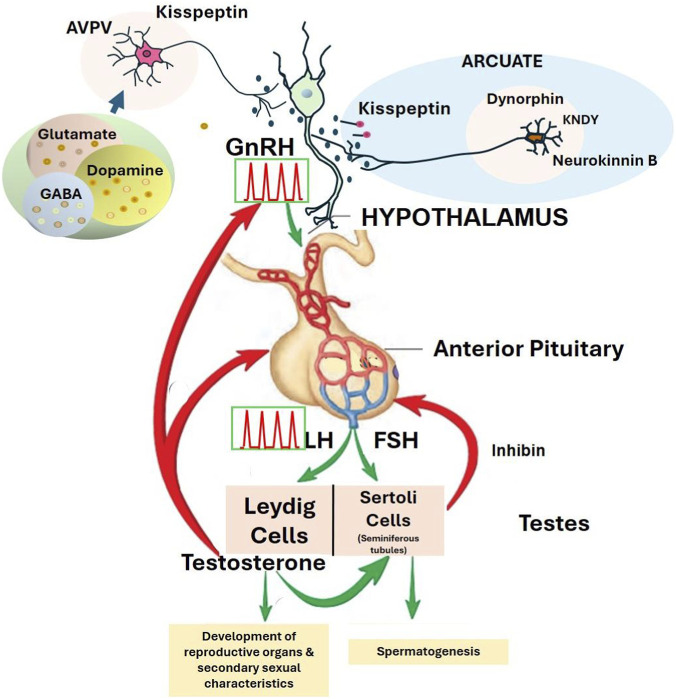
Hypothalamic-pituitary-gonadal axis regulation of the GnRH pulse generator. Figure 4, displays the role of pulsatile release of gonadotropin-releasing hormone (GnRH). the regulation of luteinizing hormone (LH) and follicle-stimulating hormone (FSH) in males. Pulsatile GnRH release is controlled by neuropeptide kisspeptin, which, in turn, is regulated by neurotransmitters and neuropeptides in the hypothalamus arcuate (inhibitory) and anteroventral periventricular nucleus (AVPV) (stimulatory). The activity of Kisspeptin, Neurokinin B, and Dynorphin (KNDy) neurons in the arcuate (ARC) is under the feedback regulation of testosterone, which has a negative feedback influence on the ARC GnRH pulses from GnRH neurons in this region. The AVPV kisspeptinergic apparatus in the AVPV is more prominent in the female and has a primary role in the generation of the ovulatory surge of LH. Unlike females, males are not capable of producing an LH surge, but the pituitary of males does produce LH pulses in response to GnRH pulses. LH stimulates the synthesis and release of testosterone from the Leydig cells in the testicular interstitium. FSH stimulates the Sertoli cells of the seminiferous tubules to increase spermatogenesis and the hormone inhibin. Elevated serum inhibin feeds back on the pituitary to suppress FSH release. Testosterone exerts negative feedback on the hypothalamus and pituitary, resulting in reduced secretion of GnRH, LH, and FSH. Testosterone also has a stimulatory effect on the male reproductive organs, either directly or after conversion to dihydrotestosterone (DHT) by the enzyme 5α-reductase. Within the testes, testosterone binds to androgen receptors in Sertoli cells, which supports the maturation of germ cells into spermatozoa. Red arrows indicate inhibitory pathways. Green arrows indicate stimulatory pathways.

The HPG axis (Figure 2, right panel) is essential for the development and regulation of adult male reproductive functions, including testicular growth, testosterone production, sperm development, secondary sex characteristics, and sexual behavior. A group of neurons in the hypothalamus produces and releases gonadotropin-releasing hormone (GnRH), a decapeptide, into the hypothalamic-hypophyseal portal system, which stimulates the anterior pituitary’s gonadotrophs to release LH and FSH into the bloodstream. Sexually mature males only produce GnRH pulses (Figure 4), while females generate GnRH pulses and also trigger a GnRH surge during proestrus of the ovulatory cycle (Cooper et al., 2025).

**FIGURE 5:**
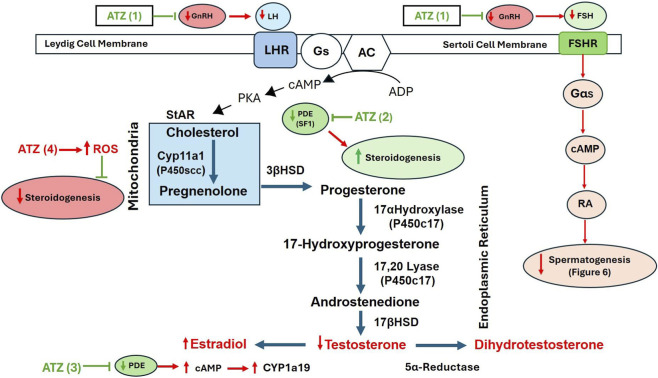
Schematic diagram illustrating the effects of atrazine (ATZ) on hormone pathways in Leydig and Sertoli cells. ATZ impacts GnRH and LH secretion, inhibiting steroidogenesis and leading to decreased testosterone levels. Pathways within mitochondria and the endoplasmic reticulum are detailed, showing interactions with enzymes like Cyp11a1 and 17α-Hydroxylase. ATZ also affects spermatogenesis via FSH pathways in Sertoli cells. Atrazine inhibits GnRH release (ATZ 1) leading to a reduction in LH and FSH secretion. Atrazine inhibits PDE resulting in increased production of estradiol and decreased testosterone by upregulating the expression of aromatase (ATZ 3); SF-1 may play a role in mediating this effect (ATZ 2; Fan et al., (2007b). At high concentrations, atrazine may concurrently increase the production mitochondrial ROS leading to a reducton in mitochondria-mediated steroidogenesis (ATZ4).

LH and FSH, which are released from the pituitary, act on G-protein-coupled receptors on the membranes of Leydig and Sertoli cells in the testes, respectively. Both hormones belong to the same glycoprotein family and share identical alpha subunits, but their different beta subunits account for their functional differences. LH and FSH exert their physiological effects by binding to LHR and FSHR, respectively (Figure 5). When these receptors are activated, they trigger adenylyl cyclase to convert ATP to cAMP, resulting in the activation of PKA-mediated pathways in Sertoli and Leydig cells.

In Leydig cells, LH binds to the luteinizing hormone receptor (LHR) and activates multiple intracellular signaling pathways (Figure 5), thereby increasing mitochondrial cholesterol uptake by elevating StAR protein expression at the mitochondrial membrane. StAR-mediated cholesterol uptake is the rate-limiting step in the steroidogenic pathway. Enhanced cholesterol uptake by mitochondria leads to increased steroidogenesis and testosterone production (see Dufau, 1988; Zirkin and Papadopoulos, 2018; Lei et al., 2025 for reviews).

**FIGURE 6:**
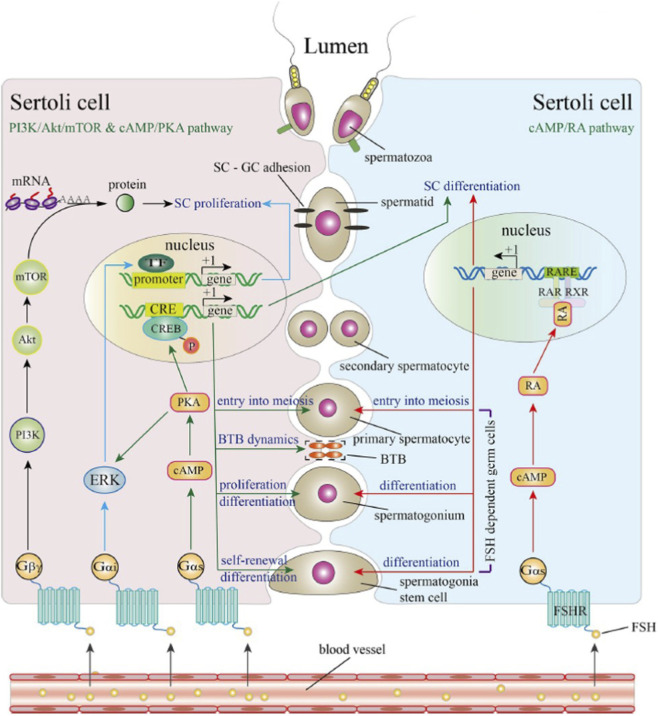
Signaling pathways triggered by FSH in sertoli cells (From Wang et al. (2022)). FSH binds to FSHR on the membrane of Sertoli cells thereby activating FSH signaling. FSHR recruits G proteins to mediate different signaling pathways. Recruitment of Gβγ subunits activates the PI3K/Akt/mTOR signaling pathway, promoting the translation of mRNAs. Recruitment of the Gα subunit activates the cAMP/PKA signaling pathway. Activated PKA directly phosphorylates the CREB protein in the nucleus. Phosphorylated CREB binds to CREs of target genes to regulate transcriptional activity. In addition, PKA activates ERK during Sertoli cell proliferation. ERK activation is also mediated by recruiting the Gαi subunit. FSH may also promote RA biosynthesis through a cAMP-dependent pathway. RA translocates into the nucleus and binds to RAR/RXR to regulate target gene transcription. The cAMP/PKA signaling pathway participates in Sertoli cell differentiation, SSC self-renewal and differentiation, spermatogonia proliferation, and their entry into meiosis, as well as the blood-testes barrier (BTB) dynamics. The cAMP/PKA/ERK signaling pathway and PI3K/Akt/mTOR signaling pathway induce Sertoli cell proliferation. The cAMP/RA signaling pathway has been shown to participate in SSC differentiation, spermatogonia differentiation, and their entry into meiosis.

FSH binds to FSHR on the Sertoli cell membrane, triggering multiple intracellular signaling pathways involved in Sertoli cell proliferation and differentiation. Under the influence of FSH, the mature Sertoli cell regulates and supports spermatogenesis, including the proliferation, differentiation, and meiosis of spermatogonia (Figure 6).

## 3 Effect of atrazine on the HPG axis

### 3.1 Peri-pubertal males

Tables 1–3 summarize the results from studies on the effects of atrazine and its chlorotriazine metabolites on pituitary hormones (LH, FSH, prolactin), gonadal hormone levels (testosterone, estradiol, estrone), and reproductive organ weights in males. These studies evaluated the effect of chlorotriazine during late in gestation (Rosenberg et al., 2008; Stanko et al., 2010; DeSesso et al., 2004) or postnatally (Stoker, et al., 2000; Friedman, 2002; Pogrmic et al., 2009; Trentacoste et al., 2001; Mokhtari et al., 2011; Feyzi-Dehkhargani et al., 2012; DeSesso et al., 2004; Song et al., 2014; Adedara et al., 2021; Khozimy et al., 2022).

**TABLE 1:** Effect of atrazine dosing during gestation on postnatal development, hormone levels and prostate pathology.

Study author/Observation day	ATZ dose (mg/kg/d)	Days of treatment	Absolute body weight and organ weights				Serum concentration				
			Body wt.	Seminal vesicle	Ventral prostate	Testes	LH	Prolactin	Testosterone androstenedione	Estradiol	Estrone
Rayner et al. (2007) (PND 120)	ATZ-ATZ 100	GD 15-19	NE	NE^d^	NE	NE	---	NE	NE	---	NE
Rayner et al. (2007) (PND 220)	ATZ-ATZ 100	GD 15-19	NE	NE^d^	NE	NE	---	NE	NE	---	NE
Stanko et al. (2010) PND 120 and 220	100	GD 15-19	NE	NE	NE	NE	NE	NE	NE	NE	∼40% ↑^e^ ∼90% ↑^f^
DeSesso et al. (2004) ATR 3: (PND 70)^b^	1, 5, 25, 125	GD 6-21	NE	NE	---	NE	---	---	NE	---	---
DeSesso et al. (2004) ATR 3: (PND 170)^c^	1, 5, 25, 125	GD 6-21	NE	NE	---	NE	---	---	NE	---	---

Study author/Observation day	Group ATZ- dose	Day of treatment	PND4 body wt.		Mean (Day of PPS)		Pathology on PND 120			Pathology on PND 220		
			Dams	Pups	Bwt (g)	PPS day	LP incidence^a^	VP incidence	Mylo- peroxidase	LP incidence^a^	VP incidence	Mylo- peroxidase
Rayner et al. (2007): PND 4 Pathology: PND 120 Pathology: PND 220	ATZ-ATZ 100	GD 15-19	13% ↓	6.5% ↓	214.0*	43.7*	1/10 (6/10)	6/10	51.9	4/9	3/9	84.7*
	ATZ-C 100	GD 15-19			229.8	41.6	0/10 (8/10)	8/10	41.3	4/9	2/9	228.7
	C-ATZ 100	GD 15-19	--	--	221.6	42.3	3/10 (7/10)	7/10	45.3	3/10	1/10	155.3
	C-C	GD 15-19	--	--	235.8	41.3	0/10; (8/10)	8/10	44.5	2/8	1/8	224.1

NE, no effect of treatment; * Significantly different from the control group.

PPS, preputial separation; LP, lateral prostate; VP, ventral prostate; Myeloperoxidase is an enzyme secreted by neutrophils, monocytes, and macrophages.

^a^ Two different incidences for LP, inflammation were reported; the incidence in brackets is for the “total incidence of inflammation”. The distinction between them is unclear.

^b^ No effect on spermatid or sperm number at any dose, but the number of abnormally shaped sperm was increased in the 125 mg/kg/day dose group on PND, 70 and PND, 170.

^c^ High dose group (125 mg/kg/day) terminated early due to excessive pre- and post-natal mortality. No animals available for evaluation on PND, 170.

^d^ No effect of treatment on wet or dry seminal vesicle weight.

^e^ PND, 120.

^f^ PND, 180.

**TABLE 2:** Effect of gestational exposure on hormone levels and reproductive organ weights in developing male rats.

Investigator (observation day)	Triazine	Dose (mg/kg/Day)	Duration of treatment	Absolute body and organ weights				Serum concentration					Intratesticular testosterone
				Body weight	Seminal vesicle	Ventral prostate	Epididymis	LH	FSH	Prolactin	Estrone or estradiol	T	
Stanko et al. (2010) (PND 120 and 180)	Atrazine	100	GD 15-19	NE	NE	NE	---	NE	---	NE	NE	↑ 100	---
Rosenberg et al. (2008) (PND 0)	Atrazine	1, 10, 50, 75, 100	GD 15-21	↓ ≥50	---	---	---	---	---	---	---	---	NE
Rosenberg et al. (2008) (PND 60)^a^	Atrazine	1, 10, 50, 75, 100	GD 15-21	NS ↓trend ≥75	NE	NE	Testis: NS ↓ trend ≥50	---	---	---	---	↓ ≥50	↓ ≥75

↓ or ↑ arrows indicate statistically significant increases or decreases in the parameter compared to the control group. The number after the arrow indicates the LOEL, dose for the effect reported.

--- Parameter not examined.

NE, No Effect at the highest dose tested.

^a^ Rosenberg reported a significant, dose-dependent delay in preputial separation at ATZ, doses ≥ 50 mg/kg/day; Anogenital index (adjusted for body wt) was reduced at 100 mg/kg ATZ, group.

**TABLE 3:** Effect of GD 15–19 atrazine or AMM treatment on the prostate of male rats on PND120 and PND180 (Stanko et al., 2010).

Group	Atrazine dose (mg/kg/Day)	PND4 body wt. (% CC)		Mean on day of PPS		Percent of rats with prostate foci		Prostate inflammation on PND 120		Prostate inflammation on PND 180	
		Dams	Pups	Bwt (g)	PPS day	PND 120	PND 180	Incidence^a^	Severity	Incidence^a^	Severity
Control	0	NR	10.4	228	41.5	5%	0%	12/25 (48%)	0.6	11/20 (55%)	0.7
0.09 AMM	0.018	NE	10.9	232	42.4	13%	0%	14/21 (67%)	1.4	4/18 (22%)	0.4
0.087 AMM	0.18	NE	10.9	244*	42.4*	25%	23%	19/25 (76%)	1.4	13/21 (62%)	0.8
8.73 AMM	1.8	NE	10.4	237	42.4*	21%	25%	20/25 (80%)	1.4	11/19 (58%)	0.9
100 ATZ	100	NE	9.8	236	43.1**	23%	50%	17/21 (81%)	1.2	6/16 (34%)	0.7

AMM, Atrazine (25%), DEA (15%), DIA (5%), DACT (35%), and 20% hydroxyatrazine

NR, data not provided.

NE, no effect of treatment; * or **Significantly different from the control group (p < 0.05; or p < 0.01). Statistical analysis appears to be on a pup rather than a litter basis.

PPS, preputial separation.

^a^ Incidence and severity scores are for the lateral and ventral prostate combined.

Rosenberg et al. (2008) examined the dose effects of atrazine exposure during Sprague-Dawley rat gestation on the postnatal development of male offspring. Pregnant dams were treated by oral gavage with atrazine at 0, 1, 10, 50, 75, and 100 mg/kg/d from gestational day (GD) 14 to parturition (Table 2). Atrazine did not affect the number of live births per dam. Neonatal pup survival was reduced with increased pup death seen at doses of 10 mg/kg/d and higher. There was no effect of atrazine on testosterone concentrations within the testes of newborn pups. Anogenital distance, an androgen-dependent process, decreased from the control level at the 75 and 100 mg/kg/d doses, with the decrease reaching significance at the 100 mg/kg/d dose. Preputial separation, also an androgen-dependent process, was significantly delayed at 50 and 100 mg/kg/d compared with controls. On PND60, serum testosterone concentrations were significantly lower than in controls in the 50–100 mg/kg/d groups. However, these decreases had little effect on the weights of the seminal vesicles.

DeSesso et al. (2004) exposed male SD rats to atrazine at doses of 1, 5, 25, or 125 mg/kg/day from GD 6 - 21 and evaluated male offspring on PND 70 or 17 (Table 1). There were no effects of gestational exposure to atrazine on body weight, seminal vesicle, or testis weights on PND 70 or 170. Testosterone levels were comparable to controls, although there was an increase in abnormally shaped sperm in the 125 mg/kg/day dose groups evaluated on PND 70 and 170. In a separate study, DeSesso et al. (2004) found that male SD rats administered a dose of 125 mg/kg/day of atrazine from PND 2-21 had reduced spermatid and sperm numbers on PND 70 and 170. There were no effects on body weight, seminal vesicle, or prostate gland weight, although testicular and epididymis weights were significantly reduced. The NOAEL for these endpoints was 25 mg/kg/day. There was no effect of atrazine treatment on testosterone levels at doses up to 125 mg/kg/day on PND 70 or 170.

Stoker et al. (2000) administered atrazine to male Wistar rats by gavage at doses of 12.5, 25, 50, 150, and 200 mg/kg/day from post-natal day (PND) 23 to PND 53. Lateral prostate weights were significantly decreased at doses of 50 mg/kg/day and above. Intratesticular testosterone and seminal vesicle weights were decreased in the 200 mg/kg/day atrazine-treated group and a group of pair-fed control males. In addition, serum estradiol and estrone were increased in the 200 mg/kg dose and pair-fed males (Table 5).

Trentacoste et al. (2001) administered atrazine by gavage to male Sprague-Dawley rats at doses of 1, 2.5, 5, 10, 25, 50, 100, or 200 mg/kg/day from PND 22 to 47 (Table 5). They found significantly reduced LH, serum testosterone, and intratesticular testosterone at doses of 100 or 200 mg/kg/day. Exposure to high doses of atrazine also reduced ventral prostate and seminal vesicle weights. The NOAEL in this study was 50 mg/kg/day. Trentacoste et al. (2001) also reported that a pair-fed control group, whose food intake was yoked to the 100 mg/kg/day atrazine-treated group, showed effects similar to those in the atrazine-treated group on body weight, ventral prostate, and seminal vesicle weights. In contrast, Friedman (2002) (Table 5) found a significant reduction in both serum and intratesticular testosterone at an atrazine dose of 50 mg/kg/day.

Stoker et al. (2002) administered atrazine, DEA, DIA, or DACT at atrazine equivalent doses of 6.25, 12.5, 25, 50, 100, or 200 mg/kg/day to male Wistar rats from PND 23 to 53 (Table 5). On PND53, mean serum LH tended to be decreased at doses ≥ 50 mg/kg/day for atrazine chlorometabolites, but none of the differences were statistically significant. Pituitary LH levels were significantly reduced in the 200 mg/kg/day DEA-treated group and in the 100 mg/kg/day DIA group, but the clear dose-response was not evident. DACT did not affect serum or pituitary LH levels at doses up to 100 mg/kg/day in this study. This result was unexpected because Minnema et al. (2001) reported equimolar concentrations of atrazine (400 ppm; 25.3 mg/kg/day), simazine (347 ppm; 25.1 mg/kg/day), or DACT (274 ppm; 18.4 mg/kg/day) administered to female SD rats for 6 months suppressed the LH surge. However, as noted by Fraites et al. (2009), atrazine, DEA, and DIA also stimulate the secretion of ACTH, cortisol, and progesterone by activating the adrenal axis. In contrast, DACT had no effect, suggesting that some of atrazine’s effects on LH secretion may be mediated by activation of the hypothalamic-pituitary-adrenal (HPA) axis. The activation of the HPA axis during forced food restriction may account for the results seen in the pair-fed controls (Son et al., 2022).

Overall, the results suggest that high doses of atrazine have an equivocal effect, with decreases in LH, FSH, and serum testosterone, accompanied by decreased body weight and reduced weights of the seminal vesicles, prostate, and epididymis in the majority of studies. Notably, the lack of a compensatory rise in LH and FSH in males treated with doses of atrazine that lower serum testosterone indicates that the central control of the gonadotropins is suppressed. The lowest observed effect level (LOAEL) for a decrease in serum testosterone was 38.5 mg/kg/day. Adedara et al. (2021) reported that 20 mg/kg/day atrazine reduced serum LH, FSH, and testosterone; however, they did not provide any supporting data (Table 5). The weight of the evidence suggests that the NOAEL for the HPG parameters investigated in these studies is 25 mg/kg/day in males.

### 3.2 Studies in adult males

Table 6 summarizes studies that evaluated gonadal hormones and reproductive organs (Victor-Costa et al., 2010; Kniewald et al., 2000) as well as sperm counts and motility (Kniewald et al., 2000; Khozimy et al., 2022) at atrazine doses ranging from 50 to 300 mg/kg/day (10% of the LD_50_). In these high-dose studies, reduced testosterone levels were often associated with reduced LH levels, as reported at lower doses in studies by Trentacoste et al. (2001) and Pogrmic et al. (2009) (Table 6). Likewise, reduced sperm count and motility observed by Kniewald et al. (2000) and Khozimy et al. (2022) were linked to reduced FSH reported by Feyzi-Dehkhargani et al. (2012), Mokhtari et al. (2011), and Adedara et al. (2021) in Table 5. However, Song et al. (2014) reported increased FSH at doses ≥38.5 mg/kg/day and LH at doses ≥77 mg/kg/day.

Table 6 shows that significant reductions in body weight were accompanied by decreased absolute testis and relative testis weight in the study by Khozimy et al. (2022) whereas, Victor-Costa et al. (2010), who tested atrazine at a dose of 300 mg/kg/day, which was 10% of the LD_50_, reported nearly a 40% reduction in body weight accompanied by increased absolute and relative testes weights and a 104% increase in adrenal gland weight after 40 days of treatment. Martin-Santos et al. (2017) and Martin-Santos et al. (2018) associated increased testicular weights with an accumulation of testicular fluid in efferent ductules of the testes with an atrazine-induced disruption of ductal fluid transport.

Jin et al. (2013) reported that a more modest reduction in body weight (15%) at an atrazine dose of 200 mg/kg/day administered to male mice for 3 weeks was accompanied by a 10% increase in relative testis weight. They also found a 34% reduction in testosterone, a 61% increase in aromatase activity, and a 250% increase in estradiol levels. Jin et al. (2013) concluded that it is unlikely that the increase in E2 was responsible for the decrease in testosterone because the serum E2 concentration was approximately 2000-fold lower than serum testosterone levels in male mice.

Foradori et al. (2017) administered atrazine by gavage to eleven-week-old adult male SD rats for 1, 7, 14, or 28 days, after which plasma concentrations of pituitary and adrenal hormones were evaluated. A dose-dependent increase in plasma concentrations of ACTH, corticosterone, and progesterone was seen on day 1, but not after 7, 14, or 28 daily doses (Supplementary Figure S1a, Panel A to C). The NOAEL was 25 mg/kg/day. There were no effects of atrazine on prolactin (Panel D) or androgens (Panel E to G) at any dose for up to 28 days of treatment.

Collectively, these results suggest that atrazine may adversely affect male gonadal hormone levels at doses greater than 25 mg/kg/day, most likely via a direct action on the testes. Based on these results, impaired male fertility would be expected at high doses. However, a two-generation reproduction study conducted on atrazine at “maximum tolerated doses”) of 38.7 mg/kg/day (500 ppm atrazine in diet), did not have any effect on fertility in either the F0 or F1 generations. Testicular weights in the high-dose parental males were comparable to those of controls in both the Fo and F1 generations (DeSesso et al., 2004).

## 4 Effect of atrazine on prolactin regulation in males

**FIGURE 7:**
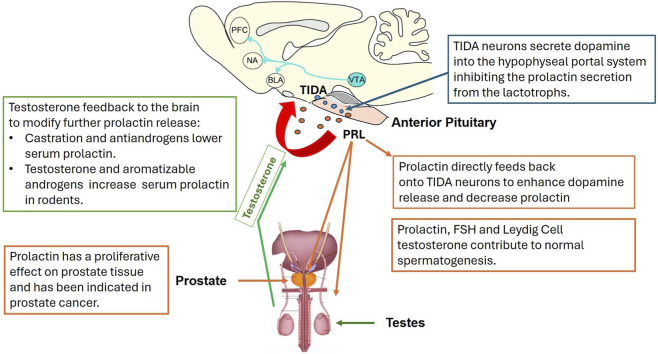
TIDA neuron regulations of prolactin release from the anterior pituitary. TIDA neurons secrete dopamine into the hypophyseal portal system, inhibiting the prolactin secretion from lactotrophs in the pituitary. Prolactin directly feeds back onto TIDA neurons to enhance dopamine release and decrease prolactin secretion. Prolactin, FSH, and Leydig cell testosterone contribute to normal spermatogenesis. Prolactin has a proliferative effect on prostate tissue and has been implicated in prostate cancer. Testosterone feedback to the brain modifies further prolactin release. Castration and antiandrogens lower serum prolactin levels. Testosterone and aromatizable androgens increase serum prolactin in rodents.

Tuberoinfundibular dopaminergic (TIDA) neurons have cell bodies located in the arcuate nucleus of the hypothalamus (Figure 7). These neurons project axons that terminate near the external zone of the median eminence of the hypothalamus. When activated, TIDA neurons release dopamine, which is carried via the hypophysial portal vasculature to the anterior pituitary. Dopamine binds to the dopamine receptor (DRD2), a G-coupled protein receptor on pituitary lactotrophs, leading to the inhibition of prolactin release into the blood (Figure 7; See Qi-Lytle et al., 2024 for a recent review).

Prolactin plays a role in establishing and maintaining the tuberoinfundibular dopaminergic (TIDA) neuron population in rats, particularly during early postnatal life (Lim et al., 2025). Shyr et al. (1986) identified a critical role of milk-derived prolactin in the postnatal development of the TIDA neurons in the adult male rat. The TIDA neurons have a significant inhibitory effect on prolactin secretion from the anterior pituitary. Shyr et al. (1986) dosed lactating dams with bromocriptine on PND 2-5 to block prolactin secretion from the anterior pituitary and demonstrated that this brief deprivation of prolactin had a permanent effect on the development of the TIDA neurons in the median eminence on PND 33-35. This response did not occur if the lactating dams were dosed on PND 9-12. Others had shown that the first postnatal week was essential for TIDA neuronal development (Loizou, 1971; Ojeda and McCann, 1974).

### 4.1 Effect of gestational atrazine dosing on prolactin-mediated inflammation of the prostate

Prolactin influences the prostate as one of its multiple physiological effects in the male (Grattan and Kokay, 2008; Goffin et al., 2011; Costello and Franklin, 2018; Lim et al., 2025). Hypophysectomy reduces pituitary-dependent prostate growth, whereas pituitary graft implantation enhances it. In adult male rats, prolactin can enhance prostate growth, particularly under conditions of hyperprolactinemia or when acting synergistically with androgens. When dopamine release into the pituitary portal system is low, prolactin release is elevated; as serum prolactin concentrations increase, prolactin binds to prolactin receptors on TIDA neurons, enhancing their activity. The increase in dopamine in the pituitary portal suppresses further prolactin release (Qi-Lytle et al., 2024).

Rayner et al. (2007) evaluated the effect of atrazine treatment during gestation (GD 15-19) on the number of days needed to achieve sexual maturation as indicated by preputial separation and on gonadal development (reproductive organ weights; serum testosterone, estrogen, and estradiol), evaluated on postnatal day (PND) 120 and 220 (Table 1). Groups of forty time-pregnant dams were administered either atrazine (ATZ-groups) or the vehicle control (C-group) by gavage from GD 15-19 and then allowed to give birth to their pups. On PND 4, the pups were divided into four subgroups such that atrazine-treated dams fostered either atrazine-treated pups (ATZ-ATZ subgroup) or pups from untreated dams (ATZ-C subgroup). Likewise, control dams cross-fostered either pups from atrazine-treated dams (C-ATZ subgroup) or from untreated control dams (ATZ-C subgroup). Males whose dams were administered 100 mg/kg/day atrazine and cross-fostered by atrazine-treated dams displayed a significant, 2 to 4-day delay in preputial separation. The body weight of atrazine-treated dams decreased significantly in males that received atrazine gestationally. Five sections through the right lateral and the ventral prostate were examined by a pathologist blinded to dose group, and the severity of inflammation was categorized (None, < 1–10%; 25%, 50%, ≥ 75% of the sections affected). The degree of inflammation in the left lateral prostate was also quantified using a myeloperoxidase (MPO) activity. Myeloperoxidase, which is an enzyme secreted by neutrophils, is also found at lower concentrations in monocytes and macrophages.

Preputial separation was delayed in males exposed during gestation and lactation, but the other groups did not differ from controls. For both PND 120 and PND 220 evaluations, there were no consistent or dose-response effects on body weight, testis weight, seminal vesicle weight (wet or dry), and ventral prostate weight. However, these investigators report a significant increase in lateral prostate weight for the gestational and lactational exposed males. At necropsy, this group also exhibited noticeable macroscopic anomalies, including nodular, pale, white foci on the outer surface. These anomalies were not present in the vehicle-vehicle group nor the gestational atrazine-lactation vehicle group.

Comparisons of the measurements of inflammation among the treatment groups revealed little influence of atrazine on this parameter. The incidence of prostate inflammation in postnatally atrazine-exposed animals tended to be greater than in controls; however, this difference was not statistically significant. This conclusion was supported by the more objective measurement of myeloperoxidase scores, which were the same across dose groups on PND 120. Myeloperoxidase scores increased in all groups by PND 220, as expected with increased age. However, the myeloperoxidase values for males exposed postnatally were somewhat lower in rats exposed to atrazine postnatally and significantly lower in rats exposed to atrazine both pre- and postnatally.

Stanko et al. (2010) examined the effect of dosing Long-Evans dams with atrazine at 100 mg/kg (Table 2) or an atrazine metabolite mixture (AMM) at 0.09, 0.87, or 8.73 mg AMM/kg/day (Table 3). The mixture contained atrazine (25%), DEA (15%), DIA (5%), DACT (35%), and 20% hydroxyatrazine. The final administered doses of atrazine were 0.018, 0.8, and 1.8 mg/kg/day. The authors likely selected these doses because the chronic reference dose for atrazine is based on a NOEL of 1.8 mg/kg/day, derived from effects of atrazine on the LH surge in female rats (USEPA, 2011; WHO, 2007). Although these proportions were selected to be representative of exposure to atrazine and its metabolites in drinking water, it is questionable whether hydroxyatrazine should have been included, given that it has a mode of action that does not involve the HPG axis in the same manner as the chlorometabolites of atrazine (Cooper et al., 2026), Also, hydroxyatrazine is not commonly found in drinking water (Breckenridge et al., 2016; Tierney et al., 2008).

Stanko et al. (2010) administered AMM or 100 mg/kg/day atrazine to pregnant dams from gestation days 15 to 19. The male offspring were evaluated during development for selected reproductive endpoints and killed on either PND 120 or on PND 180. Multiple comparisons were conducted for body weight, serum estrogen, estrone, testosterone, LH, prolactin, androgen-dependent tissue weights, and the presence of inflammation in the prostate. Body weight was significantly decreased in the group dosed with the lowest AMM dose and increased in the two higher AMM doses. The body weight of the 100 mg/kg dose group did not differ from that of the control group. There was a slight delay in preputial separation (PPS) with the 0.87 mg and 8.7 mg doses of the metabolite mixture, but no delay was observed in the PPS of the 100 mg/kg/day dose group.

There were no effects of atrazine treatment on serum or pituitary prolactin levels in any AMM or atrazine-treated groups. Serum testosterone results were inconsistent in that there was an increase in testosterone levels in the high-dose atrazine group on PND 120, but not on PND 180. There were no effects on testosterone levels in any AMM-treated group on PND 120. However, a significant decrease was observed in the high-dose AMM group on PND 180. In contrast, the high-dose atrazine-treated group remained unchanged. Results on the effect of atrazine on serum estradiol and estrone were likewise inconclusive (data not shown). Serum estrone was increased on PND 120 and decreased on PND 180 in the 100 mg/kg dose group, but was unaffected in all of the AMM-treated groups on PND 120 or PND 180. Serum estradiol levels were comparable to those in the control group in all AMM and the 100 mg/kg/day atrazine-treated group on PND 120. However, on PND 180, serum estradiol levels were decreased in all AMM and the 100 mg/kg atrazine doses. This latter result was likely due to an unusually high level of estradiol reported for the control group on PND 128 (32.8 ƿg/mL compared to the control level reported on PND 120 (17.8 ƿg/mL).

The prostate pathology report revealed no difference in the incidence of inflammation among the groups on PND 120 or 180. However, a higher percentage of rats showed inflammation on PND 120 (48%–81%) compared to PND 180 (22%–62%). The severity scores of the affected rats showed mild inflammation, with scores ranging from 1.2 to 1.5 on PND 120 and 0.4–0.9 on PND 180 (on a scale of 0–5, with 0 indicating no inflammation). It is challenging to reach any conclusions from this study, as the consistent lack of a dose-response and isolated differences in the numerous endpoints examined may merely reflect the result of multiple comparisons and may have been more a matter of chance rather than due to atrazine treatment.

### 4.2 Effect of post-partum atrazine exposure on TIDA-Regulated prolactin secretion

Stoker et al. (1999) evaluated the effect on prolactin of total daily atrazine doses of 0, 12.5, 25, 50, or 100 mg/kg/day, which were split into doses of 6.25, 12.5, 25, or 50 mg/kg administered twice daily to nursing dams on PND 1-4 (Table 4). A positive control group of nursing dams was administered the dopamine receptor agonist bromocriptine at total daily at doses of 0.052, 0.104, 0.208, or 0.417 mg/kg (split doses, twice daily; data not shown). Serum prolactin was significantly increased in untreated nursing dams within 10 min of the initiation of suckling. Atrazine daily doses of 50 mg/kg/day and 100 mg/kg/day inhibited suckling-induced prolactin release in all females. Atrazine daily doses of 12.5 and 25 mg/kg/day inhibited suckling-induced prolactin release in some dams and had no discernible effect in others. The NOAEL for atrazine on suckling-induced maternal prolactin release during this study was, therefore, a minimum of 12.5 mg/kg/day and as high as 25 mg/kg/day in some females. Bromocriptine inhibited the suckling-induced prolactin release at doses of 0.104–0.417 mg/kg, with no effect at a bromocriptine dose of 0.052 mg/kg. There was no effect of treatment on serum or pituitary prolactin levels on PND 120 in male offspring from dams that had been administered atrazine from PND 1-4.

**TABLE 4:** Effect of atrazine treatment on PND 1-4 on tissue weight and prostate inflammation in wistar rats on PND120 (Stoker et al., 1999).

Atrazine dose^a^ (mg/kg/Day)	Lateral prostate (g)	Ventral prostate (g)	Testis (g) (Mean ± SEM	sPRL (ng/mL) (Mean ± SEM	pPRL (ng/mL) (Mean ± SEM)	Percent of rats with LP inflammation on PND 120	Severity of LP inflammation in affected rats
0	0.099 ± 0.003	0.580 ± 0.021	1.807 ± 0.033	4.440 ± 0.670	2.781 ± 0.126	8.4%	0.11
12.5	0.094 ±0.003	0.675 ± 0.029*	1.779 ± 0.089	2.936 ± 0.777	2.707 ± 0.224	16.2%	---
25	0.108 ± 0.004	0.581 ± 0.029	1.759 ± 0.022	5.367 ± 0.844	3.507 ± 0.247	39.3%*	0.11
50	0.100 ± 0.005	0.592 ± 0.020	1.759 ± 0.022	4.452 ± 1.216	2.850 ± 0.147	42.8%*	0.42*
100	0.101 ± 0.004	0.577 ± 0.024	1.885 ± 0.029	4.020 ± 0.551	2.341 ± 0.180	43.0%*	0.36*
ATZ 0 + oPRL	0.116 ± 0.007*	0.684 ± 0.036*	1.851 ± 0.037	3.524 ± 1.07	2.654 ± 0.125	57.9*	---
ATZ 50 + oPRL	0.108 ± 0.006	0.618 ± 0.031	1.872 ± 0.039	2.862 ± 0.0724	2.839 ± 0.321	9.8%	---
ATZ 100 + oPRL	0.100 ± 0.004	0.561 ± 0.027^b^	1.936 ± 0.035*	0.572 ± 0.109**	2.654 ± 0.185	7.2%	---

^a^ Atrazine was administered twice daily at doses of 6.25, 12.5, 25 or 50 mg/kg/dose occasion resulting in the indicated daily dose of 12.5, 25, 50 and 100 mg/kg/day.

sPRL, serum prolactin level; pPRL, pituitary prolactin level.

* Significantly different from the control group (p < 0.05); ** (p < 0.01.

^b^ Significantly different from the ovine prolactin replacement (oPRL) control group.

Stoker et al. (1999) also evaluated the long-term effects of lactation on the incidence and severity of inflammation in the lateral prostate in the male offspring on PND 90 and PND 120 after treatment of the dams with atrazine from PND 1-4. There was no effect of atrazine treatment on prostate inflammation on PND 90 (data not shown in Table 4). The incidence and severity of prostate inflammation were increased on PND 120 days in the offspring of dams administered atrazine doses of 50 and 100 mg/kg/day (Table 4). The 25 mg/kg atrazine-treated group displayed an increased incidence, but not an increased severity, of prostatitis on PND 120. The NOAEL for atrazine’s effect on prostate inflammation on PND 120 was 12.5 mg/kg/day. Combined treatment of the dams with ovine prolactin and 25 or 50 mg/kg atrazine on PND 1-4 reduced the inflammation observed on PND 120 days in the male offspring, indicating that the increase in inflammation observed after atrazine alone was due to prolactin suppression in the dam. Inflammation was increased in those offspring from dams treated on PND 6-9, but this increase was not significant. Dosing from PND 11-14 was without effect. The critical period for this effect appears to be PND 1-9. However, DeSesso et al. (2004) did not find any effect of atrazine treatment on ventral prostate weights on PND 70 or PND 170 in male offspring exposed to up to 125 mg/kg/day atrazine via their dams from PND 2-21 (Table 4).

Since prolactin plays a role in the development of the prostate (Goffin et al., 2011; Lim et al., 2025) as well as the development of the TIDA neurons (Shyr et al., 1986), the study by Stoker et al. (1999) employing perinatal exposure to atrazine on PND 1-4 establishes a plausible NOAEL of 6.25 mg/kg/day for this effect. Although the reduction in prolactin in the dam was linked to prostatic inflammation in the adult male offspring in the Stoker et al. (1999) study, the effects of atrazine on prostate inflammation following gestational exposure (GD 15-19) at an atrazine dose of 100 mg/kg were equivocal (Stanko et al., 2010; Table 3). In the Stoker et al. (1999) study, similar treatment with atrazine to the nursing dams on lactational days 6–9 was without effect on the prostate. These data support the identification of the sensitive period of exposure identified by Shyr et al. (1986) for a few days immediately after parturition.

In further support of the adverse effects on the prostate reported by Stanko et al. (2010), McBirney et al. (2017) did not identify any adverse effect of atrazine treatment on the prostate of the male offspring from 3 successive generations of males after dosing the FO dams from GD 8-14 with 25 mg/kg.

### 4.3 Effects of atrazine on prolactin secretion in adult males

Foradori et al. (2017) evaluated the effects of atrazine on adrenal hormones and pituitary prolactin in eleven-week-old adult male SD rats for 1, 7, 14, or 28 days, after which plasma concentrations of pituitary and adrenal hormones were evaluated. A dose-dependent increase in plasma concentrations of ACTH, corticosterone, and progesterone was seen on day 1, but not after 7, 14, or 28 daily doses (Supplementary Figure S1, Panels A to C). The NOAEL was 25 mg/kg/day. There were no effects of atrazine on prolactin (Panel D) or testosterone, dihydrotestosterone, or androstenedione (Panel E to G) at any dose for up to 28 days of treatment.

O’Connor et al. (2000) administered 7 mg/kg cyanazine intravenously to 230-g male rats. They monitored serum prolactin levels for 24 h and found a transient increase in prolactin (∼17 ng/mL) for the first 30 min post-infusion. Prolactin levels then returned to baseline for the remainder of the sampling period. In contrast, an infusion of reserpine (2 mg/kg) or haloperidol (1 mg), both of which alter TIDA neural activity, resulted in a marked increase in prolactin during the first hour (40–60 ng/mL) and elevated prolactin (∼20 ng/mL) for up to 10 h thereafter. Based on pharmacokinetic data for atrazine (Simpkins et al., 2026) and standard blood volumes (IUCUC, 2022), we estimated that a single gavage dose of more than 500 mg/kg atrazine would be needed to produce a comparable plasma concentration (120 µg/mL) within 20 min of dosing.

### 4.4 Summary of the effect of atrazine on prolactin secretion in males

Overall, there is no evidence that atrazine increased prolactin in males following gestational (Tables 1–3), early lactational (Tables 4, 5), or peripubertal (Table 5) exposure. Likewise, adult male rats administered atrazine for up to 28 days at doses up to 100 mg/kg/day did not show any effect of treatment on prolactin (Foradori et al., 2017; Supplementary Figure S1a, Panel D). In male rats, testosterone is expected to exert negative feedback on TIDA neurons (Figure 7). Thus, reduced plasma testosterone, generally seen in young adult males (Table 5) and adult rats at high doses of atrazine (Table 6), would be expected to increase plasma prolactin secretion, not decrease it. However, as will be discussed later, the reduction in testosterone in high-dose atrazine-treated males may be mediated by a cytotoxic effect of atrazine on Leydig cells in the testes.

**TABLE 5:** Effect of the chlorotriazines on hormone levels and reproductive organ weights in young adult male rats^a^.

Investigator (observation day)	Triazine	Dose (mg/kg/Day)	Duration of treatment	Absolute body and organ weight				Serum concentration					Intratesticular testosterone
				Body weight	Seminal vesicle	Ventral prostate	Epididymis	LH	FSH	Prolactin	Estrone or E2	T	
Stoker et al. (2000)	Atrazine	12.5, 25, 50, 100, 150, 200	PND 23-53	↓200	↓200	↓ ≥50	---	NE^k^	---	NE	↑ 200	NE	↓200
Stoker et al. (2000)	Atrazine	Pair-fed control	PND 23-53	↓	↓	↓	---	NE^k^	---	NE	↓	NE	NE
Trentacoste et al. (2001)	Atrazine	12.5, 5, 10, 25, 50, 100, 200	PND 22-47	↓ ≥100	↓ ≥100	↓ ≥100	---	↓ 200	---	---	---	↓ ≥100	↓ ≥100
Trentacoste et al. (2001)	Atrazine	Pair-fed control	PND 22-47	↓	↓	↓	---	↓	---	​	---	↓	---
Friedmann (2002)	Atrazine	50 50	PND 22-48 PND 46-48	↓ 50 ↓ 50	--- ---	--- ---	NE	--- ---	--- ---	--- ---	--- ---	↓ 50 ↓ 50	↓ 50 ↓ 50
Stoker et al. (2002)	DIA	12.5, 25, 50, 100, 200	PND 23-53	↓ ≥100	↓ ≥ 50	↓ ≥ 50	↓ ≥100	NE^g^	---	---	NE	↓ ≥ 100	---
	DEA			↓ ≥100	↓ ≥100	↓ ≥100	↓ ≥100	NE^g^ ^,^ ^h^	---	---	NE	NE^j^	---
	DACT			↓ ≥100	↓ ≥100	↓200	↓ ≥100	NE^i^	---	---	↑ ≥ 100	NE^l^	---
DeSesso et al. (2004) ATR 4: (PND 70)^b^	Atrazine	1, 5, 25, 125	PND 2-21	NE	NE	NE	Epid, and testes ↓ 125	---	---	---	---	NE	---
DeSesso et al. (2004) ATR 4: (PND 170)^c^	Atrazine	1, 5, 25, 125	PND 2-21	NE	NE	NE	Epididymis ↓ 125	---	---	---	---	NE	---
Pogrmic et al. (2009)	Atrazine	50, 200	PND 23-51	NE	---	---	---	↓ 200	​	---	​	↓ 200	​
Mokhtari et al. (2011)	Atrazine	100, 200, 400	∼PND 90-104	↓ ≥100	---	---	Testis ↓ ≥100	↓ 200	↓400	---	---	↓ 400	---
Feyzi-Dehkhargani et al. (2012)	Atrazine	100, 200,300	∼ PND 63 for 12, 24, 48 days	NE	---	---	Testis ---	↓ ≥100	↓ ≥100	---	---	↓ ≥100	---
Song et al. (2014) ^d^^,^ ^e^	Atrazine	38.5, 77, 154	PND 35-65	↓77	NE	NE	NE	↑ 77	↑ 38.5	---	---	↓ 38.5	---
Adedara et al. (2021) ^f^	Atrazine	20, 40	PND 77-119	---	---	---	---	↓ 20	↓ 20	---	---	↓ 20	↓ 20

NE, no effect at the highest dose tested; --- parameter not examined.

^a^ ↓ or ↑ arrows indicate statistically significant increases or decreases in the parameter compared to the control group. The number after the arrow indicates the LOEL, dose for the parameter.

^b^ DeSesso et al. (2004) reported no effect on spermatid or sperm number. The percent abnormal sperm was elevated but not significant in the 125 mg/kg/day dose group on PND, 70.

^c^ DeSesso et al. (2004) reported no effect on spermatid or sperm number. The percent abnormal sperm was significantly increased in the 125 mg/kg/day dose group on PND, 170.

^d^ Song et al. (2014) reported a significant reduction in testes weight at the highest doses tested (154 mg/kg/day); the NOAEL, was 77 mg/kg/day.

^e^ Song et al. (2014) reported a significant increase in inhibin-B, at a dose ≥77 mg/kg/day; the NOAEL, was 38.5 mg/kg/day.

^f^ Adedara only showed data for the 40 mg/kg/day dose group, although they state that effects were also seen in the 20 mg/kg/day dose group.

^g^ Mean serum LH, levels tended to be decreased at doses ≥ 50 mg/kg/day but none of the differences were statistically significant.

^h^ Pituitary LH, levels were significantly reduced in the 200 mg/kg DEA, group and in the 100 mg/kg DIA, group; no effect in the 200 mg/kg DACT, group.

^i^ No effect of DACT, on serum or pituitary LH, levels at doses up to 200 mg/kg/day.

^j^ Reduced serum testosterone was evident for DEA, at doses ≥50 mg/kg/day, although not statistically significant.

^k^ LH levels were reduced but were not significantly different from controls.

^l^ Reduced serum testosterone was evident for DACT, at a dose of 200 mg/kg/day although it was not statistically significant.

**TABLE 6:** Effect of atrazine on hormone levels reproductive organ weights in young adult male rats or mice.

Reference	Dose (mg/kg/Day)	No. of days of treatment	Body weight and organ weights				Plasma or serum concentration			Intratesticular	
			Body weight	Relative adrenal wt.	Absolute testis wt.	Relative testis wt.	Testosterone	Estradiol	Estrone	Testosterone	Estradiol
Victor-Costa et al. (2010)	50, 200, 300 After PND 120	7	38% ↓ 300	41% ↑ 300	14% ↑ 300	83% ↑ 300	↓ ≥ 50	NE	---	---	---
		15	35% ↓ 200	64% ↑ 200	16% ↑ 200	80% ↑ 200	↓ ≥ 50	↑ 200	---	↓ 200^a^	↑ 200^a^
		40	35% ↓ 200	104% ↑ 200	60% ↓ 200	41%↓ 200	↓ 200	NE	---	---	---

Reference	Dose (mg/kg/Day	# Days of treatment	Body weight	Epididymis	Ventral prostate	Testis	Sperm in epididymis	Sperm motility	Estradiol	Testosterone	Aromatase
Kniewald et al. (2000)^b^ (IP dosing)	60	2x Weekly (60 days)	∼33% ↓	NE	40% ↓	NE	27% ↓	56% ↓	---	---	---
	120		∼33% ↓	NE	35% ↓	NE	53% ↓	52% ↓	---	---	---
Jin et al. (2013)^c^	50	PND 29-50	∼3% ↓	---	---	NE	---	---	∼47%↑	∼27% ↓	∼23%↑
	100		∼6% ↓	---	---	12% ↑	---	---	225%↑	∼24% ↓	∼62%↑
	200		∼15% ↓	---	---	10% ↑	---	---	250%↑	∼34% ↓	∼61%↑

Reference	Dose (mg/kg/Day)	# Days of treatment	Body weight	Absolute testis wt.	Relative testis wt.	Seminiferous tubules			Distal efferent ductules		
						Normal (%)	Dilated (%)	Atrophic (%)	MCM7	Caspase 3	TUNEL
Martins-Santos et al. (2017), Martins-Santos et al. (2018)	200	7	17% ↓	NE	18%↑	100%	0%	0%	205%↑	NE	NE
		15	22% ↓	NE	47%↑	46.3%	23.2%	30.5%	145%↑	NE	NE
		40	22% ↓	NE	25%↑	46.3%	42.7%	10.8%	192%↑	900%↑	500%↑

Reference	Dose (mg/kg/Day)	# Days of treatment	Body weight (% decrease)	Absolute testis wt.	Relative testis wt.	Testosterone	Sperm count (10^6^/mL)	Sperm motility
Khozimy et al. (2022)^d^	60	30	4.3% ↓	4.7%↓	1%↓	15.4%↓	17.5%↓	7.1%↓
	150	30	7.8% ↓	15%↓	7.6%↓	30.8%↓	21.5%↓	15.6%↓
	300	30	18.3% ↓^e^	29%↓	8.9%↓	61.5%↓	33.0%↓	23.4%↓

Reference	Dose (mg/kg/Day)	# Days of treatment	Body weight (g)	Relative wt. Testis (SD)	Relative wt. Epididymis	Relative wt. Seminal vesicle	Relative wt. Prostate	Sperm motility	# Sperm/g testis (10^6^/g)	# Sperm/g epididymis (10^6^/g)
Abarikwu et al. (2010)	0	7	156.5 ± 21.5	1.14 ± 0.19	0.51 ± 0.07	0.39 ± 0.14	0.21 ± 0.10	83 ± 2.74	79.4 ± 1.30	135.3 ± 5.5
	120	7	141.2 ± 21.79	1.47 ± 0.11**	0.48 ± 0.04	0.44 ± 0.13	0.20 ± 0.11	56 ± 5.48**	51.0 ± 1.00**	106.7 ± 6.3**
200	200	7	124.0 ± 18.1**	1.46 ± 0.23**	0.45 ± 0.03*	0.29 ± 0.15	0.11 ± 0.07*	44 ± 5.37**	48.4 ± 3.60**	98.7 ± 4.8**
	0	16	167.3 ± 25.7	1.15 ± 0.23	0.41 ± 0.03	0.29 ± 0.08	0.10 ± 0.04	85 ± 5.00	56.8 ± 4.80	140.3 ± 6.4
	120	16	155.0 ± 25.82	1.46 ± 0.23*	0.38 ± 0.07	0.31 ± 0.07	0.10 ± 0.04	48 ± 8.36**	43.6 ± 2.00**	92.4 ± 5.2**
	200	16	135.0 ± 21.08**	1.41 ± 0.27*	0.34 ± 0.10*	0.17 ± 0.09**	0.06 ± 0.02*	36 ± 8.94**	42.0 ± 2.30**	73.4 ± 6.3**

NE, no effect of treatment; MCM7: protein marker of cell replication; Caspase 3: Protease enzyme in apoptosis; TUNEL: Biomarker for detecting DNA, fragments in apototic cell death.

The up or down arrows indicate if the parameter was significantly increased (↑) or decreased (↓) compared to the control group; the number after the arrow indicates the LOEL, dose.

* Significantly different from the control group after 7 or 16 days of treatment (p < 0.05) based on Dunnett’s t-test calculated using the means ± SDs (N = 10) reported by Abarikwu et al. (2010).

** Significantly different from the control group after 7 or 16 days of treatment (p < 0.01) based on Dunnett’s t-test calculated using the means ± SDs (N = 10) reported by Abarikwu et al. (2010).

^a^ Only evaluated in the 200 mg/kg group administered atrazine for 15 days.

^b^ 90-day old Fischer-344, rats.

^c^ Male mice received on PND, 21 from the China National Laboratory.

^d^ Male albino rats that were young adult (195 g in weight) upon receipt.

^e^ This group of animals displayed a slight weight loss over the 30 days of treatment.

In contrast to the data from males, Cooper et al. (2000) found that three daily doses of atrazine administered to adult, ovariectomized, estrogen-treated female SD or LE rats significantly reduced prolactin release from the anterior pituitary. This effect was attributed to an inhibition of prolactin secretion by increased dopamine released from TIDA neurons in the median eminence into the hypophysial portal vasculature. The fact that *in vitro* exposure of pituitary lactotrophs to atrazine did not alter prolactin release suggests that the *in vivo* results observed in female rats may have been mediated via TIDA neurons (Cooper et al., 2000). Thus, atrazine had a similar effect on prolactin as reported by Shyr et al. (1986) for bromocriptine, a dopamine receptor agonist.

## 5 Interaction between the HPG and the HPA axes

High doses of atrazine interfere with the function of the GnRH surge and pulse generators in females (Cooper et al., 2025) and likely negatively impact the GnRH pulse generator in males (Figure 2). However, in a systematic review of the published literature on atrazine, we did not find any studies that directly evaluated the effects of atrazine on pulsatile GnRH or LH release in male rats. Toxicity studies conducted in males at atrazine doses ≥ 50 mg/kg/day (Friedmann, 2002; Trentacoste et al., 2001; Pogrmic et al., 2009), and sometimes at doses as high as 400 mg/kg/day (Mokhtari et al., 2011) significantly reduced plasma concentrations of FSH, LH, and testosterone (Table 5). These effects were typically accompanied by reduced body weight and gonadal weights. Thus, high doses of atrazine may directly impact steroid biosynthesis in Leydig cells, thereby reducing circulating testosterone enough to trigger GnRH release in the hypothalamus through decreased testosterone’s negative feedback to the hypothalamus (Pogrmic et al., 2009; Pogrmic-Majik et al., 2010).

### 5.1 Mechanistic studies on the HPA and HPG axes

Most mechanistic studies aimed at identifying the final common pathway underlying the effects of chlorotriazines on the HPA axis have been conducted in female rats. Key results summarized by Cooper et al. (2026) are as follows:

1. Direct infusion of 50 *η*M atrazine, DEA, or DIA into the lateral ventricles of young adult females elicited an ACTH response comparable to that of the positive control, IL-1β; DACT had no effect (Foradori et al., 2018).
2. Neither pituitary cells *ex vivo* (Laws et al., 2009) nor corticotrophs *in vivo* (Foradori et al., 2018) responded to chlorotriazine administration, whereas they rapidly responded to CRH with increased ACTH release.
3. Pre-treatment of female rats with dexamethasone, a synthetic glucocorticoid, suppressed atrazine-induced ACTH release from the pituitary and corticosterone release from the adrenal gland (Foradori et al., 2018).
4. Pre-treatment of female rats with astressin, a CRH receptor antagonist, blocked atrazine-induced HPA activation (Foradori et al., 2018).
5. Unlike stress-induced HPA activation, a single dose of 100 mg/kg atrazine, while increasing plasma corticosterone, did not increase c-Fos immunoreactivity in the periventricular nucleus (Foradori et al., 2018; Supplementary Figure S3, whereas preliminary data suggest that c-Fos expression in the central nucleus of the amygdala was increased (Personal communication; Bob Handa).
6. Zimmerman et al. (2021) reported that atrazine-treated and restraint-stressed ovariectomized females displayed a reduced thickness of the zona glomerulosa in the adrenal gland and reduced expression of aldosterone synthase (CYP11B2) (Figure 3). Somewhat paradoxically, these changes were accompanied by an increase in angiotensin II-induced plasma aldosterone level in 100 mg/kg atrazine-treated female rats (Supplementary Figure S4, Panel A). The basal level of aldosterone was also increased in male rats after a single atrazine dose of 100 mg/kg, but not after 7, 14, or 28 daily doses (Foradori et al., 2017; Supplementary Figure S4, Panel B). Supplementary Figure S8.2 provides a proposed adverse outcome pathway (AOP) for the effect of atrazine on aldosterone. Note in this supplemental figure that we included an AOP subset alteration in dopamine synthesis reported by Das et al. (2003) might play a role in the effect of atrazine on the activation of the HPA axis or the termination of atrazine’s effect after a few days of dosing (Belda and Armanio, 2009). Other neurotransmitters (acetylcholine, noradrenaline, serotonin) may also play a role.

An unpublished 52-week oral toxicity study conducted in beagle dogs at an average daily dose of approximately 34 mg/kg/day in males and females resulted in EKG anomalies, increased heart rate, and myocardial pathology (O’Connor et al., 1987). This dose clearly exceeded the maximum tolerated dose, as evidenced by an approximate 20% reduction in body weight gain and mortality in one of six females. The NOAEL for these effects was 5 mg/kg/day. The effect of high doses of atrazine on the heart does not appear to be unique to dogs. Rajkovic et al. (2014) reported that the administration of atrazine at doses of 50 or 200 mg/kg/day to peripubertal rats induced myocardial angiogenesis and increased mast cell degranulation. Zhao et al. (2024) reported that 4-week-old male C57Bl/6J mice administered atrazine at 50 or 200 mg/kg/day for 4 or 8 weeks resulted in cardiac fibrosis, myofiber damage, apoptosis, and inflammation, which the authors attributed to activation of the NF-kB pathway. These results are consistent with the hypothesis that high doses of atrazine enhance the production of superoxide radicals by mitochondria (See Section 8.5) in cardiomyocytes of atrazine-treated rats (Stolze and Nohl, 1994).

Collectively, these results suggest that atrazine’s effect on the HPA axis likely occurs at the level of the hypothalamus or elsewhere in the CNS (Fraites et al., 2009), but not at the level of the pituitary or adrenal glands. There are numerous potential cellular and molecular candidates for the effects of the chlorotriazines in the hypothalamus or midbrain. For example, Galibiati et al. (2021) suggested that atrazine reduces dopamine synthesis *in vitro* (Das et al., 2003) or *in vivo* (Walters et al., 2015), which could alter prolactin secretion, as discussed in Section 4. Furthermore, interactions between the HPA and HPG axes are likely. These interactions may ultimately be reflected in reductions in the GnRH pulse generator frequency observed by measuring pulsatile LH secretion in atrazine-treated, ovariectomized female rats (Foradori et al., 2009; Supplementary Figure S2a). Interestingly, adrenalectomy blocked the effect of atrazine on LH pulses (Foradori et al., 2009; Supplementary Figure S2b), suggesting that atrazine-induced release of corticosterone or progesterone from the adrenal gland may feed back to the hypothalamus, thereby attenuating the effect of the atrazine-induced increase in CRH and its subsequent effect on the GnRH pulse generator.

### 5.2 Adverse outcome pathways underlying atrazine’s effects on the HPG and HPA axes

#### 5.2.1 HPG axis inhibition

Supplementary Figure S8.1 (Panel A) provided a proposed AOP for the effect of atrazine on the HPG axis. Briefly, it is postulated that atrazine reduces activity in KNDY neurons in the arcuate nucleus (Figure 4), thereby reducing pulsatile GnRH release and, ultimately, LH and FSH release from the pituitary. Consequently, androgen synthesis is reduced in Leydig cells, and spermatogenesis is impaired in Sertoli cells, resulting in decreased fertility and altered sexual and parental behaviors in males.

#### 5.2.2 HPA axis activation

Atrazine, DEA, and DIA, but not DACT, activate the HPA axis through a mechanism not fully understood, leading to increased release of CRH, ACTH, and, ultimately, the glucocorticoid corticosterone (Supplementary Figure S8.2, Top panel). While increased plasma corticosterone has a beneficial anti-inflammatory effect, it also increases testosterone synthesis, which, in turn, may reduce prolactin levels, leading to altered sexual behaviors in males.

During stress, increased prolactin secretion is initiated by increased hypothalamic corticotropin-releasing hormone (CRH) (Pan et al., 1995). However, Kehoe et al. (1992) suggested that the effect of stress on prolactin may not be mediated via TIDA neurons, as there were no changes in TIDA neuronal activity 5 or 30 min after immobilization stress in estrous cycling or lactating females.

Restraint-stressed males displayed c-Fos expression in neurons of the paraventricular nucleus, whereas atrazine-treated males did not (Supplementary Figure S8.3, Top Panel). Since c-Fos is generally considered a marker for neuronal excitation, it would appear that the atrazine activation of the HPA axis does not occur via the pathway triggered by stress.

#### 5.2.3 Interaction between the HPA and HPG axis

Atrazine activation of the HPA axis, as described in Section 5.2.2, may induce a CRH-mediated increase in the release of GnIH, leading to a suppression of GnRH neuronal activity (Figure 2). The AOP for this scenario (Supplementary Figure S8.3, bottom panel) postulates that decreased Leydig cell androgen synthesis reduces negative feedback on TIDA neurons, resulting in increased prolactin secretion and a potential increase in prostatic inflammation.

## 6 Epigenetic studies of atrazine in rodents

Epigenetics is the study of how gene activity is regulated without changing the DNA sequence itself, thereby providing a means by which a wide range of environmental factors can modify the inheritance of acquired traits. In brief, this process unfolds when heritable changes in gene expression that do not involve alterations to the underlying DNA sequence modify how genes are expressed. The molecular basis for such effects involves changes in DNA methylation (to silence genes), histone modifications (to activate or repress genes), chromatin remodeling (to alter DNA accessibility and transcription), and non-coding RNAs that fine-tune gene expression.

Potential epigenetic effects of various pesticides, including atrazine, have been examined. Hao et al. (2016) exposed pregnant outbred CD1 female mice to atrazine (100 mg/kg/day) from E6.5-E15.5, which is the developmental window for heritable epigenetic reprogramming. The F1 generation was bred to obtain F2, and the progeny of F2 were bred to obtain the F3 generation (never directly exposed to atrazine). The majority of their measures focused on the testes. These investigators reported that embryonic exposure to atrazine globally changes tissue-specific RNA expression patterns, which become deregulated concomitant with the alterations in H3K4me3 marks. H3K4me3 refers to the trimethylation of lysine (K) on histone H3, a key epigenetic mark involved in gene regulation. They suggested that the H3K4me3 occupancy pattern in the F3 generation of the atrazine-exposed males is derived from embryonic exposure of F1 males and that the pattern of altered H3K4me3 peaks in F3 is preserved from the previous generations.

McBirney et al. (2017) administered outbred pregnant female rats with an ip dose of 25 mg/kg/day of atrazine from GD 4 to GD 18. The F1 offspring were bred to generate the F2 generation, and the F2 offspring were bred to generate an F3 generation. Males from each generation were killed at 1 year of age, and various pathologies were investigated. The incidence of disease in the testes was not different from controls in the F1 generation (embryos exposed). The greatest increase in the incidence of pathological findings was present in the second generation (germ cells only exposed), whereas the incidence was lower in the F3 generation animals that were never exposed to atrazine. The abnormalities seen in the testes included: azoospermia, atretic seminiferous tubules, vacuoles, sloughed germ cells in the lumen, lack of tubular lumen, increased spermatogonial cell apoptosis, and reduced sperm numbers and motility. In contrast to the differences in testicular disease among the three generations, there was no difference in prostate disease at 1 year of age (examined for vacuoles in glandular epithelium, atrophic ductal epithelium, and hyperplasia of prostatic ducts). This lack of effect at 1 year of age agrees with the results from the regulatory studies on prostate (See Section 8).

The differences between the atrazine vs. control generations have been attributed to differential DNA methylation (McBirney et al., 2017). In a subsequent study, Thorson et al. (2020) further examined the spermatozoa from the same McBirney et al. (2017) study. In this work, the sperm DNA and chromatin were isolated from the F3 males with specific diseases (anomalies) and analyzed for DMRs with methylated DNA immunoprecipitation (MeDIP) sequencing and DHRs with histone chromatin immunoprecipitation (ChIP) sequencing. Transgenerational F3 generation males with or without disease were compared to identify the disease-specific epimutation biomarkers. All pathologies were found to have disease-specific DMRs and DHRs, which were found to predominantly be distinct for each disease. No common DMRs or DHRs were found among all the pathologies. Epi-mutation gene associations were identified and found to correlate with previously known disease-linked genes, an observation that would indicate that these effects may represent potential sperm disease biomarkers for histone retention.

It is difficult to place the epigenetic studies in the context of other studies on the effects of atrazine because the high doses evaluated in the epigenetic studies also have a broad range of neuroendocrine effects on atrazine-treated males, including the activation of the HPG and HPA axes (Meaney et al., 2007). Other researchers have also identified hormonal changes associated with atrazine exposure during the development of F1 offspring which potentially could be linked to epigenetic alterations. Lacking specific information on the changes in the dam’s endocrine environment following atrazine exposure raises the question as to whether the above-noted alterations in the F3 animals were the result of a disruption in the dam’s HPA or HPG hormonal axes or a direct effect of atrazine itself (Zhang and Ho, 2011; Meaney et al., 2007). A straightforward interpretation of these epigenetic studies will require more data from comprehensive dose-response studies.

## 7 Bioassays for prostate and testicular cancer in regulatory studies

### 7.1 Prostatic inflammation

Benign prostatic hyperplasia [BPH] develops spontaneously in dogs and humans (Isaacs, 1983). Winter et al. (1995) induced benign prostatic hyperplasia in dogs by treatment with 17β-estradiol and 5α-dihydrotestosterone. Inflammation can be induced in the lateral prostate of castrated Wistar rats exposed to estradiol, followed by dihydrotestosterone (Tangbanluekal and Robinette, 1993). The presence of inflammation was correlated with increased serum prolactin, elevated pituitary weight, and a greater than 2-fold increase in the lateral prostate DNA concentration. The administration of bromocriptine effectively suppressed the increase in pituitary weight and hyperprolactinemia and mitigated the lateral prostate inflammatory response. Inflammation was restored in the bromocriptine-treated hormone-implanted rats by administering exogenous ovine prolactin. Estradiol-induced inflammation in the rat lateral prostate is mediated at least in part by the release of prolactin from the pituitary. Therefore, hyperprolactinemia in the adult male rat is associated with the development of prostatitis.

### 7.2 Prostate and testicular cancer in animal models

Spontaneously occurring prostate tumors are uncommon in the ventral lobes and rarely occur in the dorsolateral lobes in rats (Bosland, 1987). The Fischer-344, Sprague-Dawley, and Wistar rats have a very low incidence of benign and/or malignant prostate tumors at 24 months of age. Like humans, the incidence of prostate tumors in rats appears to increase with age. Two rat strains, the ACI/segHapBR Rat (Ward et al., 1980) and the germ-free Lobund-Wistar [L-W] (Pollard, 1993; Hoover et al., 1990), appear to develop spontaneous tumors after 24 months of age. The ACI strain is not an ideal model because it forms non-metastasizing adenocarcinomas in the ventral prostate only. The Lobund-Wistar rat appears to be better suited, as it develops metastasizing adenocarcinoma in the dorsolateral prostate in aged males. Spontaneous prostate tumors do not develop in either strain of rat until 30 months. The mouse appears to be relatively refractory to the development of spontaneous prostate tumors. In 46,752 male B6C3F1 mice used in over 300 long-term studies conducted by the National Toxicology Program (NTP), only three tumors (2 adenomas and one carcinoma) of the coagulating gland (dorsocranial or medial prostate) were observed (Mitsumori and Elwell, 1988). Bell et al. (1991) demonstrated that the dog is one domestic species that develops prostate cancer spontaneously. The long latency to tumor development limits the usefulness of the dog as an animal model. Testicular cancer is prevalent in aged Fischer-344 rats (interstitial cell tumors) (Maronpot et al., 2016), and relatively rare in Sprague-Dawley rats.

### 7.3 Sub-chronic, chronic studies and carcinogenicity studies in rodents

Breckenridge et al. (2025) summarized the in-life findings of sub-chronic and chronic studies, as well as carcinogenicity studies, on atrazine in rodents. Despite limitations in using rodent models to evaluate the effects of a chemical on the prostate and testes, we have summarized relevant body weight, organ weight, and microscopic pathology data from regulatory studies on atrazine in Supplementary Tables S1–S5.

Bachmann (1994) conducted a 3-month dietary study of atrazine in rats. Groups of male and female Sprague-Dawley rats were given atrazine in the diet at concentrations of 10, 50, or 500 ppm. Average daily doses were approximately 0.8, 3.3, and 34.1 mg/kg/day in the low, mid-, and high-dose males and females at 13 weeks. Body weight in the high-dose group was reduced by approximately 15% at the end of the study. There were no effects of treatment on testes weights, and no treatment-related effects were observed on the testes or prostate after 13 weeks of treatment (Supplementary Table S1).

Rudzki et al. (1991) conducted a unique 8- and 52-week chronic toxicity study on F2-generation males exposed to atrazine via their dams at 0, 10, 50, or 500 ppm in the diet. The group mean average atrazine doses were 0.8, 4.1, and 37.4 mg/kg/day, and 0.5, 2.3, and 23.6 mg/kg/day after 52 weeks in the 10, 50, and 500 ppm groups, respectively. The testes in males sacrificed after 8 and 52 weeks did not display any treatment-related findings (Supplementary Table S2).

Mayhew (1986) conducted a chronic toxicity/carcinogenicity study on Sprague-Dawley rats. Males received atrazine in the diet for 24 months at concentrations of 0, 10, 70, 500, or 1,000 ppm. The average daily dose was 0.5, 3.5, 25.9, and 53.4 mg/kg/day in the 10, 70, 500, and 1,000 ppm groups, respectively. Significant dose-related reductions in body weight gain were observed in males in the 500 and 1,000 ppm groups throughout the study, with reductions of 8% and 19% at 24 months, respectively (Supplementary Table S3).

Cancers of various types were randomly scattered among the control and treated groups in the epididymis, seminal vesicles, and prostate (Supplementary Table S3). There was a statistically significant increase in the incidence of interstitial cell tumors in the testes of the high-dose group (7/67) compared to the control group (1/65). However, the incidence was within the historical range for control animals in the laboratory. After adjusting for the higher survival in the high-dose group (Supplementary Figure S6), the difference was no longer statistically significant. An increase in the incidence of prostate epithelial hyperplasia was also observed in high-dose males (29/66) and mid-dose males (17/67) compared with vehicle controls (12/65). There were no effects on cancer incidence at doses ≤ 25.9 mg/kg/day in this study.

Thakur (1992) conducted a carcinogenicity study in groups of male Fischer-344 rats that were fed atrazine at 10, 70, 200, or 400 ppm for 24 months. The average daily atrazine dose calculated over 24 months was 0.54, 3.85, 11.04, and 31.87 mg/kg/day, respectively (Supplementary Table S4). The incidence of histopathological findings was comparable between the control and atrazine-treated animals in the testes, epididymis, seminal vesicles, and prostate. Interstitial cell tumors occurred in nearly 100% of the animals, irrespective of treatment. The incidence of interstitial cell hyperplasia was approximately 3%–12% of the animals, again without any evidence of a treatment-dependent effect. Chronic inflammation of the prostate occurred in the majority of animals in all groups. The majority of animals also displayed hypospermia in the epididymis and reduced seminal vesicle secretion.

Hazelette and Green (1987) conducted an oncogenicity study in male CD-1 mice that received atrazine in the diet at concentrations of 10, 300, 1,300, or 3,000 ppm for 18 months. The average daily doses received by these groups of mice over the treatment period were 1.2, 38.4, 194, and 385.7 mg/kg/day. Body weight was reduced approximately 9% in the high-dose group. There were no treatment-related findings and no carcinogenic effect of atrazine in the testes, epididymis, seminal vesicles, or prostate in atrazine-treated CD1 mice (Supplementary Table S5).

Collectively, the studies on atrazine summarized above, including sub-chronic, chronic, and lifetime studies conducted in rats and mice, showed little to no evidence of sustained adverse effects on the male gonadal system at doses up to and including the maximum tolerated dose.

## 8 Effects of atrazine on gonadal steroidogenesis

Figure 5 illustrates the enzymes and intermediates involved in the production of testosterone and dihydrotestosterone by Leydig cells. Steroidogenesis is initiated when cholesterol is transferred from the outer to the inner mitochondrial membrane by steroidogenic acute regulatory protein (StAR; Figure 3). Within the mitochondria, cholesterol is converted to pregnenolone by CYP11A1, the rate-limiting step in the steroidogenic pathway (Papadopoulos, 2012). Two weak androgen intermediates, dehydroepiandrosterone (DHEA) and androstenedione, are first synthesized. The enzyme 17-beta-hydroxysteroid dehydrogenase (17-beta-HSD) converts androstenedione to testosterone. Testosterone acts on the hypothalamus and anterior pituitary via negative feedback to decrease the secretion of LH and FSH.

### 8.1 Steroidogenesis

Testosterone is converted to estradiol-17*β* in target cells that have the enzyme aromatase (CYP19A1). Aromatase is present in many tissues of both males and females, including gonads (Leydig cells, granulosa cells), brain, adipose tissue, placenta, blood vessels, skin, and bone (Simpson and Davis, 2001). Testosterone is also converted to dihydrotestosterone (DHT) by the enzyme 5-alpha-reductase, which is present in the prostate gland, seminal vesicles, epididymis, skin, hair follicles, liver, and brain (Marchetti and Barth, 2013). DHT is significantly more potent than testosterone because it has a 2-fold greater affinity for the androgen receptor and a 5-fold slower rate of dissociation, resulting in a longer half-life than testosterone.

### 8.2 Effect of atrazine on aromatase and other steroidogenic enzymes

Simpkins et al. (2026) presented new data on the effect of atrazine DEA, DIA, and DACT on phosphodiesterase (PDE) expression and activity in H295R cells *in vitro*. They found that atrazine, DEA, and DIA inhibited PDE *in vitro* at concentrations greater than 0.5 µM, whereas DACT, the predominant chlorometabolite (95%) *in vivo*, had no effect. Co-exposure of H295R cells to atrazine and DACT *in vitro* increased atrazine’s IC_50_ for PDE inhibition approximately 10-fold. This result is consistent with that of Roberge et al. (2006), who demonstrated that atrazine did not inhibit PDE4 in hepatocytes that are capable of metabolizing to DACT (Simpkins et al., 2026).

Studies on intact animals suggest that atrazine does not inhibit phosphodiesterase *in vivo* at maximum tolerated doses. Atrazine did not induce uterine weight gain in ovariectomized females in the classical uterotrophic assay (Ashby et al., 2002; Connor et al., 1996; Yamasaki et al., 2000), indicating that endogenous estrogen was not being synthesized in atrazine-treated females. Furthermore, there was no evidence that androgens were being depleted in castrated males in a Hershberger assay (Yamasaki et al., 2004). In fact, the weight of the evidence suggests atrazine may have anti-estrogenic effects in females (Cooper et al., 2026).

The majority of studies on testosterone described earlier show that exposure to atrazine in males led to a decrease in testosterone production and alterations in androgen-dependent organs/tissues. The lack of consistent findings contrasts with changes in serum estradiol and estrogen levels observed in these same studies (Tables 1, 2), suggesting that the conversion of androgens to estrogens *in vivo* is not enhanced by moderate doses of atrazine.

**FIGURE 8:**
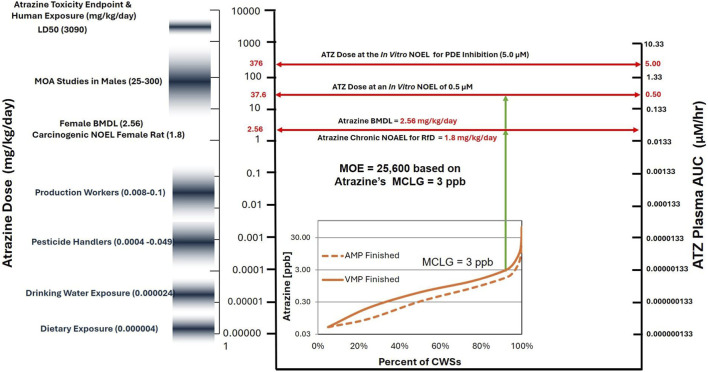
Comparison of NOELS from *in vitro* and *in vivo* studies to human exposure to atrazine (Breckenridge et al., 2016; Baranyai, 1994; Bray et al., 2008; Lunchick and Selman, 1998; Scutaru et al., 2002; Tierney et al., 2008). MCLG = Mean Contaminant Level Guideline (USEPA). AMP Finished = Atrazine Community Water System (CWS) compliance monitoring data collected by States under USEPA’s (1991) Safe Drinking Water Act (SDWA). Data from Tierney et al., 2008; Breckenridge et al., 2016. VMP = Syngenta’s Voluntary Monitoring Program for vulnerable CWSs. AUC = Atrazine’s plasma area under the curve estimate (Data from Experiment 4, Simpkins et al., 2025). Atrazine Benchmark Dose Lower Bound Estimate (BMDL). The BMDL of 2.56 mg/kg/day for atrazine was calculated by USEPA (2011) based upon data collected by Cooper et al. (2010). Atrazine Reference Dose (RfD) = 0.018 mg/kg/day based on a No Observed Effect Level (NOEL) of 1.8 mg/kg/day for the suppression of the LH surge in female rats. An uncertainty factor (UF) of 1000 was used to extrapolate the NOEL to humans. The conversion of μM concentrations of atrazine in vitro to Area under the curve (AUC) concentrations in vivo and the oral gavage dose needed to produce the specific AUC was based upon the following regression equation developed by Simpkins et al., 2025. Y = mX + b, where Y = AUC (μM/L ‐ hr); m = 0.0133 (Slope of linear trendline); X = oral gavage dose (mg/kg/day), b = 0 (Y intercept), The Tier III estimated dietary dose was calculated by Bray et al., 2008 for the US population based upon the reference dose (RfD) for atrazine and its chlorotriazine residues of 0.018 mg/kg/day. Tier III daily dietary exposure to atrazine and its chlorotriazine metabolites was determined to be 0.22% of the RfD. The drinking water dose estimate at the MCLG of 3 ppb (3 μg/L) was calculated for a 60‐kg person drinking 2 liters of water/day. The pesticide handler exposure estimate was based on an evaluation provided by Lunchick and Selman, 1998. They reported the lifetime average daily dose (LADD) for atrazine ranged from 0.008 to 0.1 mg/kg/day depending on the crop treated, method of application, job type, and whether open or closed systems and tractor cabs were used. The estimated daily dose of atrazine for production workers was based on an estimate derived from urine monitoring data from Syngenta’s production facility in St. Gabriel, Louisiana (Baranyai, 1994). The range of doses estimated by Baranyai, 1994 for production workers was comparable to those measured by Scutaru et al. (2002) in the plasma of workers exposed to atrazine in a production facility in Romania.

*In vitro* studies have shown that at concentrations ranging from ∼1 to 20 μM, atrazine inhibits PDE (Roberge et al., 2004), leading to increased cyclic AMP, aromatase expression and activity, resulting in increased rate of conversion of androgens to estrogens (Sanderson et al., 2000; Sanderson et al., 2001; Fan et al., 2007a; Fan et al., 2007 b). However, Simpkins et al. (2026) recently reported that DACT, the major chlorometabolite of atrazine, blocks atrazine’s effect on PDE *in vitro*. Furthermore, pharmacokinetic data on atrazine and its chlorometabolites indicate that the administration of an oral dose of 37.6 mg/kg/day to rats would produce an AUC atrazine plasma concentration of 0.5 μM in rats (Figure 8), which is the NOEL for PDE inhibition *in vitro* (Simpkins et al., 2026).

Therefore, in the absence of the counteractive effect of DACT on PDE inhibition, high doses of atrazine *in vivo* may increase cAMP, leading to increased PKA, which is capable of activating several steroidogenic enzymes, including aromatase (Sanderson et al., 2000; 2001; Fan et al., 2007a; b; Suzawa and Ingraham, 2008). In addition to activating the cAMP-PKA pathway, atrazine has also been shown to influence steroidogenesis *in vitro* by activating the MAPK/ERK pathway (Albanito et al., 2015; Pogrmic-Majkic et al., 2016).

However, others have shown that atrazine inhibits cAMP-PKA-mediated activation of aromatase and estradiol production under normal physiological conditions in steroid-producing cells. Using a primary granulosa cell culture, Fa et al. (2013) examined the effect of atrazine on the stimulatory effect of FSH on these cells. Under normal conditions, FSH activates both the cAMP and extracellular signal-regulated kinase 1/2 (ERK1/2) signaling pathways, with the cAMP pathway being more critical for increasing LH receptor (LHR) and aromatase (CYP19A1) mRNA expression, ultimately leading to increased estradiol production. Fa et al. (2013) demonstrated that atrazine exposure blocked the FSH-stimulated LHR and CYP19A1 mRNA expression and estradiol synthesis, with the LHR mRNA response being more sensitive to atrazine than CYP19A1 and estradiol synthesis. Inadequate acquisition of LHR in FSH-stimulated, atrazine-exposed granulosa cells impairs their standard response to human chorionic gonadotropin (hCG) stimulation. They went on to demonstrate that the atrazine-induced inhibitory response resulted from activation of the ERK1/2 pathway, as inactivation of this pathway by selective inhibitors restored normal functioning.

The complexity of atrazine’s effect on the Leydig cell and granulosa cell is further complicated by the downregulation of the upstream gene SRB1, which is involved in cholesterol uptake, StAR, and other genes involved in supplying mitochondria with the proper levels of cholesterol to maintain proper steroid production (Pogrmic-Majkic et al., 2018). In summary, atrazine has been shown to induce several alterations in the mechanisms involved in the synthesis within the steroidogenic process. Depending on the cell line, it is possible to increase (Sanderson et al., 2000) or inhibit (Jin et al., 2013; Pogrmic-Majkic et al., 2018) estrogen production.

Martins-Santos et al. (2017) evaluated the effect of atrazine on aromatase expression in the efferent ductules of the rete testis. It is known that efferent ductules present the highest levels of estrogen in the luminal fluid and the highest expression of estrogen receptor in the epithelium (Hess et al., 1997b; 2011). Their primary function is to reabsorb fluid coming from the testis, which occurs under the control of estrogens (Hess et al., 2011). Disturbance in fluid reabsorption leads to the accumulation of fluid in the ductule lumen and consequent reflux to the testis, resulting in luminal dilation of the seminiferous tubules followed by testicular atrophy and, consequently, infertility (Hess et al., 1997a; Oliveira et al., 2001; Oliveira et al., 2002).

Martin Santos et al. (2018) administered 200 mg/kg/day administered daily for 7, 15, or 40 days to mature, 100-day-old Wistar rats. They found that atrazine increased luminal diameter, reduced epithelial height, increased aromatase expression, and disrupted the rate of cell proliferation and apoptosis in the efferent ductules (Table 5). In contrast, the effects on the ventral prostate were mild. Given that similar alterations in the efferent ductules lead to dilation and testicular atrophy, they hypothesized that the testicular changes in rats exposed to atrazine may be, at least in part, secondary to the alterations in the efferent ductules that may reflect a disruption in estrogen (ERα) receptor regulation.

### 8.3 *In vitro* studies on Leydig cells

Friedmann (2002) evaluated the effects of atrazine on primary Leydig cell cultures isolated from rats on PND 49 and co-cultured with 232 μM atrazine in the presence of a maximally stimulating concentration of LH. Compared with cells cultured with vehicle and LH alone, testosterone production by Leydig cells treated with atrazine for 72 h was reduced by 35%. A similar decrease was observed when Leydig cells were stimulated with dibutyryl-cAMP (db-cAMP) rather than LH, but not with hydroxycholesterol, indicating that atrazine’s effects on testosterone production can be bypassed. Friedman concluded that these results demonstrate that atrazine directly disrupts testosterone synthesis in the Leydig cell. The results confirm that oral exposure to atrazine lowers circulating and intratesticular levels of testosterone in juvenile rat males and that this disruption of endocrine function is not secondary to weight loss, but rather due to a direct action on Leydig cells.

Karmaus and Zacharewski (2015) exposed mouse BLTK1 Leydig cells to 1–600 µM atrazine either alone or in combination with recombinant human chorionic gonadotropin (rhCG), a luteinizing hormone analog, for 1, 2, 4, 6, 12, or 24 h. They evaluated evidence of phosphodiesterase inhibition (i.e., reduced media concentrations of 5′-AMP and increased cyclic AMP) and effects on steroidogenesis (i.e., increased media concentrations of progesterone and testosterone). They showed that when atrazine was administered in combination with rhCG, the EC_50_ for increased cyclic AMP (cAMP EC_50_ = 111 µM atrazine) was comparable to the EC_50_ for reduced testosterone (Testosterone EC_50_ = 104 µM atrazine) (Supplementary Figure S5). Atrazine, at biologically relevant concentrations *in vitro* (Simpkins et al., 2026; ≤10 μM), had no effect when applied alone or in rhCG-stimulated Leydig cells.

Pogrmic et al. (2009) administered atrazine at oral doses of 0, 50, or 200 mg/kg/day from PND23-51 to male Wistar rats. The serum concentration of testosterone and dihydrotestosterone (DHT) combined was significantly reduced in the high-dose group 24 h after the last dose. LH was lower than in controls, but this difference was not statistically significant (Table 4). The NOEL for the effect of atrazine on LH was 50 mg/kg/day. However, for androgens, a non-significant reduction was evident in the 50 mg/kg/day atrazine-treated group.

Pogrmic et al. (2009) harvested Leydig cells on PND 52 from the control and atrazine-treated groups. They evaluated their testosterone and progesterone production *ex vivo* under basal conditions and after stimulation with hCG or other steroidogenic substrates. In hCG-stimulated cells, they measured extracellular cAMP levels and gene expression related to steroidogenesis. Transcripts for luteinizing hormone receptor (LHR), scavenger receptor B1 (SR-B1), steroidogenic factor-1 (SF-1), steroidogenic acute regulatory protein (StAR), translocator protein (TSPO), phosphodiesterase 4B (PDE4B) which participate in the cAMP-dependent control of steroidogenesis, as well as 3β-hydroxysteroid dehydrogenase (HSD), CYP17A1, and 17β-HSD were determined in hCG-stimulated Leydig cells (Supplementary Table S6b).

Basal serum progesterone and testosterone production were decreased by 200 mg/kg, but only testosterone was decreased at 50 mg/kg/day. The release of progesterone and testosterone was less than that observed in control cells when stimulated with hCG at doses ranging from 0.25 to 20 ng/well. To determine whether substituting the intermediate substrates would restore androgen concentrations to those of the control, these investigators incubated Leydig cells from each atrazine dose group with 22-OH cholesterol, pregnenolone, progesterone, or Δ4-Androstenedione. None of these procedures restored androgen release to control concentrations. Finally, they determined whether atrazine induced changes in gene expression among genes involved in steroidogenesis. They reported that LHR (LH receptor), SR-B1 (primary receptor for high-density lipoproteins), StAR, SF1, PDE4B, CYP17A1, and 17*β*HSD were all downregulated by the 50 and 100 mg/kg/day doses of atrazine. In contrast, TSPO, a transmembrane protein located on the outer mitochondrial membrane, decreased at 200 mg/kg only, and 3*β*HSD was not changed at either dose (Supplementary Table S6b). The results from these studies suggest that atrazine is unlikely to have any effect on Leydig cells *in vivo* at the NOAEL (i.e., 1.8 mg/kg/day) established in the most sensitive animal model (Figure 8).

### 8.4 Effect of atrazine on cell signaling pathways *in vitro*

Albanito et al. (2015) employed gene reporter assays in various cell lines to demonstrate that atrazine does not bind to either estrogen receptor α (ERα) or estrogen receptor β (ERβ). These investigators further reported that atrazine bound to GPER, inducing ERK activation and the expression of estrogen target genes, which appeared to depend on both GPER and ERα expression. Atrazine stimulated the proliferation of ovarian cancer cells that depend on GPER and ERα, as evidenced by gene silencing and specific signaling inhibitors. Notably, through GPER, atrazine induced ERK phosphorylation, gene expression, and migration in cancer-associated fibroblasts (CAFs), suggesting that atrazine stimulates tumor growth by enhancing the tumor microenvironment (Supplementary Table S6c).

Hu et al. (2016) evaluated the *in vitro* effect of atrazine on a mouse prostate cancer cell line (RM1). They reported increased cell viability based on MTT and increased cell proliferation based on MPP (a measure of mitochondrial membrane potential that can be indicative of apoptosis), and increased optical density of cells in culture following exposure to atrazine for 48 h at concentrations of 0.01 or 0.1 µM (Supplementary Table S6a). There was no effect of atrazine on the proliferation of human prostate cancers (LNCaP) at atrazine concentrations up to 30 μM, either with or without DHT added to the media (Robitaille et al., 2015; Supplementary Table S6a). Atrazine has been reported to decrease cell viability and increase apoptosis in several cell types evaluated *in vitro* (Supplementary Table S6c). However, Surapinit et al. (2024) reported a biphasic response, with increased mitochondrial membrane potential for apoptosis (MPP) at low concentrations and decreased MPP at high concentrations (Supplementary Table S6c).

Hu et al. (2016) also evaluated the effect of atrazine on the growth of tumor xenografts of RM1 cells, which were injected subcutaneously into the flanks of recipient C57BL/6 mice. The mice were then administered atrazine (20 or 100 mg/kg/day) for 15 days. They reported a dose-dependent increase in tumor weight at sacrifice on day 15. It is well established that high doses of atrazine activate the HPA axis in rats (Fraites et al., 2009; Laws et al., 2009), resulting in elevated plasma corticosterone levels. Pruett et al. (2009) showed that atrazine doses of 50 and 100 mg/kg reduced B lymphocytes in the blood of B6C3F1 mice. Thus, the high-dose, though short-lasting, effects of atrazine on the HPA axis may have altered RM1 tumor growth reported by Hu et al. (2016).

Chan et al. (2010) also evaluated the effect of GPER on the growth of prostate cancer cells *in vitro* and the growth of prostate xenografts *in vivo*. In contrast to the results reported by Hu et al. (2016), Chan et al. (2010) found that activation of GPER (also known as GPR30) with a selective ligand (G1) inhibited the growth of androgen-dependent prostate cancer cells (PCa) *in vitro* and PC-3 xenografts *in vivo*. G1 elicited no growth or histological changes in the prostates of intact mice and did not inhibit the growth of benign prostatic epithelial cancer cells (BPH-1). Treatment of PC-3 cells with G-1 induced cell-cycle arrest at the G2 phase and reduced the expression of G2-checkpoint regulators.

The inconsistency in the results of studies identifying GPER as a mediator of atrazine effects is not unique. Cancer cell lines differ from normal cells in growth behavior, genetic stability, and response to signals. Although using specific cell lines are excellent for highly targeted research, they are not necessarily good models *in vivo* biology (Ertel et al., 2006) Perhaps the most prominent difference is cancer cells are often insensitive or hyper-responsive due to altered signaling pathways, which may be the fundamental difference between the Hu et al. (2016) study and the Chan et al. (2010).

In summary

1. Normal cells are controlled by contact inhibition and cell-cycle checkpoints, whereas cancer cells typically show greater proliferation.
2. In normal cells apoptosis may be triggered by DNA damage, whereas cancer cells often evade apoptosis;
3. Primary cells *in vitro* have a relatively stable genome, while cancer cell lines display a greater number of mutations and chromosomal aberrations.
4. Normal cells maintain tissue-specific functions, which may be impaired in cancer cells;
5. Normal cells that rely on oxidative phosphorylation for energy production, whereas there are fundamental differences in normal cells and cancer cells that favor glycolysis and the production of lactate (i.e., the Warburg effect) even when oxygen is available,
6. Normal cells typically display a lower rate of cell division, whereas cancer cells bypass senescence mechanisms.
7. Primary cells are non-invasive *in vitro*, whereas cancer cells exhibit invasive or metastatic traits.

A commentary by Chevalier et al. (2016a) noted a similar discrepancy in their work and that of Albanito et al. (2015). Chevalier et al. (2016a) examined the effect of atrazine on the JKT-1 cell line (derived from a human testicular seminoma), which also expresses ERα and ER*β*. In contrast to the results reported by Albanito et al. (2015), who found enhanced cell proliferation, Chevalier et al. (2016a) reported that atrazine suppressed JKT-1 cell proliferation at concentrations ranging from 1 μM to 1 mM. These data illustrate that “the same chemical compound (atrazine) can exert opposite effects in different cancer cells (seminoma, ovarian, or breast) through the same GPR30 receptor” (Chevalier et al., 2016a; b). It also highlights the difficulty of predicting *in vivo* effects from non-physiological *in vitro* data.

In addition to the potential difficulties in interpreting *in vitro* data on atrazine’s potential carcinogenicity, there are other questions about the relevance of these studies to observations made in intact animals. Fa et al. (2013) conducted a study to identify the signal transduction pathways involved in atrazine’s effects on steroidogenesis, using primary granulosa cell cultures. They report two outcomes relevant to predicting the stimulatory versus inhibitory effects of atrazine on estrogen production. Fa et al. (2013) reported that, in primary granulosa cell culture, atrazine at lower doses increased aromatase and estrogen synthesis, presumably via activation of the cAMP-PKA and ERK1 pathways. However, when the dose was increased in the same experiment, the increase in aromatase and estrogen concentrations was blocked. They concluded that the inverted U-shape dose-response for atrazine resulted from activating the known stimulatory pathways (PKA and ERK1) at low doses. However, at higher concentrations, an inhibitory transcription factor was found to suppress hormone synthesis. These results may partially explain why *in vivo* dosing decreases estrogen-dependent responses, such as the delay in vaginal opening and smaller uterine weights observed in female pubertal studies (Ashby et al., 2002; Laws et al., 2003).

The effect of atrazine on steroid hormones is not only dose-dependent, but also dependent on the number of days of dosing. Several investigators have demonstrated a significant, dose-dependent increase in pituitary, gonadal, and adrenal hormones after a single dose of atrazine or its metabolites, as evidenced by increased release of LH, ACTH, and gonadal and adrenal steroid hormones, which lasts approximately 3–6 h. Notably, the amplitude of the response observed for LH (Goldman et al., 2013), ACTH (Fraites et al., 2009; Laws et al., 2009), corticosterone (Fraites et al., 2009; Laws et al., 2009), and testosterone (Riffle et al., 2014) observed after the first atrazine exposure (day 1), but the amplitude of the response returns to baseline or lower if the treatment continued for 3–7 days. Fraites et al. (2009) demonstrated that this response was not related to stress associated with the gavage procedure, gastrointestinal disturbance, or general stress of the procedure. Similar results have been reported by Pogrmic et al. (2009) and Pogrmic-Majkic et al. (2010) for the activation and inhibition of steroidogenesis in peripubertal rats using a combination of *in vivo* and *in vitro* exposures. The importance of these observations underscores the limitations of extrapolating the results from short-duration, high-concentration *in vitro* experiments (e.g., Sanderson et al., 2000; 2001; Fan et al., 2007a; b; Suzawa and Ingraham, 2008; Karmaus and Zacharewski, 2015) or acute *in vivo* exposure (discussed above) (∼1–3 days) to effects observed following longer duration *in vivo* studies.

### 8.5 Reactive oxygen species (ROS)

Oxidative stress occurs when the antioxidant defense mechanisms are overwhelmed by reactive oxygen species (ROS) generated during exposure to a toxicant. This imbalance (with excess ROS) can modify proteins and lipids, alter DNA structure, activate stress-induced transcription factors, and induce the production of inflammatory cytokines. General pathways by which pesticides increase ROS levels include oxidative metabolism by cytochrome P450 enzymes, the generation of redox-active metabolites, and the impairment of mitochondrial electron transport cascades. ROS react readily with lipids, proteins, carbohydrates, and nucleic acids, and in doing so affect the structure and function of cellular components. When free radicals attack unsaturated fatty acids in cell membranes, lipid peroxidation occurs. One of the most common markers of lipid peroxidation is malondialdehyde (MDA), which is formed as a product of this process. MDA is a known mutagen. Radical chain reaction of lipid peroxidation appears to be a continuous physiological process. This process, if left unchecked, alters essential cell functions and leads to cell death. Antioxidant defense mechanisms include the production of superoxide dismutase (SOD), catalase (CAT), and glutathione peroxidase (GPx), as well as non-enzymatic systems such as glutathione. SOD is considered the first defense against oxidative stress, as it catalyzes the dismutation of two superoxide radicals (O_2_-) into molecular oxygen (O_2_) and hydrogen peroxide (H_2_O_2_), which is then neutralized by the combined action of CAT and GPx.

Several studies have reported that high doses of atrazine promote oxidative stress by increasing the concentration of reactive oxygen species (ROS) and the products of oxidative damage, such as lipid peroxides, and therefore influencing the activity of antioxidant enzymes (AOE) (Abarikwu et al., 2010; Adesiyan et al., 2011; Singh et al., 2011; Jin et al., 2010). The liver is the primary target of ingested oxidants and a vital tissue in defense against oxidative stress. However, when induced, liver CYP xenobiotic metabolizing isozymes can also become sources of ROS (Twaroski et al., 2001). The testes also possess antioxidant defense, and changes in the activity of antioxidant enzymes have been recorded in xenobiotic-exposed animals (Andric et al., 2003). Supplementary Figure S8.6, adapted from Abarikwu et al. (2023) and Galbiati et al. (2021), presents a cartoon depicting the effects of atrazine on various signalling pathways that may be involved in atrazine-induced ROS in cells and mitochondria. Supplementary Figure S8.7 represents this as an adverse outcome pathway.

Pogrmic-Majkic et al. (2012) examined the relationship between the effect of atrazine on serum androgens (testosterone plus DHT) and the increase in the activity of antioxidant enzymes, SOT, CAT, and GPx in the primary Leydig cell cultures obtained from peripubertal rats dosed with atrazine (50 mg/mL or 200 mg/mL) from PND 23 to PND 50. They report that the activity of all enzymes, except SOD, decreased in animals exposed to atrazine. The most substantial dose-dependent inhibitory effect of atrazine was observed on GST activity, with enzyme activity 1.4 times lower in the 50 mg/kg group and 2.5 times lower in the 200 mg/kg group compared to the control. The activities of CAT and GSH-Px were inhibited only by the 200 mg/kg dose of atrazine, with the activities decreased to 80% compared to the control. The opposite effect of atrazine, which resulted in enhanced activity of GSH-Px and GST in comparison to the control, was detected in liver AOE. The activity of GSH-Px exceeded the control values by 1.2 times in the 50 mg/kg experimental group and by 1.3 times in the 200 mg/kg experimental group, while GST activity was significantly increased by atrazine exposure only.

Testicular androgenesis was assessed *ex vivo* in Leydig cells from the same animals used for the AOE measurements described earlier. The results indicated that basal androgen production was significantly lower in both the 50 and 200 mg/kg/day atrazine-treated groups. Additionally, 10 rhCG (10 ng/mL) stimulation of androgen production in these groups was also significantly diminished. Since atrazine inhibited several antioxidant enzymes (GST, GSH-Px, and CAT) in the interstitial compartment of peripubertal rat testes, Pogrmic-Majkic et al. (2012) concluded that reduced antioxidant defense is likely related to increased oxidative stress. The elevated oxidative stress observed in these cells would promote a prooxidant state, leading to free-radical-mediated inhibition of steroidogenesis.

The work of Pogrmic-Majkic et al. (2012) and Fa et al. (2013), demonstrating that atrazine exposure reduces antioxidant defense, is consistent with studies suggesting that atrazine impairs mitochondria, reduces mitochondrial membrane potential, and inhibits ATP production (Lim et al., 2025). Adverse effects on mitochondrial function have been linked to dysfunction of electron transport chain complexes, particularly complexes I and III. (Karadayian et al., 2022). Combined, these observations suggest that the mitochondria are a primary target in the cells for the initiation of ROS and increased oxidative damage to the mitochondria, and impair steroidogenesis by disrupting the activity of StAR (transferring cholesterol across the mitochondrial membranes) and the first enzymatic step converting cholesterol to pregnenolone by cytochrome P450scc (CYP11A1) occurs on the inner mitochondrial membrane.

However, the interaction between ROS production and testosterone concentrations may be more complex. The above discussion would suggest that the temporal pattern and dose-response data presented by Pogrmic-Majkic et al. (2012) suggest that within the Leydig cell, high concentrations of atrazine create a mitochondrial redox imbalance as shown in Supplementary Figure S8.7, leading to cytotoxicity, impaired steroid biosynthesis by Leydig cells, and disrupted spermatogenesis in Sertoli cells. This pathway implies that the atrazine dose that effectively alters mitochondrial function would also alter steroidogenesis, AEO, and ROS production, as all depend on normal mitochondrial function. However, the AOP pathway shown in Supplementary Figure S8.7 does not include other pathways that could also lead to altered steroidogenesis and impaired spermatogenesis. Figure 5 illustrates the effect of atrazine phosphodiesterase (PDE4) and associated changes in cAMP activity reported by Fa et al. (2013), Sanderson et al. (2000), and Sanderson et al. (2001) as represented by the AOP shown in Supplementary Figure S8.5 or targeted changes in SF1 reported by Fan et al., 2007a, b; Suzawa and Ingraham, 2008) discussed above also represented in Figure 5.

Leydig cells are a target for their own steroid product, testosterone, and thus could be subject to short-loop feedback regulation by androgens. This effect of testosterone is mediated by the androgen receptor (AR), whose level of expression is in turn regulated by the tissue concentrations of androgens. Hwang et al. (2011) using TM3 Leydig cells showed that testosterone supplementation had cytoprotective effects at 100 nmol/L, but cytotoxic effects at ≥ 500 nmol/L. Significantly reduced ROS generation, lipid peroxide contents, and hypoxia induction factor (HIF)-1α stabilization and activation were present after 100-nmol/L testosterone treatment. They further noted that increased StAR activity was observed at lower doses of testosterone (50 nmol L^-1^), but increasing testosterone to 500 nmol L^-1^ actually increased ROS concentrations. In this *in vitro* study, testosterone supplementation likely modulated intracellular testosterone production via the ROS signaling pathway or a non-genomic signaling pathway.

Tam et al. (2003) showed that CAT, GST, and SOD are decreased in the prostate of castrated male rats, and that control levels of AEO can be restored if the male is administered testosterone propionate. This suggests that changes in testosterone availability alone may drive changes in ROS and/or antioxidant enzymes. This hypothesis was examined by Mgbudom-Okah et al. (2023) and Mgbudom-Okah et al. (2024) in a study of male rats that were treated with atrazine (50 mg/kg for 6 days a week) or atrazine plus testosterone (i.p. (200, 400, or 800 µg/kg) every other day for 52 days. Compared to the atrazine-only group, sperm quality was increased, and the number of abnormal sperm was decreased in atrazine plus testosterone co-treated rats. Lipid peroxidation decreased, whereas lactate dehydrogenase activity and testosterone concentration increased after testosterone co-treatment. Testosterone co-treatment was reported to increase the epithelial thickness and diameter of the seminiferous tubules in a dose-dependent manner, and to dose-dependently elevate glutathione concentration and the enzyme activities of superoxide dismutase and catalase in the testes. The data suggest that testosterone replacement mitigated the depletion of testosterone and minimized the atrazine-induced diminution of defensive inflammatory molecules and antioxidant systems, further elucidating the relevance of androgens under inflammatory and oxidative stress conditions in the testes.

As evidence of testosterone’s role in the reduction of ROS production, the AR antagonist, flutamide, causes elevated levels of ROS and impaired mitochondrial function as indicated by loss in MMP and ATP depletion in cultured murine hepatocytes (HepG2 cell line) (Zhang et al., 2018). Similarly, in the acinar epithelium of the rat prostate, castration yields an increase in ROS-generating NAD(P)H oxidases such as Nox1 [NAD(P)H oxidase 1], GP91PHOX (catalytic subunit of Nox2), and Nox4 (Tam et al., 2003). Furthermore, reductions in the levels of some antioxidant enzymes involved in ROS detoxification, such as superoxide dismutase 2 (SOD2), glutathione peroxidase 1 (GPX1), thioredoxin, and peroxiredoxin 5, occur in the prostate of castrated rats. In males, functional AR and normal androgen levels appear critical for maintaining normal ROS production.

In summary, high doses of atrazine lead to testicular inflammation by a multistep process shown below:

1. Mitochondrial-mediated increase in TNFα and NO resulting in the overproduction of ROS
2. Increased binding of NF-κB by IKK in the cytoplasm
3. Increased translocation of NF-κB to the nucleus
4. Increased cytokine production leading to inflammation.

At the same time, atrazine reduces testosterone synthesis by downregulating StAR and other enzymes involved in steroid biosynthesis. This results in decreased spermatogenesis.

Mgbudom-Okah et al. (2023) and Mgbudom-Okah et al. (2024) reported that when rats were co-administered testosterone and atrazine, the inflammatory response was ameliorated because, in the presence of adequate levels of testosterone, ROS production by mitochondria was suppressed, and the signaling pathways involved mediating inflammation and other adverse effects of atrazine on the testes were not triggered. Likewise, the co-administration of atrazine and antioxidant biflavanoids (Abarikwu et al., 2011; 2012a; b; Ndufeiya-Kumasi et al., 2022) reduced ROS production observed in animals treated with high doses of atrazine alone. Likewise, manganese, a trace element found in mitochondria as manganese superoxide dismutase (MnSOD), a mitochondrial enzyme that scavenges free radicals, protected the liver and kidney from atrazine-induced oxidative stress in Wistar rats (Owumi et al., 2025).

Although the above discussion provides a framework for the pathways mediating the impact of atrazine on both steroidogenesis and inflammation, the doses employed in these studies have been relatively large (e.g., often above the MTD based on body weight) *in vivo* or above the limit of solubility (33 mg/L of atrazine is approximately 153 µM). Furthermore, the suggestion that atrazine directly induces ROS at low doses is inconsistent with the observation that hydrogen peroxide (H_2_O_2_) levels were unaffected *in vitro* in four different cell lines (H295R, JEG-3, H-22 and MCF-7) exposed to atrazine for up to 72 h at concentrations of 10, 30, 100 or 300 µM (Supplementary Figure S7; Simpkins et al., 2026). However, Abarikwu et al. (2013) reported that exposure of primary cultures of rat Leydig cells to atrazine concentrations of 46.4 or 116 µM resulted in increased lipid peroxidation (MDA) and glutathione moieties and reduced SOD and GST levels (Supplementary Table S6a). The NOAEL was 23.2 µM.

More recently, Chen et al. (2016) demonstrated that testosterone deficiency increased the expression of TNF-α and nitric oxide (NO) secretion in response to lipopolysaccharides (LPS) in rat splenocytes. Testosterone treatment of orchiectomized animals also significantly attenuated LPS-elicited TNF-α and inducible nitric oxide (iNO) release in a dose-dependent manner.

Related to atrazine effects on the male reproductive organs, several studies report detailed atrazine-induced increases in ROS or decreases in antioxidant defence in a variety of other tissues. These studies were recently reviewed by Abarikwu et al. (2023). These authors identified several molecular changes associated with atrazine-induced redox imbalance, cytotoxicity, and apoptosis (See Supplementary Figures S8.5, 8.6).

## 9 Summary: atrazine’s effects on male reproductive organs

The studies reviewed above demonstrate that atrazine and atrazine metabolites, when administered at high doses, will disrupt testosterone synthesis, impair testicular function, and alter androgen-dependent tissues. As shown in Tables 1–6, such adverse changes in the reproductive tract of the prepubertal or adult male are absent at lower doses. This conclusion is supported by the results from several sub-chronic and chronic carcinogenicity studies in rodents (Supplementary Tables S1–S5). These studies were conducted using dietary exposure to atrazine at a wide range of concentrations. The oral dose of atrazine required to induce a change in androgen-dependent organ weights was typically greater than 25 mg/kg/day.

Additionally, these results demonstrate minimal effects of atrazine on the histopathology of testes, seminiferous tubules, epididymides, or prostates in male rats and mice exposed for 8, 13, 52, or 106 weeks. It is noteworthy that cancer in any of these androgen-dependent organs is relatively rare in most rat strains except for the Fischer-344 rat. In particular, the effects of atrazine on prostate inflammation were absent in all doses in the Sprague-Dawley males, regardless of the duration of exposure. The observation that both control and all treated Fischer 344 male rat exposed to atrazine is in agreement with the well-documented incidence of spontaneous testicular tumors and accompanying prostatitis in this strain (Derelanko and Auletta, 2014), The lack of effect in the males of other strains exposed long-term to atrazine is in contrast with the results reported by Stoker et al. (1999) in which prostate hyperplasia and inflammation was observed in response to treatment of the lactating dam to atrazine. However, it is worth noting that Stoker et al. (1999) exposed the dam to atrazine. This deprived the male offspring of maternal prolactin during development. However, histopathological effects on the prostate in the male rat dosed directly with atrazine were generally absent, even at high doses (e.g., Martins-Santos et al., 2018). Martins-Santos et al. (2018) also examined the effect of high-dose atrazine on testicular, efferent ducts, and prostate aromatase. They reported that aromatase was elevated in the testis and efferent ducts; however, there was no change in prostate aromatase.

A recent review by Abarikwu et al. (2023) highlighted studies indicating that atrazine promotes oxidative stress by increasing reactive oxygen species (ROS) levels and decreasing the enzymes that remove them, both *in vivo* and *in vitro*. It is noteworthy that Abarikwu et al. (2023) concluded that the dose of atrazine required to increase ROS, impair mitochondrial function, and induce cytotoxicity across different model systems is unlikely to be found in the environment. Thus, the environmental levels of atrazine may not exceed levels of concern for humans, as shown in Figure 8. A similar conclusion can be drawn for the relevance of the studies on prostate and testicular dysgenesis identified in the studies by Victor-Costa (2010) and Martins-Santos et al. (2018), in which 200 mg/kg atrazine altered the reabsorption of luminal fluid in efferent ducts of the testes, including luminal dilation, reduced epithelial height, altered steroidogenesis, and spermatogenesis. In studies conducted at lower atrazine doses, no effects on testicular histology (Supplementary Tables S1–S5) or intratesticular testosterone levels (Stoker et al., 2000) were observed.

Increased aromatase activity as a result of PDE inhibition *in vitro* has raised the concern that increased aromatase activity, which is essential for the conversion of androgens to estrogens, may drive a higher incidence of breast and prostate cancer (Sanderson et al., 2001; 2002; Fan et al., 2007a; b). Simpkins et al. (2026) have clearly shown, however, that DACT, the major metabolite of atrazine, blocks the effect of atrazine on phosphodiesterase. Furthermore, *in vivo* data suggest that atrazine has both antiestrogenic and anti-androgenic effects.

Finally, as shown in Figure 8, the NOAEL of approximately 25 mg/kg/day on the endocrine-related effects of atrazine in males is approximately 10-fold higher than the chronic reference dose currently employed by regulatory authorities to establish acceptable daily intake for atrazine and its chlorometabolites (USEPA, 2011; WHO, 2007). These doses are many orders of magnitude greater than anticipated human exposure.

## 10 Epidemiological studies on testicular and prostate cancers

Published epidemiological studies on the association between atrazine exposure and the incidence of testicular and prostate cancers were reviewed by Sathiakumar et al. (2011) and Boffetta et al. (2013). In the following analyses, we relied on the reviews by Sathiakumar and Boffetta to identify studies published before 2013. We search PubMed for new epidemiological studies on atrazine or the chlorotriazines. We found only two publications reporting data on the association between atrazine use and testicular cancer incidence (Mills, 1998; Remigio et al., 2024). Mills (1998) conducted an ecological study that reported Pearson correlation coefficients between age-adjusted cancer incidence rates in 58 counties in California from 1992 to 1998 and the amount of atrazine used in each county in 1993. They found that for atrazine, there was a non-significant negative correlation (r = −0.16) between the number of pounds of atrazine used in 1992 for each county and testicular cancer reported for white men living in those counties. A non-significant positive correlation (r = 0.41) was reported for Hispanic males. Remigio et al. (2024) conducted an extensive prospective cohort study in North Carolina and Iowa, finding no association between incidence cases that never used atrazine and those that used atrazine and testicular cancer (RR = 1.17; 95% CI (0.57–2.42).

For prostate cancer, based on comparing groups of workers who were ever exposed to the triazines to men who were never exposed, the risk ratios tended to be above one in most studies. However, the results were not statistically significant, except for a significant increase in prostate cancer risk among simazine workers in the Band et al. (2011) study (See Table 7 for the list of studies included in the meta-analysis on the association between atrazine exposure and prostate cancer conducted by Breckenridge et al., 2026). Supplemental Table 7 provides the individual study results and the meta-analyses statistics. The RR’s for atrazine and cyanazine were not significant in the study by Band et al. (2011). The much larger AHS case-control studies on simazine (Pardo et al., 2020) and atrazine (Remigio et al., 2024) were null and close to unity (Supplemental Table 7). The overall meta-analysis risk ratio for Tier 1 studies was 1.0 [95% CI; 0.94–1.16; I2 = 0.46; p < 0.09]. The risk ratio based on two Tier 2 studies was elevated and statistically significant (RR = 1.67; [95% CI; 1.19–2.36]; I2 = 0; p < 0.3; Supplemental Table 7).

**TABLE 7:** Epidemiological studies on prostate cancer. List of studies that were either included or excluded from the meta-analysis shown in Supplementary Table S7.

Study	Study type	Cited by Sathiakumar et al. (2011)	Cited by Boffetta et al. (2013)	Included in meta-analysis
Sathiakumar et al. (1996)	Retrospective follow-up mortality study: Production facility cohort, LA	Yes	No	No^a^
Mills (1998)	Cancer incidence correlation analysis ecologic use proximity, CA	Yes	Yes	No^b^
Alavanja et al. (2003)	AHS prospective cancer incidence study	Yes	Yes	No^c^
MacLennan et al. (2002)	Retrospective follow-up incidence study: Production facility cohort, LA	Yes	Yes	Yes
MacLennan et al. (2003)	Retrospective follow-up mortality study: Production facility cohort, LA	Yes	Yes	No^d^
Mills and Yang (2003)	UFW cohort cancer incidence study on simazine ecologic use proximity, CA	Yes	No	Yes
Hessel et al. (2004)	Nested case-control study on production facility cohort, LA	Yes	Yes	Yes
Rusiecki et al. (2004)	AHS prospective cancer incidence study	Yes	Yes	No^c^
Lynch et al. (2006)	AHS cohort: Cancer incidence study on cyanazine	Yes	No	No^c^
Beane Freeman et al. (2011)	AHS prospective cancer incidence study	No	Yes	No^c^
Band et al. (2011)	Case-control study of agricultural workers in BC based on JME.	No	Yes	Yes
Koutros et al. (2013)	AHS prospective cancer incidence study on atrazine and cyanazine	No	No	No^c^
Pardo et al. (2020)	AHS prospective cancer incidence study on simazine	No	No	Yes
Remigio et al. (2024)	AHS prospective cancer incidence study	No	No	Yes

^a^ Excluded because Sathiakumar et al. (1996) was the earliest retrospective follow-up mortality study of a cohort of production workers. It was updated by MacLennan et al. (2003).

^b^ Excluded because Mills only reported correlation coefficients for four ethnic subgroups.

^c^ Excluded because this AHS, cohort study on prostate cancer was updated by Remigio et al. (2024).

^d^ Excluded because MacLennan et al. (2003) was a Cohort follow-up mortality study; Replaced by MacLennan et al. (2002) which was a cancer incidence study on the same cohort.

The study on company employees at a production facility in Louisiana (MacLennan et al., 2002) had a standard incidence rate (SIR) of 217 (95% CI [94 to 428]) for prostate cancer. This SIR was larger than the SIR for contract production workers (SIR = 129; 95% CI [3–721]) or contract maintenance workers (SIR = 108; 95% CI [13-391]). Hessel et al. (2004) conducted a nested case-control study that found that the difference in prostate cancer risk between company employees and contract workers at the same facility was likely due to company employees participating in a company PSA screening program, whereas contract manufacturers did not. Hessel reported an RR = 8.54 [95% CI = 1.69–82.2] for prostate cancer among individuals at the plant who had at least one PSA test compared to individuals who had no tests. This result suggests that the relative risk for prostate cancer among company employees, reported by MacLennan et al. (2002), was inflated at least in part by a prostate cancer screening bias, as concluded by Hessel et al. (2004).

Remigio et al. (2024) found no effect of the cumulative, intensity-weighted lifetime days of exposure to atrazine on prostate cancer risk, irrespective of whether there was no lag, a 10-year lag, or a 25-year lag between first exposure and disease detection. There were no differences in risk irrespective of whether the cancer was considered “passive” or aggressive prostate cancer (Gleason score ≥ 8; cancer stage ≥ three at diagnosis), or the cancer was considered to have caused death. However, the risk of having aggressive prostate cancer was greater in men less than 60 years of age, and a statistically significant dose trend was evident. These relationships were not evident in men aged 60–69 or those aged 70 years or older.

In an earlier AHS study by Pardo et al. (2020) on pesticide exposure and the incidence of aggressive prostate cancer, 62% of private applicators underwent PSA testing at least once. Pardo et al. (2020) also stated that all men with aggressive prostate cancer were tested for PSA. However, they did not provide the percentage of men with aggressive prostate cancer that had their cancers diagnosed because of elevated PSA results, or whether the PSA test was ordered to confirm other symptoms typically associated with prostate cancer. In neither the study by Pardo nor Remigio was any data provided as to whether there was a differential use of PSA testing between men who were ever exposed to atrazine or simazine vs. men who were never exposed.

Pardo et al. (2020) reported that there was no association between ever vs. never using simazine and aggressive prostate cancer incidence (RR = 1.17; CI 0.93–1.48). This result is consistent with the null results reported by Remigio et al. (2024) for atrazine. Pardo et al. (2020) did not evaluate the relationship between simazine exposure and the age at which aggressive prostate cancer was first detected. Pardo et al. (2020) evaluated the association between ever vs. never using 38 different pesticides, including simazine but not atrazine, and the incidence of aggressive prostate cancer. There was an increased risk of aggressive prostate cancer in one of two contrasts for dimethoate (OR = 1.37; 95% CI = 1.04–1.80) and a decreased risk of aggressive prostate cancer in applicators exposed to triclopyr.

In another study, Beane Freeman et al. (2011) evaluated interactions among single-nucleotide polymorphisms (SNPs) for 149 gene candidates involved in xenobiotic metabolism, prostate cancer, and exposure to 45 different pesticides, including atrazine and cyanazine, but not simazine. Again, the ORs for low and high use of atrazine or cyanazine compared to never used were not statistically significant. They found numerous interactions, including an interaction between a SNP for the glutathione-S-transferase (GST) gene and the OR between cyanazine use and prostate cancer. An interaction between the gene for glutathione synthetase (GSS) and the OR between atrazine use and prostate cancer was also reported. However, the authors concluded that the several positive interactions noted between pesticide use, prostate cancer incidence, and the presence of SNPs for oxidative stress genes and genes involved in phase I/II metabolism in this study were “comparable with the number expected by chance.” In a subsequent study, Koutros et al. (2013) evaluated the association between SNPs associated with prostate cancer susceptibility and pesticide use. Significant interactions were reported for malathion, aldrin, and terbufos. Atrazine, simazine, and cyanazine were not included in the study.

Overall, the conclusion reached by Boffetta et al., in 2013 that “the epidemiological studies do not support a causal relationship between atrazine and prostate cancer” appears to be still appropriate in 2025. The exploratory studies conducted on the AHS cohort appear to be hypothesis-generating. The evaluation of the relationship between aggressive prostate cancer incidence in men less than 60 years of age suffers from having too few men exhibiting this trait among the atrazine cases and controls. This limitation was acknowledged by Beane Freeman et al. (2011) when they stated that they “were not able to explore aggressive cancer alone due to small numbers”. This was evident in the study by Remigio et al. (2024), which found only 17 cases among men who never used atrazine, compared with 21, 23, 19, and 30 men in the atrazine exposure categories Q1 to Q4, respectively.

## 11 Summary and conclusion

The toxicity and carcinogenic potential of atrazine have been extensively studied (Cooper et al., 2007; 2026; Morris-Schaffer et al., 2025; Simpkins et al., 2016; 2026) and closely regulated since its introduction as a pre-emergent herbicide used to control broadleaf grassy weeds primarily in corn (LeBaron et al., 2008). In this review, we evaluated published *in vitro* and *in vivo* studies on atrazine. We focused on characterizing the adverse effects of atrazine on male reproductive organs and defining atrazine doses that had no effect.

Figure 8 illustrates the margin of safety between the toxicologically based (NOAEL) value in animal models and the magnitude of human exposure from food, water, or occupational sources. In this figure, the left-hand y-axis shows atrazine doses (mg/kg/day) from animal studies. The far left-hand panel displays doses used in mechanistic studies in animal models, along with the lowest NOAEL and BMDL for atrazine from a chronic rat study, which regulatory authorities use to calculate human margins of exposure (MOEs). Estimates of human exposure to atrazine are also displayed on the y-axis for production and agricultural workers, as well as for the general public who may be exposed through food or water. Margins of exposure are computed as a ratio of the dose at the NOAEL to the human dose for each exposure scenario. The center insert displays an analysis by Breckenridge et al. (2016) of chlorotriazine exposure in 132 of the most vulnerable community water systems (CWSs) in the US that supply drinking water to the public. The far right-hand axis displays the plasma area under the curve (AUC) that is coupled to an oral dose of atrazine administered to female SD rats in a pharmacokinetic study reported by Simpkins et al. (2026). This study allows one to relate results from *in vitro* atrazine exposure studies to animal and human mg/kg/day doses. The *in vitro* NOAEL for atrazine of 0.05 µM was derived from a study on PDE reported by Simpkins et al. (2026).

It is readily apparent that a large safety margin exists between human exposure and the most sensitive NOAEL from animal models. It is equally apparent that the doses needed to produce effects on male gonadal organ parameters fall within the 25–300 mg/kg range, as observed in MOA studies, and within the upper µM concentrations employed *in vitro*. While some believe that dose-response relationships are sometimes nonmonotonic (Vandenberg et al., 2012), the neuroendocrine system operates according to the law of mass action (Volt et al., 2015). Thus, sufficient concentrations of hormones or hormone mimics are needed to act at the receptor level or at other cellular or molecular targets to modulate function in the complex system that comprises the HPG axis. The evidence review herein does not support the view that atrazine or its chlorometabolites will initiate or sustain cancers in male reproductive organs at doses to which humans will be exposed. This conclusion is consistent with the lack of compelling epidemiological evidence that atrazine causes testicular or prostate cancer in men.

## Glossary

ACTH: adrenocorticotropic hormone
AMM: atrazine metabolic mixture
ATZ: atrazine
AUC: area under the curve
AVPN: anteroventral periventricular nucleus
BPH-1: benign prostate epithelial cell line
BTB: Blood-testes-barrier
CAFs: cancer-associated fibroblasts
cAMP: cyclic adenosine monophosphate
CRH: Corticotropin-Releasing Hormone
CRH-R: Corticotropin-Releasing Hormone Receptor
CYP17A1: cytochrome P450 17A1 (17,20-desmolase)
CYP19A1: cytochrome P450 19A1 (aromatase)
DACT: diaminochlorotriazine
Db-cAMP: dibutyryl cyclic adenosine monophosphate
DEA: deethylatrazine
DHEA: dehydroepiandrosterone
DHT: dihydrotestosterone
DIA: deisopropylatrazine
DNA: deoxyribonucleic acid
DRD2: dopamine D2 receptor
E2: 17β-estradiol
Erβ: estrogen receptor beta
ERK: extracellular signal-regulated kinase
FSH: follicle-stimulating hormone
FSHR: follicle-stimulating hormone receptor
G1 phase: phase 1 of the cell cycle in eukaryotic cell division
G2 phase: growth phase 2 of the cell cycle
GD: gestation day
GnIH: gonadotropin inhibitory neuron
GnRH: gonadotropin-releasing hormone
GPER: G-protein coupled estrogen receptor
GPR30: G-protein coupled estrogen receptor 30
GPR54: kisspeptin receptor
hCG: human chorionic gonadotropin
HPA: hypothalamic-pituitary-adrenal
HPG: hypothalamic-pituitary-gonadal
3β-HSD: 3β-hydroxysteroid dehydrogenase
17β-HSD: 17β-hydroxysteroid dehydrogenase
Kiss1: kisspeptin neurons
Kiss1R: kisspeptin 1 receptor
KNDy: kisspeptin, neurokinin B, and dynorphin-containing neurons
LD50: Lethal Dose, 50%
LH: luteinizing hormone
LHR: luteinizing hormone receptor
MAPK: mitogen-activated protein kinases
mRNA: messenger ribonucleic acid
MTT assay: colorimetric assay for assessing cell metabolic activity
NOEL: no-observed-adverse-effect level
NOAEL: no-observed-adverse-effect level
Pca: prostate cancer cells
PDE: phosphodiesterase
PDE4B: phosphodiesterase 4B
PKA: protein kinase A
PND: Post-natal day
SC: Sertoli cell
SF1: steroidogenic factor 1
SR-B1: Scavenger Receptor Class B Type 1 protein involved in lipid metabolism
StAR: steroidogenic acute regulatory protein that regulates cholesterol transport
TIDA: tubero-infundibular dopaminergic
TSPO: Translocator protein on the outer membrane of mitochondria
