# Supplementary material for: Review of the effect of atrazine on the HPG axes and steroidogenic pathways in males: relevance for testicular and prostate cancer
Source: Front Toxicol. 2026 Mar 11;7:1702389. doi: 10.3389/ftox.2025.1702389 (PMC13012850; doi:10.3389/ftox.2025.1702389)
Supplement: Supplementary file 1 [file Supplementaryfile6.docx]

**Supplemental Figure 6: Survival of Male Rats in the 24-Month Carcinogenicity Study on Atrazine (Mayhew,1986)**

This figure shows individual animal survival in control and atrazine-treated Sprague-Dawley males over a 24-month treatment period. The numbers displayed in the plot are for individual animals in the control (0), the 0.5 mg/kg atrazine dose group (1), the 3.5 mg/kg atrazine dose group (2), the 26 mg/kg atrazine dose group (3), and the 53 mg/kg atrazine dose group (4). A Kaplan-Meier analysis of this survival data indicated that high-dose atrazine-treated males survived significantly longer than untreated control males.
