# Supplementary material for: Review of the effect of atrazine on the HPG axes and steroidogenic pathways in males: relevance for testicular and prostate cancer
Source: Front Toxicol. 2026 Mar 11;7:1702389. doi: 10.3389/ftox.2025.1702389 (PMC13012850; doi:10.3389/ftox.2025.1702389)
Supplement: Supplementary file 2 [file Supplementaryfile1.docx]

Supplemental Figure 1a: Effect of Atrazine on Pituitary, Adrenal & Gonadal Hormones in Adult Male SD Rats (From Foradori et al., 2017)

Histograms of pituitary, adrenal, and gonadal hormone levels (mean ± SEM) in male rats administered 0, 6.5, 25, or 100 mg/kg/day of atrazine for 1, 7, 14, or 28 days.

Pair-fed animals were provided food that match the group mean amount of food consumed by animals in the high-dose atrazine group.

Asterisks denote significant differences in group means from the control group.

**Supplemental Figure 1b: Effect of Single Molar Equivalent Doses of Atrazine, DEA, DIA, or DACT on ACTH, Corticosterone, and Progesterone in Female LE Rats (From Fraites et al. 2009)**


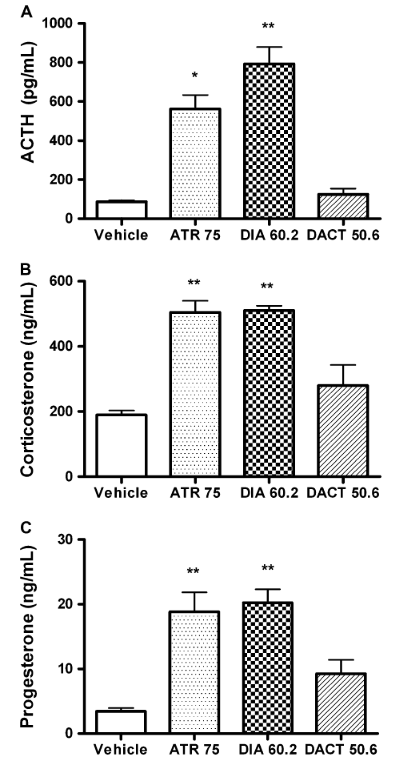


Effect of a single oral dose of 75 mg/kg atrazine or the molar equivalent doses of deethylatrazine (DEA), deisopropylatrazine, (DIA) or diaminochlorotriazine (DACT) on plasma ACTH, corticosterone, and progesterone. Plasma hormone concentrations were evaluated 15 minutes post-dosing. Mean ± SEM; n = 4–7/group.

Statistically significant differences between the group mean of treated and the vehicle control group indicated as *p < 0.001 or **p < 0.0001
