# Supplementary material for: Review of the effect of atrazine on the HPG axes and steroidogenic pathways in males: relevance for testicular and prostate cancer
Source: Front Toxicol. 2026 Mar 11;7:1702389. doi: 10.3389/ftox.2025.1702389 (PMC13012850; doi:10.3389/ftox.2025.1702389)
Supplement: Supplementary file 3 [file Table1.docx]

**Supplemental Table 1: Three-month oral toxicity study on atrazine in TIF:RAIF Rat: Organ weights and histopathological findings in male reproductive organs
(Bachmann, 1994);(Study No. 931063)^1^**

| Endpoint Description | Atrazine (ATZ) Dose Groups | | | |
| --- | --- | --- | --- | --- |
| Group Number | 1 | 2 | 3 | 4 |
| ATZ Concentration in Feed (ppm): | 0 | 10 | 50 | 500 |
| Group Mean ATZ Dose (mg/kg/day): | 0 | 0.6 | 3.3 | 34.1 |
|  |  |  |  |  |
| Group Mean Body Weight (g) (Week 13) | 483.8 | 474.3 | 440.0 | 408.9* |
| Body Weight (Percent of Control) | -- | 98.0% | 90.9% | 84.5% |
| **Organ Weights** |  |  |  |  |
| Testes (grams) | 4.1 | 4.1 | 3.9 | 4.1 |
|  | | | | |
| **Prostate** |  |  |  |  |
| Lymphohistiocytic Infiltration | 0/20 | 0/10 | 0/10 | 1/20 |
| Glandular Atrophy | 1/20 | 0/10 | 0/10 | 2/20 |
|  |  |  |  |  |
| **Testes** |  |  |  |  |
| Atrophy, Tubular | 1/20 | 1/10 | 0/10 | 0/20 |
| Lymphohistocytic Infiltration, Interstitial | 1/20 | 0/10 | 0/10 | 0/20 |
| Hyperplasia, Leydig Cells | 1/20 | 0/10 | 0/10 | 0/20 |

^1^ Epididymis and seminal vesicles were not evaluated in this study.

(*) Statistically different from control by LEPAGE at p<0.01.

Bachmann, M. (1994) 3-month oral toxicity study in rats. Report No. 931063, Ciba-Geigy Ltd. [Unpublished study archived by Syngenta Crop Protection LLC, Greensboro, NC, USA].
