# Supplementary material for: Review of the effect of atrazine on the HPG axes and steroidogenic pathways in males: relevance for testicular and prostate cancer
Source: Front Toxicol. 2026 Mar 11;7:1702389. doi: 10.3389/ftox.2025.1702389 (PMC13012850; doi:10.3389/ftox.2025.1702389)
Supplement: Supplementary file 4 [file Supplementaryfile5.docx]

**Supplemental Figure 5: Effect of Atrazine on RhCG-Stimulated Cyclic AMP and Testosterone in Murine BlTK1 Leydig Cells (From Karmaus & Zacharewski, 2015)**

Concentration-dependent effect of ATR on rhCG-induced steroidogenesis. Intracellular cAMP (A) and T levels in the media (B) were evaluated 4 h after treatment. EC50 values were determined by sigmoidal curve fitting using the ToxResponse Modeler for the co-treatment of 3ng/ml rhCG and 1–600 lM ATR (shaded areas). Data are plotted as the group mean ± SEM and were analyzed by ANOVA followed by Dunnett’s post hoc test, where #P<.05 versus rhCG.
