# Supplementary material for: Review of the effect of atrazine on the HPG axes and steroidogenic pathways in males: relevance for testicular and prostate cancer
Source: Front Toxicol. 2026 Mar 11;7:1702389. doi: 10.3389/ftox.2025.1702389 (PMC13012850; doi:10.3389/ftox.2025.1702389)
Supplement: Supplementary file 5 [file Table7.docx]

**Supplemental Table 7: Meta-Analyses of prostate cancer risk for Tier 1 & 2 studies and & Tier 1 & Tier 2 studies combined.**

RR= Relative Risk, LCL or UCL = Lower bound (LCL) and Upper bound (UCL) Limit of the 95^th^ percentile confidence interval (CI).

I^2^ = Percentage of between-study variance in the RRs attributed to study heterogeneity (Calculated as described by Breckenridge et al. 2016).
