# Supplementary material for: Review of the effect of atrazine on the HPG axes and steroidogenic pathways in males: relevance for testicular and prostate cancer
Source: Front Toxicol. 2026 Mar 11;7:1702389. doi: 10.3389/ftox.2025.1702389 (PMC13012850; doi:10.3389/ftox.2025.1702389)
Supplement: Supplementary file 7 [file Table2.docx]

**Supplemental Table 2. Incidence of Tumors in the Testes, Epididymis, Seminal Vesicles & Prostate of Sprague-Dawley Rats Exposed to Atrazine in the Feed for 24-Months (Study No. 410-1102; Mayhew, 1986)**

| Organ | Atrazine Concentration in Feed (ppm): | 0 | 10 | 70 | 500 | 1000 |
| --- | --- | --- | --- | --- | --- | --- |
|  | Group Mean Atrazine Dose (mg/kg/day): | 0 | 0.50 | 3.51 | 25.9 | 53.4 |
| Testes | Malignant mesothelioma | 1/65 | 0/65 | 0/67 | 0/67 | 0/67 |
|  | Interstitial cell tumor | 1/65 | 3/65 | 2/67 | 2/67 | 7/67*^a,b^ |
|  | Hemangioma | 0/65 | 0/65 | 0/67 | 0/67 | 1/67 |
|  | Malignant lymphoma | 0/65 | 2/65 | 1/67 | 0/67 | 0/67 |
|  | Histiocytic sarcoma | 0/65 | 1/65 | 0/67 | 0/67 | 0/67 |
|  | Seminoma | 0/65 | 1/65 | 0/67 | 0/67 | 0/67 |
|  | Interstitial cell hyperplasia | 1/65 | 0/65 | 0/67 | 1/67 | 2/67 |
|  |  |  |  |  |  |  |
| Epididymis | Malignant mesothelioma | 1/65 | 0/65 | 0/67 | 0/67 | 0/67 |
|  | Malignant lymphoma | 1/65 | 3/65 | 1/67 | 0/67 | 0/67 |
|  | Granulocytic leukemia | 0/65 | 0/65 | 0/67 | 1/67 | 0/67 |
|  | Histiocytic sarcoma | 0/65 | 1/65 | 0/67 | 0/67 | 0/67 |
|  |  |  |  |  |  |  |
| Seminal Vesicle | Malignant mesothelioma | 1/65 | 0/65 | 0/67 | 0/67 | 0/67 |
|  | Granulocytic leukemia | 0/65 | 0/65 | 0/67 | 1/67 | 0/67 |
|  | Malignant lymphoma | 0/65 | 3/65 | 1/67 | 0/67 | 0/67 |
|  | Papillary adenoma | 0/65 | 0/65 | 1/67 | 0/67 | 1/67 |
|  | Histiocytic sarcoma | 0/65 | 1/65 | 1/67 | 0/67 | 0/67 |
|  |  |  |  |  |  |  |
| Prostate | Malignant mesothelioma | 1/65 | 0/63 | 0/66 | 0/67 | 0/66 |
|  | Malignant lymphoma | 1/65 | 3/63 | 1/66 | 0/67 | 0/66 |
|  | Adenoma | 0/65 | 0/65 | 1/66 | 1/67 | 0/66 |
|  | Granulocytic leukemia | 0/65 | 0/65 | 0/66 | 1/67 | 0/66 |
|  | Histiocytic sarcoma | 0/65 | 1/65 | 1/66 | 0/67 | 0/66 |
|  | Epithelial hyperplasia | 12/65 | 16/65 | 1/66 | 17/67 | 29/66** |

*Significantly different from the control group incidence based on the Chi-square test with a Yates correction (p < 0.05).

^a^ This difference was no longer after a statistical correction for increased survival of the high-dose atrazine group (See next page)

^b^ The incidence of interstitial cell tumors in this group was within the laboratory historical control range of 0 -11.7 %, Mean = 5.55.

**Significantly different from the control group incidence based on the Chi-square test with a Yates correction (p < 0.01).

Mayhew, A. Dale., 1986. Two-year chronic feeding/oncogenicity study in rats administered atrazine. Rep. N°: 410-1102. American Biogenics Corporation, 29.04.1986, Unpublished study archived by Syngenta Crop Protection LLC, Greensboro, NC, USA, MRID 00158930.
