# Supplementary material for: Review of the effect of atrazine on the HPG axes and steroidogenic pathways in males: relevance for testicular and prostate cancer
Source: Front Toxicol. 2026 Mar 11;7:1702389. doi: 10.3389/ftox.2025.1702389 (PMC13012850; doi:10.3389/ftox.2025.1702389)
Supplement: Supplementary file 8 [file Table5.docx]

**Supplemental Table 5: Histopathological Findings in Male Reproductive Organs from an Eighteen-Month Carcinogenicity Study on Atrazine in CD-1 Mice (Study 842120; Hazelette & Green, 1987)**

| **Organ** | **Atrazine Concentration in Feed (ppm):** | **0** | **10** | **300** | **1500** | **3000** |
| --- | --- | --- | --- | --- | --- | --- |
|  | **Group Mean Atrazine Dose (mg/kg/day):** | **0** | **1.2** | **38.4** | **194.0** | **385.7** |
|  | Group Mean Body Weight (g) (Week 91) | 38.5 | 40.2 | 36.7 | 35.6* | 35.5** |
| Testes | Embryonal Carcinoma | 0/57 | 0/59 | 0/59 | 0/59 | 1/57 |
|  | Gonadal Stromal Tumor, Malignant | 0/57 | 0/59 | 1/59 | 0/59 | 0/57 |
|  | Amyloid | 5/57 | 3/59 | 4/59 | 3/59 | 6/57 |
|  | Atrophy | 10/57 | 8/59 | 14/59 | 12/59 | 17/57 |
|  | Giant Cells | 0/57 | 1/59 | 0/59 | 0/59 | 0/57 |
|  | Mineralization | 0/57 | 3/59 | 3/59 | 0/59 | 0/57 |
|  | Periarteritis Nodosa | 1/57 | 0/59 | 0/59 | 0/59 | 0/57 |
| Epididymis | Amyloid | 0/58 | 1/60 | 0/60 | 0/59 | 1/58 |
|  | Seminal Plug | 0/58 | 1/60 | 1/60 | 0/59 | 1/58 |
|  | Spermatic Granuloma | 0/58 | 0/60 | 1/60 | 0/59 | 1/58 |
| Seminal Vesicle | Acute Purulent Inflammation | 0/58 | 2/60 | 0/60 | 0/60 | 0/58 |
|  | Chronic Lymphocytic Inflammation | 1/58 | 0/60 | 0/60 | 0/60 | 0/58 |
|  | Chronic Purulent Inflammation | 0/58 | 0/60 | 0/60 | 1/60 | 0/58 |
|  | Dilatation | 11/58 | 8/60 | 2/60 | 4/60 | 0/58 |
|  | Empty | 0/58 | 2/60 | 1/60 | 2/60 | 1/58 |
|  | Hemorrhage, General | 1/58 | 0/60 | 0/60 | 0/60 | 0/58 |
|  | Subacute Purulent Inflammation | 0/58 | 1/60 | 0/60 | 0/60 | 0/58 |
| Prostate | Chronic Purulent Inflammation | 0/59 | 0/60 | 1/60 | 1/60 | 0/58 |
|  | Subacute Purulent Inflammation | 2/59 | 2/60 | 1/60 | 3/60 | 1/58 |

Hazelette, J.R. and Green, J., 1987. G 30027 - Oncogenicity study in mice, Ciba-Geigy Corp., Research Department, Pharmaceuticals Division, Summit, NJ, USA, Rep. N°: 842120, 30.10.1987, Unpublished study archived by Syngenta Crop Protection LLC, Greensboro, NC, USA, MRID 40431302.
