# Supplementary material for: Review of the effect of atrazine on the HPG axes and steroidogenic pathways in males: relevance for testicular and prostate cancer
Source: Front Toxicol. 2026 Mar 11;7:1702389. doi: 10.3389/ftox.2025.1702389 (PMC13012850; doi:10.3389/ftox.2025.1702389)
Supplement: Supplementary file 9 [file Supplementaryfile7.docx]

**Supplemental Figure 7: No Effect of Atrazine on ROS Production (H_2_O_2_) by H295R, JEG-3, H-22, and MCF-7 Cells^1^ (Data from Simpkins et al. (2026).**
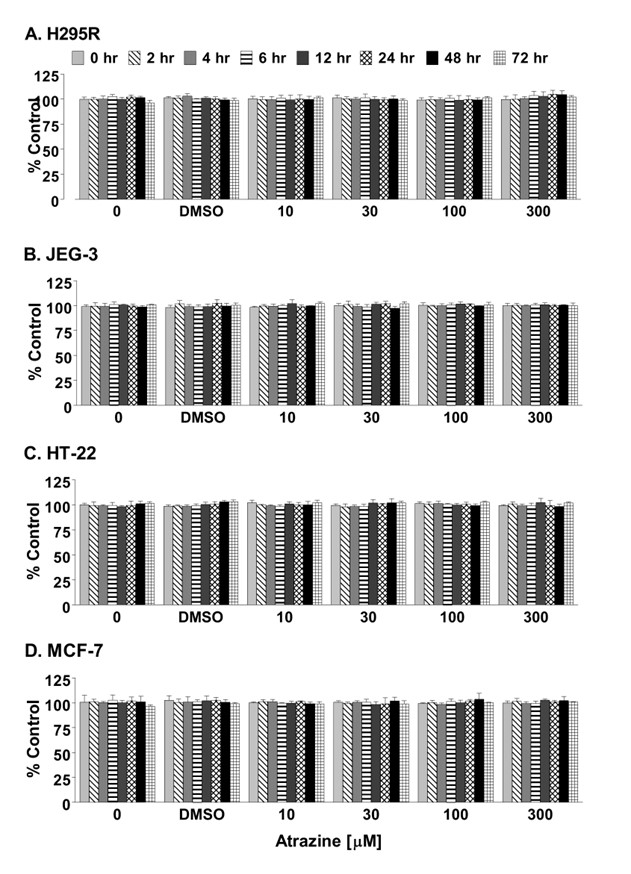


**^1^**H295R, JEG-3, HT-22, or MCF-7 cells were seeded into 96-well plates 24 hours before treatment. Atrazine was dissolved in DMSO and added to the media. ROS production was monitored after 72 hrs. Mean ±SEM) is reported as a percentage of ROS production in untreated control wells. Six independent experiments were conducted in triplicate for each concentration evaluated.
