# Supplementary material for: Review of the effect of atrazine on the HPG axes and steroidogenic pathways in males: relevance for testicular and prostate cancer
Source: Front Toxicol. 2026 Mar 11;7:1702389. doi: 10.3389/ftox.2025.1702389 (PMC13012850; doi:10.3389/ftox.2025.1702389)
Supplement: Supplementary file 10 [file Table3.docx]

**Supplemental Table 3: Incidence of Histopathological Findings in the Testes, Epididymis, Seminal Vesicles & Prostate of the Fischer-344 Rat after 24 Months of Treatment (Thakur 1992; Study 483-277 & FR1 (Stevens et al., 1999)**

| Organ | Atrazine Concentration in Feed (ppm): | 0 | 10 | 70 | 200 | 400 |
| --- | --- | --- | --- | --- | --- | --- |
|  | Group Mean Atrazine Dose (mg/kg/day)1: | 0 | 0.54 | 3.85 | 11.04 | 21.87 |
| Testes | Hypospermia | 58/60 | 56/60 | 59/60 | 60/60 | 55/60 |
|  | Interstitial Cell Hyperplasia | 5/60 | 7/60 | 2/60 | 2/60 | 6/60 |
|  | Interstitial Cell Tumor (B) | 58/60 | 57/60 | 58/60 | 60/60 | 56/60 |
|  | Mesothelioma (M) | 2/60 | 2/60 | 1/60 | 2/60 | 2/60 |
|  | Fibrosarcoma | 0/60 | 1/60 | 0/60 | 0/60 | 0/60 |
|  | Hematopoietic Neoplasia | 1/60 | 2/60 | 2/60 | 1/60 | 0/60 |
|  |  |  |  |  |  |  |
| Epididymis | Immature/Abnormal Sperm | 9/60 | 4/14 | 3/15 | 2/16 | 5/60 |
|  | Hypospermia | 52/60 | 9/14 | 13/15 | 16/16 | 53/60 |
|  | Fibrosarcoma | 0/60 | 1/14 | 0/15 | 0/16 | 0/60 |
|  | Hematopoietic Neoplasia | 1/60 | 0/14 | 1/15 | 1/15 | 1/15 |
|  |  |  |  |  |  |  |
| Seminal Vesicle | Decreased Secretion | 56/59 | 44/46 | 47/48 | 43/47 | 54/60 |
|  | Increased Secretion | 0/59 | 0/46 | 1/48 | 2/47 | 0/60 |
|  | Chronic Active Inflammation | 0/59 | 0/46 | 0/48 | 0/47 | 1/60 |
|  | Focal Hyperplasia | 0/59 | 0/46 | 1/48 | 0/47 | 0/60 |
|  | Hematopoietic Neoplasia | 0/59 | 1/46 | 2/48 | 2/47 | 1/60 |
|  |  |  |  |  |  |  |
| Prostate | Acute Inflammation | 0/60 | 0/17 | 1/16 | 0/17 | 0/60 |
|  | Chronic Inflammation | 26/60 | 9/17 | 5/16 | 10/17 | 26/60 |
|  | Adenoma | 0/60 | 0/17 | 0/16 | 0/17 | 0/60 |
|  | Adenocarcinoma | 0/60 | 0/17 | 0/16 | 0/17 | 0/60 |

| Group | Group No. | Dose  (mg/kg/day |
| --- | --- | --- |
| Control | 0 | 0 |
| Atrazine | 1 | 0.5 |
|  | 2 | 3.5 |
|  | 3 | 26 |
|  | 4 | 53 |

Thakur, A.K. (1992b) Oncogenicity study in Fischer-344 rats with atrazine. Report No. 483-277, Hazleton Laboratories. [Unpublished study archived by Syngenta Crop Protection LLC, Greensboro, NC, USA].

Stevens J.T., Breckenridge C.B., Wetzel L., Thakur A.K., Liu C., Werner C., et al. (1999). A risk characterization for atrazine: oncogenicity profile. J Toxicol Environ Health A., 56(2):69-109.
