# Supplementary material for: Review of the effect of atrazine on the HPG axes and steroidogenic pathways in males: relevance for testicular and prostate cancer
Source: Front Toxicol. 2026 Mar 11;7:1702389. doi: 10.3389/ftox.2025.1702389 (PMC13012850; doi:10.3389/ftox.2025.1702389)
Supplement: Supplementary file 11 [file Supplementaryfile8.docx]

**Supplemental Figure 8.1: Proposed Adverse Outcome Pathway for the Effects of Atrazine on the HPG Axis in Males**

**Supplemental Figure 8.2: Proposed Adverse Outcome Pathway for the Effects of Atrazine on the HPA Axis in Males**

**Supplemental Figure 8.3: Proposed Adverse Outcome Pathway for Atrazine and the Interaction between its Effects on the HPA and HPG Axes in Males**

**Supplemental Figure 8.4: Proposed Adverse Outcome Pathway for the Effect of Atrazine Phosphodiesterase**

**Supplemental Figure 8.5: Proposed Mechanism of Atrazine-Induced Redox Imbalance (From Abarikwu et al., 2023)**

**Supplemental Figure 8.6: Proposed AOP for Atrazine Role in the Production of Reactive Oxygen Species (ROS)**
