# Supplementary material for: Review of the effect of atrazine on the HPG axes and steroidogenic pathways in males: relevance for testicular and prostate cancer
Source: Front Toxicol. 2026 Mar 11;7:1702389. doi: 10.3389/ftox.2025.1702389 (PMC13012850; doi:10.3389/ftox.2025.1702389)
Supplement: Supplementary file 12 [file Table4.docx]

**Supplemental Table 4: Incidence of Histopathological Findings in the Testis of Male SD Rats after 8 or 52 Weeks of Treatment with Atrazine^1^ (Study 852214, Rudzki, 1991)**

| Parameter | Control | Atrazine Dose (ppm) | | |
| --- | --- | --- | --- | --- |
|  | 0 | 10 | 50 | 500 |
| Week 8 | | | | |
| Dose Level (mg/kg/day): 1 to 8 Weeks | 0 | 0.8 | 4.1 | 37.4 |
| Mean Body Weight at Week 8 (g) | 640 | 522 | 530 | 456** |
| Testes: Unilateral Atrophy | 1/10 | 3/10 | 2/10 | 1/10 |
| Testes: Sperm Granuloma | 0/10 | 1/10 | 0/10 | 0/10 |
| Week 52 | | | | |
| Dose (mg/kg/day): 1 to 52 Weeks | 0 | 0.5 | 2.3 | 23.6 |
| Mean Body Weight at Week 52 (g) | 783 | 776 | 775 | 651** |
| Testes: Granulocytic Leukemia | 1/40 | 0/40 | 0/40 | 0/40 |
| Testes: Unilateral + Bilateral Atrophy | 8/40 | 8/40 | 7/40 | 6/39 |
| Testes: Edema | 4/40 | 3/40 | 0/40 | 6/39 |
| Testes: Hyalinization Unilateral | 0/40 | 0/40 | 1/40 | 0/39 |
| Testes: Periarteritis | 0/40 | 0/40 | 0/40 | 1/39 |
| Testes: Unilateral + Bilateral Sperm Granuloma | 3/40 | 0/40 | 0/40 | 1/39 |

^1^ Males in this study were the second-generation offspring from a multi-generation reproduction study. Mainiero et al., 1987). F2 generation males were exposed to atrazine via their dams throughout gestation and lactation and subsequently from either PND 0 to PND 56 (8-week subgroup) or from PND0 to PND 365 (52-week subgroup).

*Significantly different from the control group (p < 0.01

Rudzki, M. W., McCormick, G. C. and Arthur, A. T., 1991, Chronic toxicity study in rats (derived from 2-gen.), Ciba-Geigy Corp., Research Department, Pharmaceuticals Division, Summit, NJ, USA, Rep. N°: 852214, 28.1.1991, . [Unpublished study archived by Syngenta Crop Protection LLC, Greensboro, NC, USA, MRID 44005301.
