# Supplementary material for: Review of the effect of atrazine on the HPG axes and steroidogenic pathways in males: relevance for testicular and prostate cancer
Source: Front Toxicol. 2026 Mar 11;7:1702389. doi: 10.3389/ftox.2025.1702389 (PMC13012850; doi:10.3389/ftox.2025.1702389)
Supplement: Supplementary file 13 [file Supplementaryfile2.docx]

**Supplemental Figure 2a: Pulsatile LH in Ovariectomized Female Wistar Rats Administered Atrazine for 4 Days at Doses of 0, 50, 100, or 200 mg/kg/day (From Foradori et al., 2009)^1^.**

**Panel A: Representative Single Animals Panel B: Mean LH Pulse Period and Maximum Amplitude**

**^
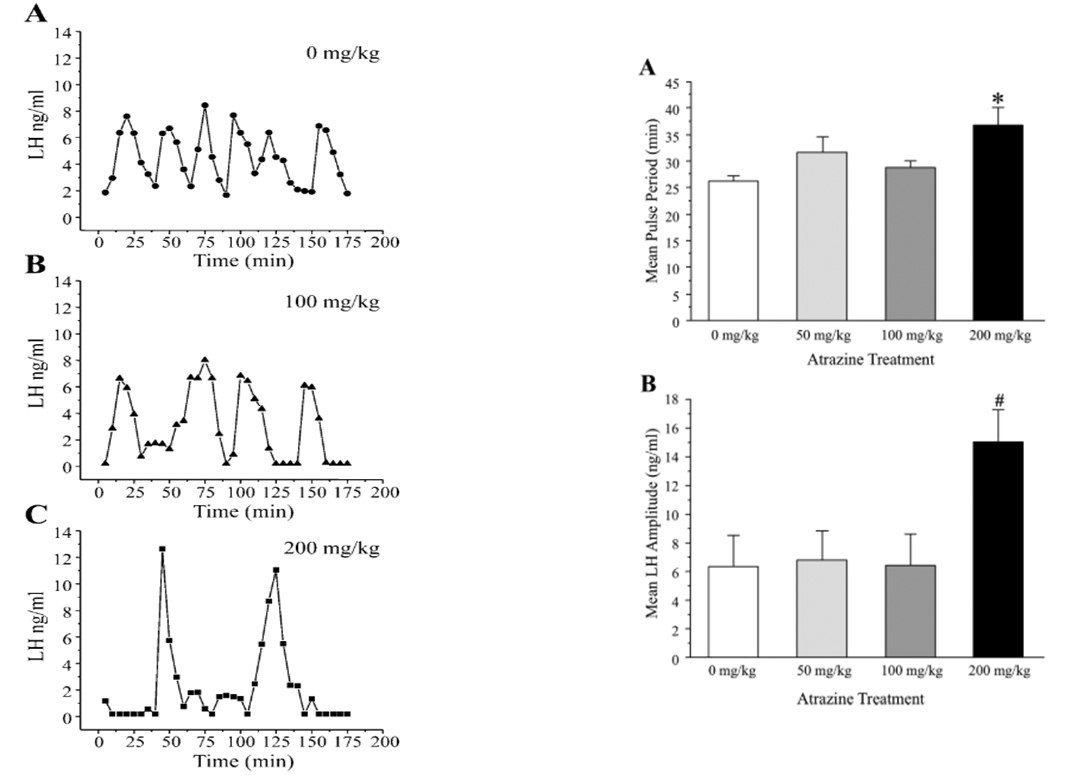
^**

^1^Young adult female Wistar rats were single-housed with a 12-hour light: dark cycle with ad libitum access to food and water. After acclimatization, all animals were ovariectomized under anesthesia, followed by a 7 to 8-day recovery. A jugular cannula was implanted to permit serial blood collection from atrazine-treated rats (0, 50, 100, or 200 mg/kg/day administered by gavage for 4 days). Three to four hours after the last atrazine dose, 150 µl of blood was collected repeatedly over a 3-hour period and analyzed for LH by radioimmunoassay.

Panel A shows plots of individual animal LH concentrations measured over time for rats administered atrazine at doses of 0, 100, or 200 mg/kg/day.

Panel B provides the group mean (± SEM; N= 5 to 6) LH pulse-period (minutes) (Figure A) and the maximum LH pulse amplitudes (Figure B.

The results indicate that an atrazine dose of 100 mg/kg/day may decrease pulse frequency in individual animals. However, reduced LH pulse frequency and increased maximum LH amplitude were statistically significantly different from controls in only the 200 mg/kg/day atrazine-treated group. The NOAEL in this study was 100 mg/kg/day.

**Supplemental Figure 2b: Effects of Adrenalectomy on Pulsatile LH in Ovariectomized Female Wistar Rats Administered Atrazine for 4 Days at Doses of 200 mg/kg/day (Panel A & B) or 0, 50, or 200 mg/kg/day (Panel C & D; From Foradori et al., 2011)^2^.**


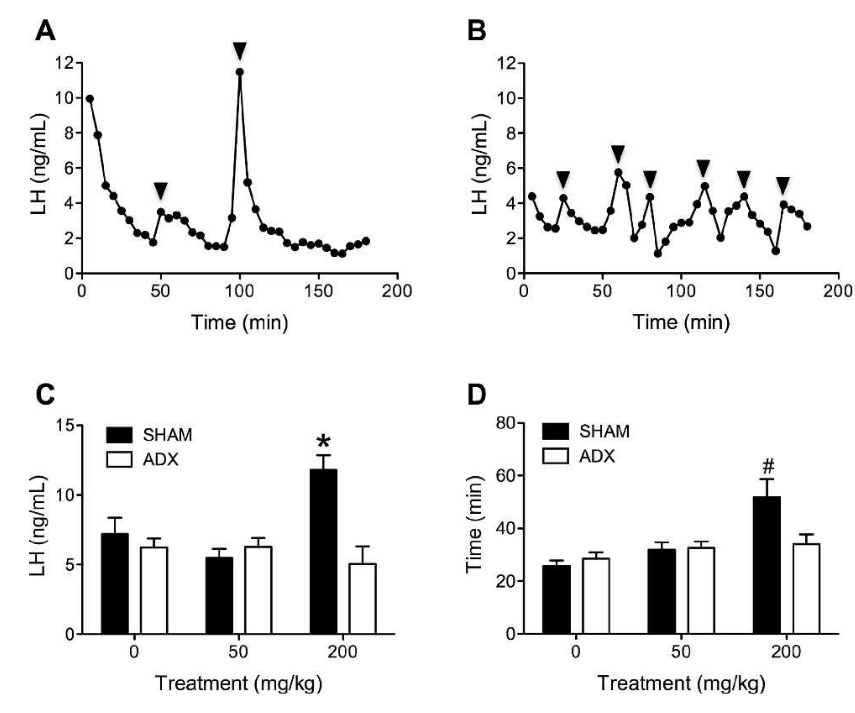


^2^LH levels in adrenalectomized or sham-operated ovariectomized female Wistar rats after 4 daily atrazine doses of 0, 50, or 200 mg/kg/day.

Figure A shows the LH concentration measured every 5 minutes for an individual sham-operated rat administered 200 mg/kg/day atrazine.

Figure B is the LH concentration for an individual adrenalectomized 200 mg/kg/day atrazine-treated rat.

Figure C is a plot of the group mean (±SEM) LH pulse peak concentration in atrazine-treated (0, 50, or 200 mg/kg/day) sham-operated (black bars) or adrenalectomized (white bars) rats.

Figure D is a plot of the group mean LH pulse period (±SEM) in sham (black bars) for the same animals displayed in Figure C.

N = 9–13 rats/group,

*P < 0.05 for the atrazine-treated high dose group (200 mg/kg) vs. all other groups;

^#^P < 0.05 for the atrazine-treated high dose group (200 mg/kg/day) vs. the control group (0 mg/kg/day).
