# Supplementary material for: Review of the effect of atrazine on the HPG axes and steroidogenic pathways in males: relevance for testicular and prostate cancer
Source: Front Toxicol. 2026 Mar 11;7:1702389. doi: 10.3389/ftox.2025.1702389 (PMC13012850; doi:10.3389/ftox.2025.1702389)
Supplement: Supplementary file 14 [file Table6.docx]

**Supplemental Table 6a: Effects on the Cell Viability, Proliferation, and ROS Production *In Vitro:* Male Reproductive Cells^1^**

| **Study Author** | **Cell ID**  **Type** | **ATZ Conc. (µM)**  **Duration (hours)** | **Cytotoxicity or Cell Viability Indicators: MTT, NRU, TBE, LDH** | **Cell Proliferation or Apoptosis Indicators**  **MMP** | **Cell Proliferation**  **(Optical Density (OD)**  **of Cell Cultures)** | **ROS: H_2_0_2_, NO^-^, O^-^**  **Oxidative Stress Indicators:**  **MDA, GSH, GSH-Pxs, GR, GST,**  **CAT, SOD, Trx, ↑CYP450s** |
| --- | --- | --- | --- | --- | --- | --- |
| **Robitaille (2015)** | **LNCaP**  **Human Prostate Cancer** | **1, 3, 10, 30**  **24 hours** | **----** | **NE^a^** | **----** | **----** |
| **Robitaille (2015)** | **LNCaP**  **Human Prostate Cancer** | **1, 3, 10, 30**  **+ 0.1 or 0. 3 nM DHT** | **----** | **NE^a^** | **----** | **----** |
| **Hu (2016)** | **RM1**  **Mouse Prostate Cancer Cell** | **0.01, 0.1**  **(24, 48, or 72)** | **↑ MTT @ ≥ 0.01 µM** | **↑ Cell Cycle @ ≥ 0.01 µM** | **↑ OD @ ≥ 0.01 uM**  **In Vitro** | **----** |
| **Hu (2016)^b^** | **RM1 Explant in**  **ATZ-dosed C57BL6 mice** | **20, 100 mg/kg/day**  **15 daily doses** | **----** | **↑ Tumor Cell Cycle ex vivo @ ≥ 20 mg/kg/day** | **↑ Tumor Weight: *In Vivo* @ 100 mg/kg/day**  **↑ Cell Migration: *In Vivo* @ ≥ 20 mg/kg** | **----** |
| **Abarikwu (2011a)** | **Leydig Cells**  **Rat Primary Culture** | **2.32, 23.2, 46.4; 116, 232**  **(24, 48, 72 hours)** | ↓ **MTT:** **≥ 116 µM: 48h**  ↓ **NRU: 232 µM: 72h**  **NOEL = 46.4 µM** | **----** | **----** | **----** |
| **Abarikwu (2013a, b)** | **Leydig Cells**  **Rat Primary Culture** | **2.32, 23.2, 46.4**  **116, 232, 464, 928**  **(24, 48, 72 hours)** | **Dose-Dependent**  ↓ **MTT:** **≥ 116 µM**  **↑ LDH Leakage**  **NOEL = 46.4 µM: 48h** | **----** | **----** | **↑ ROS: @ 46.4 & 116 µM: 3, 5 h**  **↑ MDA, GSH, GSH-Px, GR, GST**  **↓ SOD, GST @ 46.4 & 116 µM**  **NOEL = 23.2 µM** |
| **Pogrmic-Majkic (2012)** | **In vivo dosing Wistar rats**  **Ex vivo Leydig Cell Analysis** | **50, 200 mg/kg**  **Days 23-51** | **---** | **Testes & Liver: Lipid peroxidation & SOD: NE**  **↓CAT & GSH-Px @ 200 mg/kg**  **Testes Alone: ↓GST50 @ 200 mg/kg**  **Liver Alone: ↑GST 200 µM; CYP1A1/2, CYP2B: NE** | | |
| **Mgbudom-Ohah (2024)** | **TM3**  **Mouse Leydig cells** | **50 mg/kg/day**  **6 days/week for 52 weeks** | **Leydig Cell Viability: Increased in ATZ-treated mice with testosterone vs. ATZ-treated mice without testosterone.**  **(Testosterone Dose: 200, 400, 800 ug/kg/day; 2x/week; 52 weeks)** | | | |
| **Mgbudom-Ohah (2024)** | **TM4**  **Mouse Sertoli cells** | **50 mg/kg/day**  **6 days/week for 52 weeks** | **Sertoli Cell ROS: Decreased in ATZ-treated mice with testosterone vs. ATZ-treated mice without testosterone**  **(Testosterone Dose: 200, 400, 800 ug/kg/day; 2x/week; 52 weeks)** | | | |
| **Chevalier (2016a, b)** | **JKT-1**  **Human Seminoma** | **10^-5^ to 10^-12^ M** | **----** | **~ 25% ↓ 10^-7^ M** | **----** |  |

^1^ Only statistically significant differences are summarized in Supplemental Table 6. Data for effects on signaling pathways are not reported herein

**NE:** No Effect

^a^ 96-Well Plate; WST-1 Kit (CCK-8 assay. There were no effects of atrazine on prostate-specific antigen (PSA) secretion.

^b^ Dose-dependent increased cell migration reported ex vivo at doses of 20 and 100 mg/lg/day

**Supplemental Table 6b: Effect on Steroidogenesis, ROS, Antioxidants & Mitochondria in Cell Cultures or In Vivo^1^**

| **Study Author** | **Cell Type**  **Source** | **ATZ Conc (µM)**  **Exposure Duration (hours)** | **Steroidogenesis (mRNA/enzyme)** | **H_2_O_2,_ GSH,**  **MDA, SRB** | **Antioxidants**  **GP, GR, GST, SOD** | | **GSH-Pxs, GR, SOD** | **Mitochondria** |
| --- | --- | --- | --- | --- | --- | --- | --- | --- |
| **Yuan, (2017)^2^** | **Oocytes**  Porcine | **0, 50, 100, 200, or 500**  **(Ex vivo)** | **----** | ↑200 uM | ↓200uM | | **----** | ↑200 µM |
| **Fa (2013)** | **Granulosa cells**  **Rat** | **10, 20**  **(48 hours)** | **FSH- stimulated mRNA Expression**  **↑ E2, LHR at 10μM,**  **↓ E2, LHR & CYP19A1, EREG, AREG and PgR at 20μM** | **SRB: NE** | **----** | | **----** | **----** |
| **Pogrmic-Majkic, (2014)** | **Granulosa cells** | **20**  **(48 hours)** | **FSH-stimulated mRNA Expression**  **↑ P4, E2, Star, Cyp11a1 at 20 μM,** | **SRB: NE** | **----** | | **----** | **----** |
| **Pogrmic-Majkic, (2018)** | **Granulosa cells**  **Human IVF** | **0.01, 0.1, 1, 20**  **(48 hours)** | **↓ FSH induced mRNA Expression of E2, PR, STAR, CYP19A1 LHCGR, EREG, AREG only 20 μM ATR**  **ATR (20 μM increased FSH-stimulated cPDE** | **SRB: NE** | **----** | | **----** | **----** |
| **Abarikwu (2011a)** | **Leydig cells**  **Rat** | **23.2. 46.4, 116, 232, 924**  **(24, 48 or 72 hours)** | **cAMP-induced mRNA Expression:** ↓StAR, CYP11A1, 3ꞵHSD,  **48 & 72 hrs. @ 116 & 2**32 μM; NOEL = 46.4 µM | **----** | **----** | | **----** | **----** |
| **Abarikwu (2013a, b)^a^** | **Leydig cells**  **Rat** | **23.2, 46.4, 116, 232, 464, 927**  **Ex Vivo** | ↑ 17ꞵHSD 3ꞵHSD @ 232 uM; | **↑ 46.4, 116 uM** | **↓46.4, 116 µM** | | ↓ **232 µM** | **----** |
| **Study Author** | **Cell Type**  **Source** | **ATZ Conc (µM)**  **Exposure Duration (hours)** | **In Vivo Findings on PND 51 (Pogrmic 2009)**  **or Day 16 (Farombi, 2013)** | **Ex Vivo Findings** | | | | |
| **Pogrmic (2009)** | **Wistar Rat**  **Leydig Cells**  **Wistar Rat** | **In Vivo Dosing: PND 23-51**  **50 or 200 mg/kg/day**  **Ex Vivo (0.001-50 μM),**  **1, 3 or 6-day cell culture** | **PND 51**  Serum LH: NE  ↓ Body Weight @ Atz dose of 200 mg/kg/day  ↑ Adrenal Gland Wt. @ 200 mg/kg  ↓ T, DHT @ 200 mg/kg/day  ↓ Testis, Seminal Vesicle, Prostate Wts. @ 200 mg/kg/day  ↓ Dorsal Prostate Wt. @ 200 mg/kg/day | **Day 1**  **Basel cAMP:** ↑ (1, 20 µM)  **hCG-stimulated:** ↑ 20 µM  **Basel DHT**: ↑ (1-50 µM)  **Basel P4:** ↑ (0.001, 1 µM)  **mRNA:** ↑20 µM LRH, SF-1,StAR, TSPO, PDE4B, 3BHSD,17BHSD CYP17A1 | | | **Day 3 vs. Day 1**  **↑cAMP, DHT**  **@ 20 µM** | **Day 6 vs Day 1**    **↓cAMP, DHT**  **@ 20 µM** |
| **Study Author** | **Cell Type**  **Source** | **ATZ Conc (µM)**  **Exposure Duration (hours)** | **In Vivo or In Vitro Findings** | **Ex Vivo Findings** | | | | |
| **Pogrmic-Majic (2016)** | **Leydig cells**  **Wistar Rat** | **50 μM**  **30, 60 or 120 min** | **↑T + DHT ATR alone at 120; ↑cAMP↑StAR**  **↑hCG + ATR 50μM at 60 & 120 min** | **----** | **----** | | **----** | **----** |
| **Farombi (2013)** | **Male**  **Wistar Rats** | **120 mg/kg/day**  **peripubertal: 16 days** | **Day 16**  **↓ BW, testis, epididymis, seminal vesicle, and prostate Wts (both absolute and relative). ↑abnormal sperm** | **Testis**  ↑MDA; ↓GST, SOD | | **Epididymis**  ↑MDA; GSH, SOD  ↓GST, CAT, SOD, AA | | |

^1^ Only statistically significant differences are summarized in this table**.** ^2^ Also reported increased ROS products and decreased oocyte proliferation *in vitro* at 200 uM.

**Supplemental Table 6c: Effects on Cell Viability, Proliferation, & ROS Production *In Vitro:* Other Cell Types**

| **Study Author** | **Cell ID**  **Type** | **ATZ Conc. (µM)**  **Duration (hours)** | **Cytotoxicity/ Viability**  **MMT, TBE, NRU,**  **LDH Release, OD** | **Cell Proliferation/**  **Apoptosis: MMP** | **Cel Proliferation**  **(Cell Cycle) or Cell Density (OD)** | **Oxidative Stress**  **H_2_O_2_, MDA** |
| --- | --- | --- | --- | --- | --- | --- |
| **Albanito (2015)** | **BG**  **Human Ovarian Cancer** | **1 µM**  **(1 hour)** | **----** | **----** | **~ 260% ↑1 µM** | **----** |
| **Albanito (2015)** | **2008**  **Human Ovarian Cancer** | **1 µM**  **(1 hour)** | **----** | **----** | **~ 250% ↑1 µM** | **----** |
| **Kmetic (2008)** | **CHO-K1, CCL-61**  **Chinese Hamster Ovary - Epithelial** | **10, 20, 40, 60, 80, 160**  **24, 48, 72 hours** | **TBE: ↓ ≥ 20** | **IC20 (20% decreased cell viability 72 hr)**  **TBE = 101 µM; NRU = 212 µM;**  **KB = 241 µM; MTT = 290 µM** | | **----** |
| **Li (2024)** | **GC**  **Quail Ovarian Granulosa Cell** | **250** |  | **~ 25% ↓** | **----** | **NE (H_2_O_2_)**  **↑ (MDA)** |
| **Albanito (2015)** | **CAF**  **Cancer Associated Fibroblasts** | **1 µM** | **----** | **Increased Transwell Cell Migration** | | **----** |
| **Oh (2003)** | **MCF-7 BUS**  **Human Breast Cancer** | **10^-5^ to 10^-14^ M** | **----** | **NE** | **----** | **----** |
| **Wang (2023)** | **4T1**  **Mouse Breast Cancer** | **0.01, 0.1**  **24, 48 hours** | **----** | **MMP: ↑** | **↑ OD** | **----** |
| **Simpkins (2026)** | **H295R, HT-22, JEG-3, MCF-7**  **Adrenal, Liver, Placenta, Breast** | **0.3, 1, 3, 10, 30** | **MTT: NE ≤30 µM** | **----** | **----** | **NE (H_2_O_2_)** |
| **Robitaille (2015)** | **H295R**  **Human Adrenal Cancer** | **1, 3, 10, 30, 100** | **MTT: NE ≤100 µM** | **----** | **----** | **NE (CYP17)** |
| **Abarikwu (2011b)** | **PC12**  **Rat Adrenal Pheochromocytoma** | **232 µM**  **(24 hours)** | **NRU:** ↓  **TBE:** ↓  **LDH: ↑** | **MMP: ↑**  **@ 1, 6, 12 hrs** | **----** | **H_2_O_2_: ↑**  **MDA: ↑**  **GSH:** ↓ |
| **Abarikwu (2011c)** | **SH-SY5Y**  **Human Neuroblastoma** | **300 µM**  **(1, 6, 12, 24, 48 hours)** | **MTT:** ↓  **NRU:** ↓  **TBE:** ↓ | **MMP: ↑: 48 hrs** | **----** | **H_2_O_2_: ↑**  **MDA: ↑**  **GSH:** ↓ |
| **Powell (2011)** | **HepG2**  **Human hepatocellular carcinoma** | **0.4, 0.5, 0.7,1.4, 2.9, 5.8, 11.6, 23.2**  **48 or 72 hours** | **MTT:** ↓ **≥ 2.9 µM**  **72 hrs.** | **----** | **↑ S-Phase 0.5 uM**  **48 hrs.** | **----** |
| **Gao (2022)** | **L 8824**  **Grass Carp Hepatocytes** | **0.04, 0.16. 0.32, 0,64, 1.28, 2.59, 51.8**  **24 hours** | **CCK-8 Assay**  **Viability:** ↓ **≥ 0.64 µM** | **----** | **----** | **↑ ROS, MDA** |
| **Surapinit (2024)** | **MMNK-1**  **Human Cholangiocarcinoma (Bile Duct)** | **0.01, 0.1, 1, 10, 100**  **24, 48, 72 hours** | **MTT: ↑ 0.01 to 10 µM**  **MTT:** ↓ **100 µM** | **----** | **----** | **----** |
| **Surapinit (2024)** | **KKU-213B, KKU-055; KKU-100a**  **Human Intra-hepatic Cholangiocarcinoma** | **0.01, 0.1, 1, 10, 100**  **24, 48, 72 hours** | **MTT: ↑ 0.01 to 10 µM**  **MTT:** ↓ **100 µM**  **MTT:** ↓≥ **10 µM (KKU-100)** | **----** | **KKU-2103B & 055 ↑ G2/M @ 1 uM**  **↑ Migration @ 1 uM** | **----** |
| **Olejnik (2008)^b^** | **Caco-2**  **Human Colorectal adenocarcinoma** | **1, 10, 50, 100, 250**  **7, 14, 21, 28 days** | **MTT, OD: ↓ 10 µM**  **@ 14, 21, 28 days** | **----** | **----** | **----** |

**Abbreviations Used in Supplemental Table 6**

| **Cancer Cell Types** | **Cell Viability, Proliferation and Apoptosis Assays** |
| --- | --- |
|  | **CCK-8:** Colorimetric assay for cell viability or proliferation |
| **BG & 2008: Human Ovarian Cancer** | **KB**: Kenacid blue assay for cell protein content |
| **Caco-2: Human Colorectal adenocarcinoma** | **LDH**: Lactate Dehydrogenase Release Assay |
| **CAF: Cancer Associated Fibroblasts** | **MMP:** Mitochondrial membrane potential assay for apoptosis. |
| **CHO-K1, CCL-61: Chinese hamster ovary (epithelial)** | **MTT:** Cell Viability Assay |
| **GC: Quail Ovarian Granulosa cells** | **NRU**: Neutral Red Uptake Assay for cell viability |
| **HepG2: Human hepatocellular carcinoma** | **OD:** Optical Density (Indicator of cell density in vitro) |
| **H295R: Human Adrenal Cancer** | **SRB:** Sulfordhodame B assay for cell viability & proliferation |
| **H-22: Murine hepatocellular carcinoma** | **TBE**: Trypan Blue Exclusion assay for cell viability |
| **JEG-3: Human placental choriocarcinoma** | **WST-1 Colorimetric Assay:** cell proliferation, viability |
| **JKT-1: Human Seminoma cancer cells** | **Cell Proteins/Enzymes/Hormones/Endpoints** |
| **KKU-213B, KKU-055; KKU-100a Hepatic Human Cholangiocarcinoma** | **AREG**: Amphiregulin, an epithelial growth factor |
| **L 8824: Grass Carp Hepatocytes** | **CAT**: Catalase |
| **LNCaP: Human Prostate cancer cells** | **DHT:** Dihydroxytestosterone |
| **MCF-7 BUS: Human breast cancer cells** | **E2:** Estradiol |
| **MMNK-1 : Human Cholangiocarcinoma (Bile Duct** | **EREG:** Epiregulin, an epithelial growth factor |
| **PC12: Rat Adrenal Pheochromocytoma** | **FSH:** Follicle Stimulating Hormone |
| **RM1: Mouse Prostate cancer cells** | **GR:** Glutathione reductase |
| **SH-SY5Y: Human Neuroblastoma** | **GSH**: Glutathione |
| **4T1: Mouse Breast cancer cells** | **GSH-Px:** Glutathione peroxidase |
| **TM3: Mouse Leydig cells** | **GST:** Glutathione-s-transferase |
| **TM4: Mouse Sertoli cells** | **H_2_0_2_:** Hydrogen peroxide |
|  | **hCG:** Human chorionic gonadotropin |
|  | **LRH:** Luteinizing Hormone Receptor |
|  | **MDA**: Malondialdehyde (Lipid peroxidation by-product) |
|  | **PSA:** Prostate Specific Antigen |
|  | **PGR:** Progesterone Receptor |
|  | **P450 Enzymes:** CYP1A1/2, CYP2B, CYP17A1, CYP19A1 |
|  | **SF-1:** Steroidogenesis factor 1 |
|  | **Supraoxide Radical:** (O_2_^-^) |
|  | **SOD:** Supraoxide dismutase |
|  | **ROS:** Reactive Oxygen Species: H_2_O_2_, Supraoxide, Nitric Oxide |
|  | **Trx:** Thioredoxin |

**References for Supplemental Table 6**

Abarikwu, S. O., Farombi, E. O., Kashyap, M. P., & Pant, A. B. (2011a). Atrazine induces transcriptional changes in marker genes associated with steroidogenesis in primary cultures of rat Leydig cells. *Toxicol in Vitro, 25*(8), 1588-1595. doi:10.1016/j.tiv.2011.06.002

Abarikwu, S. O., Farombi, E. O., Kashyap, M. P., & Pant, A. B. (2011b). Kolaviron protects apoptotic cell death in PC12 cells exposed to atrazine. *Free Radic Res, 45*(9), 1061-1073. doi:10.3109/10715762.2011.593177

Abarikwu, S. O., Farombi, E. O., & Pant, A. B. (2011c). Biflavanone-kolaviron protects human dopaminergic SH-SY5Y cells against atrazine induced toxic insult. *Toxicol In Vitro, 25*(4), 848-858. doi:10.1016/j.tiv.2011.02.005

Abarikwu, S. O., Pant, A. B., & Farombi, E. O. (2013a). Quercetin decreases steroidogenic enzyme activity, NF-kappaB expression, and oxidative stress in cultured Leydig cells exposed to atrazine. *Mol Cell Biochem, 373*(1-2), 19-28. doi:10.1007/s11010-012-1471-z

Abarikwu, S. O., Pant, A. B., & Farombi, E. O. (2013b). Effects of quercetin on mRNA expression of steroidogenesis genes in primary cultures of Leydig cells treated with atrazine. *Toxicol In Vitro, 27*(2), 700-707. doi:10.1016/j.tiv.2012.11.005

Abarikwu, S. O., & Farombi, E. O. (2015). Atrazine induces apoptosis of SH-SY5Y human neuroblastoma cells via the regulation of Bax/Bcl-2 ratio and caspase-3-dependent pathway. *Pestic BioChem Physiol, 118*, 90-98. doi:10.1016/j.pestbp.2014.12.006.

Albanito, L., Lappano, R., Madeo, A., Chimento, A., Prossnitz, E. R., Cappello, A. R., . . . Maggiolini, M. (2015). Effects of atrazine on estrogen receptor alpha- and G protein-coupled receptor 30-mediated signaling and proliferation in cancer cells and cancer-associated fibroblasts. *Environ Health Perspect, 123*(5), 493–499. doi:10.1289/ehp.1408586

Chevalier, N., Paul-Bellon, R., & Fenichel, P. (2016a). Comment on "Effects of Atrazine on Estrogen Receptor alpha- and G Protein-Coupled Receptor 30-Mediated Signaling and Proliferation in Cancer Cells and Cancer-Associated Fibroblasts". *Environ Health Perspect, 124*(4), A64-65. doi:10.1289/ehp.1510927

Chevalier, N., Paul-Bellon, R., & Fenichel, P. (2016b). L’effet prolifératif des xéno-estrogènes ne peut pas être systématiquement déduit de celui observé avec le 17béta-estradiol, même à structure chimique apparentée : le modèle du cancer germinal testiculaire. *Ann Endocrinol (Paris), 77*(4), 321. doi:10.1016/j.ando.2016.07.220

Fa, S. Pogrmic-Majkic, K. Samardzija, D. Glisic, B. Kaisarevic, S. Kovacevic, R., and Andric, N. (2013). Involvement of ERK1/2 signaling pathway in atrazine action on FSH-stimulated LHR and CYP19A1 expression in rat granulosa cells. Toxicol Appl Pharmacol. 270 (1), 1-8. DOI: 10.1016/j.taap.2013.03.031

Farombi, E. O., Abarikwu, S. O., Adesiyan, A. C., & Oyejola, T. O. (2013). Quercetin exacerbates the effects of subacute treatment of atrazine on reproductive tissue antioxidant defence system, lipid peroxidation and sperm quality in rats. *Andrologia, 45*(4), 256-265. doi:10.1111/and.12001

Gao, M., Zhu, H., Guo, J., Lei, Y., Sun, W., & Lin, H. (2022). Tannic acid through ROS/TNF-alpha/TNFR 1 antagonizes atrazine induced apoptosis, programmed necrosis and immune dysfunction of grass carp hepatocytes. *Fish Shellfish Immunol, 131*, 312-322. doi:10.1016/j.fsi.2022.09.062

Hu, K., Tian, Y., Du, Y., Huang, L., Chen, J., Li, N., . . . Zhao, L. (2016). Atrazine promotes RM1 prostate cancer cell proliferation by activating STAT3 signaling. *Int J Oncol, 48*(5), 2166-2174. doi:10.3892/ijo.2016.3433

Kmetic, I., Gaurina Srcek, V., Slivac, I., Simic, B., Kniewald, Z., & Kniewald, J. (2008). Atrazine exposure decreases cell proliferation in Chinese Hamster Ovary (CHO-K1) cell line. *Bull Environ Contam Toxicol, 81*(2), 205-209. doi:10.1007/s00128-008-9425-6

Li, X. W., Yi, B. J., Wang, Z. Y., Guo, K., Saleem, M. A. U., Ma, X. Y., . . . Li, J. L. (2024). The ROS/SIRT1/STAR axis as a target for melatonin ameliorating atrazine-induced mitochondrial dysfunction and steroid disorders in granulosa cells. *EcoToxicol Environ Saf, 269*, 115780. doi:10.1016/j.ecoenv.2023.115780

Mgbudom-Okah, C. J., Abarikwu, S. O., Wegwu, M. O., & Henkel, R. (2024). Testosterone restores TM3 and TM4 cell viability, reduces reactive oxygen species generation, and protects against atrazine-induced stereological changes in rat testes. *J Biochem Mol Toxicol, 38*(1), e23562. doi:10.1002/jbt.23562

Oh, S. M., Shim, S. H., & Chung, K. H. (2003). Antiestrogenic action of Atrazine and its major metabolites in vitro. *J Health Sci, 49*(1), 65-71.

Olejnik, A., Marecik, R., Skrzypczak, M., Czaczyk, K., & Grajek, W. (2008). Application of rapid Caco-2 cell culture system in the studies on the bacterial adhesion and transepithelial transport. *Pol J Food Nutr Sci, 58*(3), 365-371. Retrieved from <http://journal.pan.olsztyn.pl/fd.php?f=1083>

Pogrmic, K., Fa, S., Dakic, V. Kaisarevic S., and Kovacevic R. (2009). Atrazine oral exposure of peripubertal male rats downregulates steroidogenesis gene expression in Leydig cells. Toxicol Sci. 111 (1)189-97. DOI: kfp135 [pii]10.1093/toxsci/kfp135

Pogrmic-Majkic, K., Fa, S., Dakic, V., Kaisarevic S., and Kovacevic R. (2010). Up-regulation of peripubertal rat Leydig cell steroidogenesis following 24 hour in vitro and in vivo exposure to atrazine. Toxicol Sci 118 (1), 52-60 DOI: 10.1093/toxsci/kfq227

Pogrmic-Majki, K., Kaisarevic, S., Fa, S., Dakic V., Glisic, B., Hrubik, J., and Kovacevic, R., (2012). Atrazine effects on antioxidant status and xenobiotic metabolizing enzymes after oral administration in peripubertal male rat. Environ Toxicol Pharmacol 34 (2) 495-501. DOI: 10.1016/j.etap.2012.06.004

Pogrmic-Majkic, K., Samardzija, D., Fa, .S. Hrubik, J.. Glisic, b.. Kaisarevic, S., and Andric, N. (2014). Atrazine enhances progesterone production through activation of multiple signaling pathways in FSH-stimulated rat granulosa cells: evidence for premature luteinization. Biol Reprod. 91 (5), 127. DOI: 10.1095/biolreprod.114.122606

Pogrmic-Majkic, J., Fa, S., Samardzija, D., Hrubik, J. Kaisarevic, S., and Andric, N. (2016). Atrazine activates multiple signaling pathways enhancing the rapid hCG-induced androgenesis in rat Leydig cells. Toxicology 2016 Vol. 368-369 Pages 37-45. DOI: 10.1016/j.tox.2016.08.016

Pogrmic-Majkic, K. Samardzija, D. Stojkov-Mimic, N., Vukosavljevic, J., Trninic-Pjevic, A. Kopitovic, V., and Andric, N., (2018). Atrazine suppresses FSH-induced steroidogenesis and LH-dependent expression of ovulatory genes through PDE-cAMP signaling pathway in human cumulus granulosa cells. Mol Cell Endocrinol. 461, 79-88 DOI: 10.1016/j.mce.2017.08.015

Powell, E. R., Faldladdin, N., Rand, A. D., Pelzer, D., Schrunk, E. M., & Dhanwada, K. R. (2011). Atrazine exposure leads to altered growth of HepG2 cells. *Toxicol In Vitro, 25*(3), 644-651. doi:S0887-2333(11)00003-8 [pii] 10.1016/j.tiv.2011.01.001

Robitaille, C. N., Rivest, P., & Sanderson, J. T. (2015). Antiandrogenic mechanisms of pesticides in human LNCaP prostate and H295R adrenocortical carcinoma cells. *Toxicol Sci, 143*(1), 126-135.

Simpkins, JW, Cooper, JC, and Breckenridge, C (2025). Quantitative *in vitro* to *in vivo* Extrapolation (QIVIVE) of atrazine’s effect on phosphodiesterase and aromatase mRNA expression *in vitro* to the rat *in vivo* based o*n* pharmacokinetic data. Submitted, In Vitro Toxicology.

Surapinit, A., Chaidee, A., Pinlaor, S., Kongsintaweesuk, S., Charoenram, N., Mahaamnad, N., . . . Hongsrichan, N. (2024). Atrazine promotes cholangiocarcinoma cell proliferation and migration via GPER-mediated PI3K/Akt/NF-κB pathway. *Pestic BioChem Physiol, 203*, 105988. doi:10.1016/j.pestbp.2024.105988

Wang, M., Chen, J., Zhao, S., Zheng, J., He, K., Liu, W., . . . Zhao, L. (2023). Atrazine promotes breast cancer development by suppressing immune function and upregulating MMP expression. *EcoToxicol Environ Saf, 253*, 114691. doi:10.1016/j.ecoenv.2023.114691

Yuan, B., Liang, S., Jin, Y. X., Zhang, M. J., Zhang, J. B., & Kim, N. H. (2017). Toxic effects of atrazine on porcine oocytes and possible mechanisms of action. *PLoS One, 12*(6), e0179861. doi:10.1371/journal.pone.0179861
