# Supplementary material for: Review of the effect of atrazine on the HPG axes and steroidogenic pathways in males: relevance for testicular and prostate cancer
Source: Front Toxicol. 2026 Mar 11;7:1702389. doi: 10.3389/ftox.2025.1702389 (PMC13012850; doi:10.3389/ftox.2025.1702389)
Supplement: Supplementary file 15 [file Supplementaryfile4.docx]

**Supplemental Figure 4: Effect of Atrazine or Stress on Basal (Panel B) or Angiotensin II – Induced Aldosterone Plasma Concentration (Panel A)**

| **Panel A:** Groups of seven young adult (60 to 90 days of age) female Sprague- Dawley rats were administered atrazine at a dose of 100 mg/kg/day for 1, 2, 3, or 4 consecutive days.  On the last day of treatment, dexamethasone was given before the last dose to suppress the atrazine-induced ACTH response, which would normally lead to an increase in plasma aldosterone (See Panel B).  Ninety minutes after dexamethasone dosing, the last atrazine dose was administered, or 30 minutes of restraint stress was applied to the restraint-stress group of animals.  Forty-five minutes post-treatment, the animals were anaesthetized with 4% isoflurane, and a baseline blood sample (Time Zero) was taken from an indwelling jugular catheter that had been implanted under anesthesia.  Angiotensin II (5 µg/kg body weight) was injected into the cannula, and blood samples were taken every 10 minutes post-Angiotensin II injection.  A group of sham-treated animals was handled identically except that they were administered the vehicle used to solubilize atrazine.  The group of restraint-stressed animals was treated identically to the sham-treated group, except they had 30 minutes of restraint stress applied before anesthesia.  Panel A displays the group mean (± SEM) plasma aldosterone concentrations during baseline and 10, 20, 30, or 40 minutes post-Angiotensin II administration.  Aldosterone plasma concentration was significantly elevated in the atrazine-treated group compared to sham controls 10 minutes post-Angiotensin II injection. | Panel B: Atrazine was administered to groups of ten male Sprague-Dawley rats/group at doses of 0, 6.5, 25, or 100 mg/kg/day for 1, 7, 14, or 28 days. Group mean (±SEM) plasma concentrations of aldosterone indicated that the high dose of atrazine induced a statistically significant increase 30 minutes after the first atrazine dose but not after 7, 14, or 28 doses. A significant reduction in aldosterone levels on day 28 was observed in the 25 and 100 atrazine-treated groups and the pair-fed control group. |
| --- | --- |
