# Supplementary material for: Review of the effect of atrazine on the HPG axes and steroidogenic pathways in males: relevance for testicular and prostate cancer
Source: Front Toxicol. 2026 Mar 11;7:1702389. doi: 10.3389/ftox.2025.1702389 (PMC13012850; doi:10.3389/ftox.2025.1702389)

Supplemental Figure 3: Absence of c-Fos Expression in the PVN of Atrazine-Treated Males (Foradori et al., 2018)

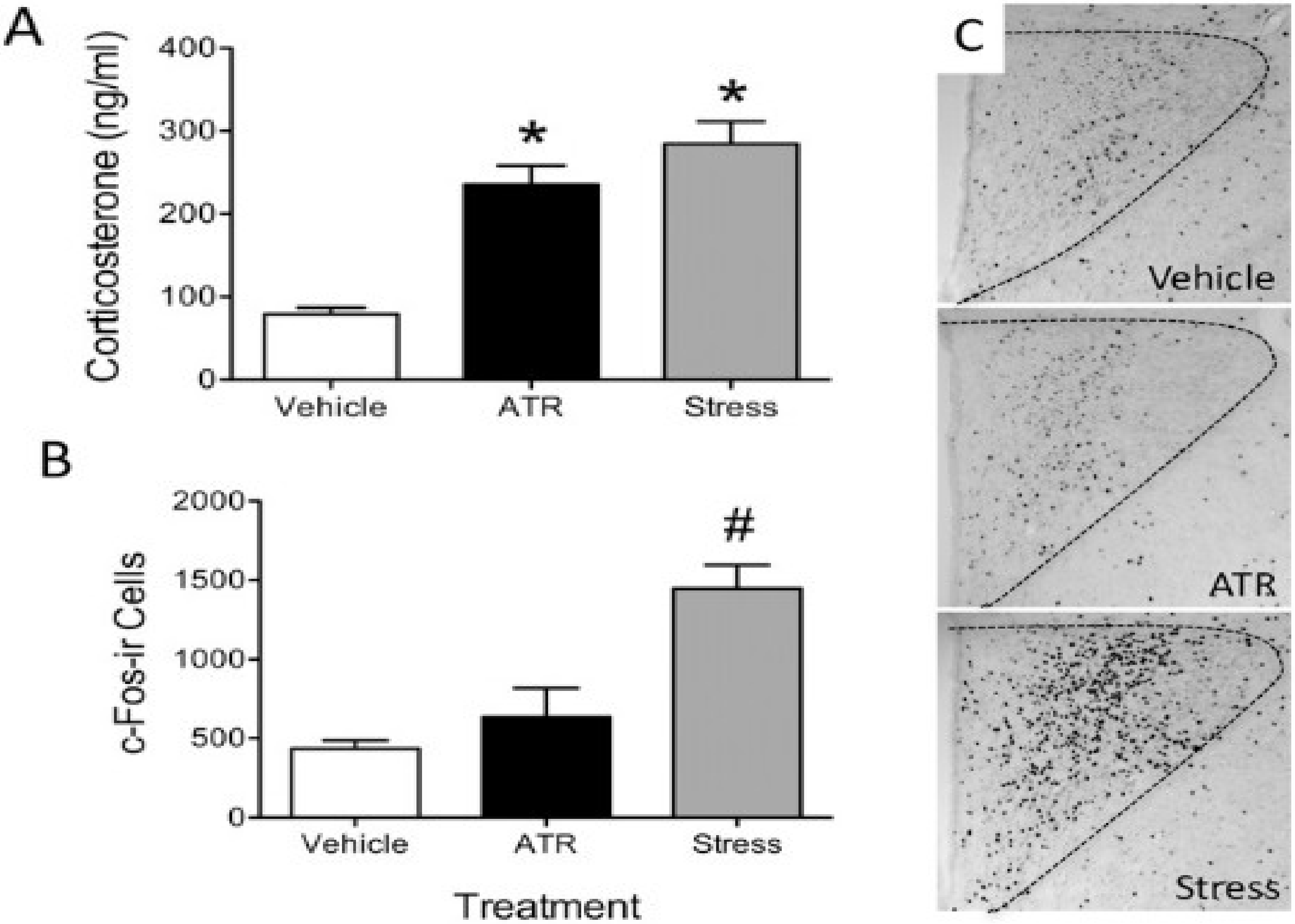

Supplement: Supplementary file 15 [file Supplementaryfile3.docx]
